# A small molecule antagonist of SMN disrupts the interaction between SMN and RNAP II

**DOI:** 10.1038/s41467-022-33229-5

**Published:** 2022-09-16

**Authors:** Yanli Liu, Aman Iqbal, Weiguo Li, Zuyao Ni, Yalong Wang, Jurupula Ramprasad, Karan Joshua Abraham, Mengmeng Zhang, Dorothy Yanling Zhao, Su Qin, Peter Loppnau, Honglv Jiang, Xinghua Guo, Peter J. Brown, Xuechu Zhen, Guoqiang Xu, Karim Mekhail, Xingyue Ji, Mark T. Bedford, Jack F. Greenblatt, Jinrong Min

**Affiliations:** 1 Jiangsu Key Laboratory of Neuropsychiatric Diseases and College of Pharmaceutical Sciences, Soochow University, Suzhou, Jiangsu China; 2 Hubei Key Laboratory of Genetic Regulation and Integrative Biology, School of Life Sciences, Central China Normal University, Wuhan, Hubei China; 3 Structural Genomics Consortium, University of Toronto, Toronto, ON Canada; 4 Donnelly Centre, University of Toronto, Toronto, ON Canada; 5 Department of Epigenetics and Molecular Carcinogenesis, The University of Texas MD Anderson Cancer Center, Houston, TX USA; 6 Department of Laboratory Medicine and Pathobiology, Faculty of Medicine, University of Toronto, Toronto, ON Canada; 7 Life Science Research Center, Southern University of Science and Technology, Shenzhen, Guangdong China; 8 Department of Physiology, University of Toronto, Toronto, ON Canada

**Keywords:** X-ray crystallography, Small molecules, Methylation, Transcription

## Abstract

Survival of motor neuron (SMN) functions in diverse biological pathways via recognition of symmetric dimethylarginine (Rme2s) on proteins by its Tudor domain, and deficiency of SMN leads to spinal muscular atrophy. Here we report a potent and selective antagonist with a 4-iminopyridine scaffold targeting the Tudor domain of SMN. Our structural and mutagenesis studies indicate that both the aromatic ring and imino groups of compound **1** contribute to its selective binding to SMN. Various on-target engagement assays support that compound **1** specifically recognizes SMN in a cellular context and prevents the interaction of SMN with the R1810me2s of RNA polymerase II subunit POLR2A, resulting in transcription termination and R-loop accumulation mimicking *SMN* depletion. Thus, in addition to the antisense, RNAi and CRISPR/Cas9 techniques, potent SMN antagonists could be used as an efficient tool to understand the biological functions of SMN.

## Abstract (web summary)

The SMN protein recognizes symmetric dimethylarginine by its Tudor domain, and SMN deficiency leads to spinal muscular atrophy. Here, Liu et al. discover a small molecule that binds to the SMN Tudor domain and disrupts the interaction between SMN and RNA Polymerase II.

## Introduction

**Fig. 1:**
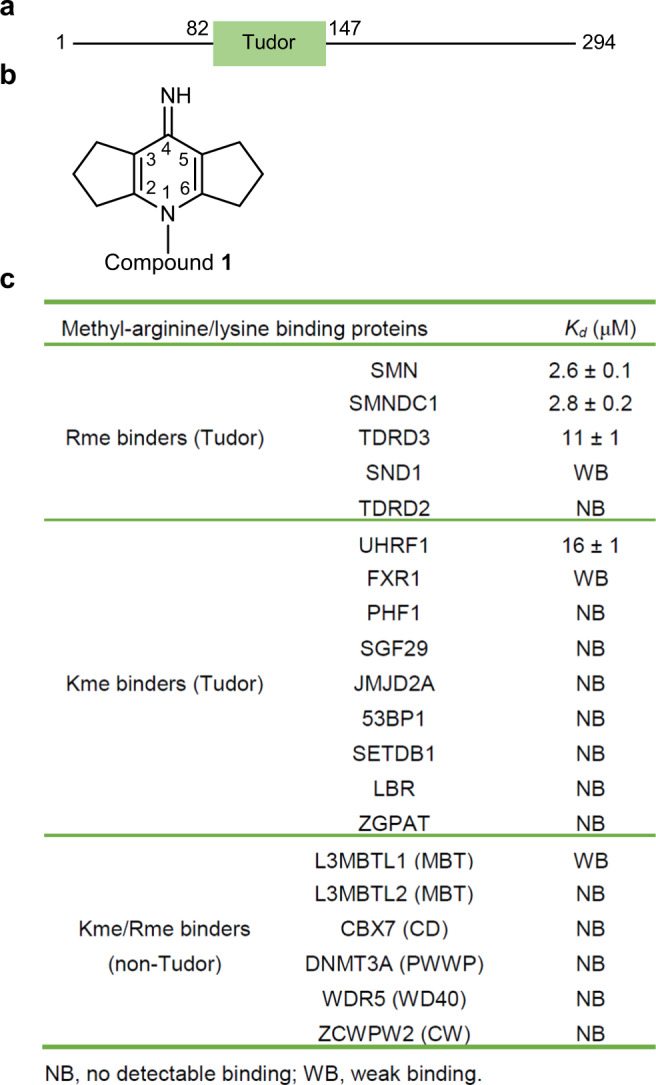
Compound **1** preferentially binds to SMN among assayed methylarginine or methyllysine binders. **a** Domain structure of SMN. **b** Molecular structure of compound **1**. **c** Binding affinities of compound **1** to selected modified histone readers measured by ITC. ITC data shown are representative of two independent experiments. The names of non-Tudor domains were shown in the parentheses. Source data are provided as a Source Data file.

Survival of motor neuron (SMN), a Tudor domain-containing protein, is a core component of the SMN complex, which is essential for biogenesis of small nuclear ribonucleoproteins (snRNPs) by assembling the heptameric Sm ring onto spliceosomal snRNA^1^. The Tudor domain of SMN (Fig. 1a) binds to arginine symmetric-dimethylated (Rme2s) Sm proteins, and this interaction plays a critical role in snRNP assembly^2,3^. Considering the importance of SMN in the fundamental process of snRNP assembly, it is not surprising that complete loss of SMN is lethal. The human genome contains 2 genes, *SMN1* and *SMN2*, which produce the identical SMN protein. Homozygous deletion or mutation of *SMN1* coupled with a single nucleotide substitution at position 6 of exon 7 (C6T) of *SMN2* is responsible for spinal muscular atrophy (SMA)^4^, the most common genetic cause of infant death with a frequency of 1 in ~10,000 births^5^.

In addition to its role in snRNP assembly, SMN is also involved in regulation of nuclear architecture^6,7^, local axonal translation in neurons^8^, and transcription termination^9^. SMN regulates nuclear architecture by interacting with arginine methylated coilin, a Cajal body (CB) specific protein. Cajal bodies (CBs) and gemini of Cajal bodies (Gems) are the twin subcellular organelles in the nucleus of proliferative cells such as embryonic cells, or metabolically active cells such as motor neurons. Coilin harbors symmetrically dimethylated arginine residues^6,7^. Sufficient arginine methylation of coilin is required for its binding to SMN, which is stored in Gems and accompanies snRNP to CBs during differentiation of the human neuroblastoma cell line SH-SY5Y^10^. SMN is also reported to regulate local axonal translation via the miR-183/mTOR pathway in neurons^8^. Specifically, the miR-183 level is increased and local axonal translation of mTor is reduced in SMN-deficient neurons. In an SMA mouse model, suppression of the miR-183 expression in the spinal motor neurons strengthens motor function and increases survival of *Smn*-mutant mice, which uncovers another potential mechanism responsible for SMA pathology^8^. SMN also interacts with symmetric-dimethylated R1810 at the C-terminal domain (CTD) of RNA polymerase II (RNAP II) subunit POLR2A (R1810me2s-POLR2A) via its Tudor domain to regulate transcription termination^9^. In SMA patients, abnormal transcription termination such as pause of RNAP II and R-loop (DNA-RNA hybrids) accumulation in the termination region may facilitate neurodegeneration^9^. Taken together, SMN functions in different biological pathways, and the Tudor domain of SMN plays a critical role in executing these functions by mediating arginine methylation-dependent interactions. In spite of the extensive study of SMN and its associated SMA disease, it is still unclear how SMN protects motor neurons in the spinal cord against degeneration.

In this work, we set out to design SMN-selective chemical probes that would specifically occupy the methylarginine binding pocket and disrupt the Tudor domain-mediated and arginine methylation-dependent interactions. These SMN-specific chemical probes could be used to better understand biological functions of SMN in different pathways and molecular etiology of SMA.

## Results

### Identification of an SMN-selective small molecule antagonist

In this study, we obtained an SMN-selective antagonist by serendipity when we tried to screen inhibitors against the histone H3K9me3 binding tandem Tudor domain (TTD) of UHRF1 (Fig. 1b, Supplementary Fig. 1a and Supplementary Table 1). In this fluorescence-based peptide displacement screen for UHRF1, we found 5 hits, among which compound **1** was confirmed by isothermal titration calorimetry (ITC) (*K*_*d*_ ~16 µM, Supplementary Fig. 1b, c). As we know, many proteins bind to lysine and arginine methylated histones/proteins, including the Tudor Royal superfamily (Tudor, chromodomain, PWWP and MBT) of proteins and some CW-type (cystine and tryptophan) and PHD-type (plant homeodomain) zinc finger containing proteins^11–14^, and all of these proteins utilize an aromatic cage to recognize the methyllysine or methylarginine residue. In order to investigate the binding selectivity of compound **1**, we screened it against selected methylarginine or methyllysine-binding Tudor domains and methyllysine/methylarginine-binding non-Tudor domains (Fig. 1c). UHRF1_TTD was the only assayed methyllysine binder that bound to compound **1** measurably, and compound **1** bound more tightly to the methylarginine binding Tudor domains of SMN, SMNDC1, and TDRD3 than to the methyllysine binding Tudor domain of UHRF1_TTD (Fig. 1c and Supplementary Fig. 2). SMN, SMNDC1 and TDRD3 are the only three known methylarginine binding proteins of single canonical Tudor domain. Moreover, the highly homologous Tudor domains of SMN and SMNDC1 bound to compound **1** with a ~4-fold selectivity over that of TDRD3 (Fig. 1c).

### Compound 1 specifically engages SMN in a cellular context

**Fig. 2:**
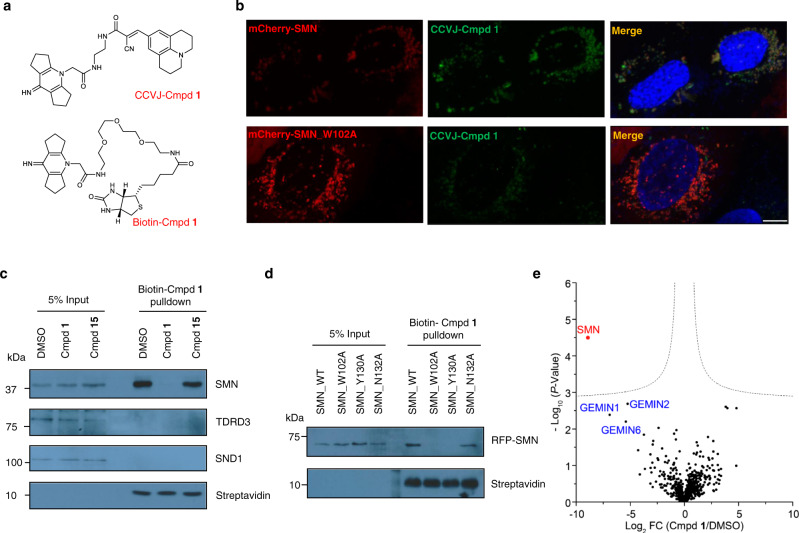
Cellular on-target engagement of compound **1**. **a** Chemical structure of CCVJ conjugated compound **1** (CCVJ-Cmpd **1**) and biotin conjugated compound **1** (biotin-Cmpd **1**). **b** mCherry-SMN (red) colocalizes with CCVJ-Cmpd **1** (green), which is lost when the cage residue W102 is mutated in SMN. Scale bar: 10 μm. **c** Compound **1**, but not negative compound **15**, prevents the pulldown of SMN by biotin-labeled compound **1** from cell lysates. **d** SMN cage mutants disrupt or weaken the interaction between SMN and biotin-Cmpd **1** in cell lysates. The U2OS cell lysate was incubated with 20 μM of biotin-Cmpd **1** overnight at 4 °C in figures **c** and **d**. Data shown are representative of three independent experiments in (**b**–**d**). **e** On-target engagement of compound **1** was analyzed by chemical proteomics. Volcano plot shows significantly displaced proteins from immobilized biotin-Cmpd **1** pulldowns by competition with 200 μM compound **1** relative to DMSO (FDR *q* value = 0.01, S0 = 0. 1, two-tailed Student’s *t*-test and *n* = 3 biological replicates). Significantly depleted protein colored and labeled in red, major potential prey proteins labeled in blue. FC: fold change. Source data are provided as a Source Data file.

To verify the cellular on-target engagement of compound **1**, we subcloned *SMN* into the mammalian expression vector mCherry2-C1 to express the N-terminally mCherry tagged SMN fluorescent protein, and conjugated compound **1** to 9-(2-carboxy-2-cyanovinyl)julolidine (CCVJ), a fluorescent molecular rotor as previously reported^15^ to generate CCVJ-Cmpd **1**, which presents switched-on fluorescence upon binding to SMN, or to biotin to generate a biotin conjugate compound **1** (biotin-Cmpd **1**) for cellular lysate pulldown assays^16^ (Fig. 2a and Supplementary Fig. 3). Our ITC results showed that these two modified compounds still bound to SMN (Supplementary Fig. 4). When the fluorescence-switching CCVJ-Cmpd **1** binds to SMN, restriction of the fluorescent molecule rotations would trigger emission of strong green fluorescence signals^15^, which is confirmed in solution (Supplementary Fig. 5). Upon treatment of U2OS cells with CCVJ-Cmpd **1**, the green fluorescent compound **1** colocalized with the red fluorescent mCherry-SMN, which was not observed with the SMN cage mutant (Fig. 2b), indicating that compound **1** binds to the aromatic cage of SMN specifically.

To further confirm the cellular binding of compound **1** to SMN, we performed pulldown assays of cell lysates by using the biotin-labeled compound **1** (biotin-Cmpd **1**). Our results showed that SMN could be efficiently captured, while neither TDRD3 nor SND1 could be detected (Fig. 2c and Supplementary Fig. 6). The biotin-Cmpd **1** could be competed out by the presence of unlabeled compound **1**, but not the negative control compound **15** in the lysates (Fig. 2c). Furthermore, the biotin-Cmpd **1** could not efficiently pull down the SMN cage mutants (Fig. 2d). Affinity-purification and mass spectrometry (AP-MS) based proteomic analysis of the biotin-Cmpd **1** pulldown samples identified SMN as the most significant protein target (with the largest fold change, log_2_ FC = −8.9). Their interaction was efficiently blocked by the competition of compound **1** (Fig. 2e). The second to forth AP-MS potential prey proteins are GEMIN1 (log_2_ FC = −6.9), GEMIN6 (log_2_ FC = −5.4) and GEMIN2 (log_2_ FC = −5.2), the three major components of the SMN complex^17,18^, further indicating that compound **1** selectively and specifically binds to endogenous SMN in the cells. Taken together, these data provide convincing evidence that compound **1** binds to the full-length SMN protein specifically in a cellular context.

### Structural basis of selective compound 1 binding to SMN

**Fig. 3:**
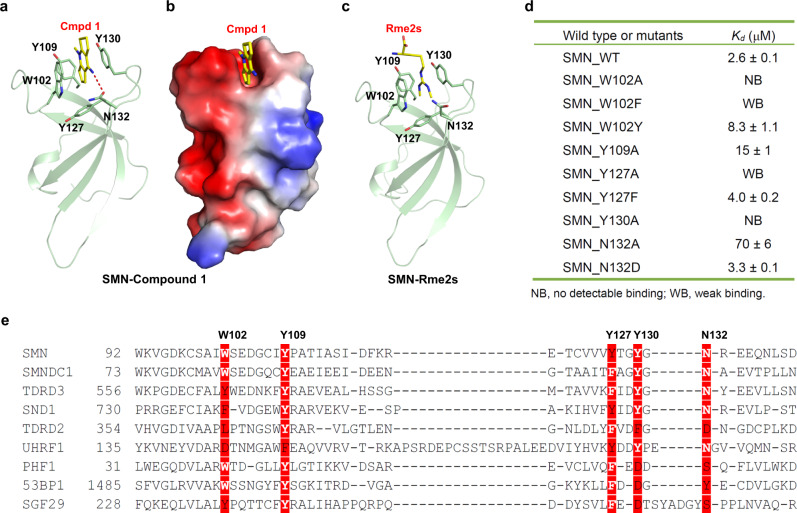
Structural basis of preferential binding of compound **1** to SMN. **a** Cartoon mode of the complex structure of Tudor domain of SMN and compound **1**. The Tudor domain of SMN was colored in green, with the interacting residues shown in sticks and the intermolecular hydrogen bonds indicated by red dashes. **b** Electrostatic potential surface representation of the complex of Tudor domain of SMN and compound **1**. **c** Cartoon mode of the complex structure of Tudor domain of SMN and Rme2s. **d** Binding affinities of compound **1** to different SMN Tudor mutants determined by ITC. Shown are representative of two independent experiments. **e** Sequence alignment of selected Tudor domains. The compound **1** interacting residues were highlighted in red background. Structure figures were generated in PyMOL. Surface representations were calculated with the built-in protein contact potential function of PyMOL. Source data are provided as a Source Data file.

In order to understand the structural basis of compound **1** recognition by these reader proteins, we determined the crystal structures of compound **1** in complex with SMN, TDRD3 and UHRF1, respectively (Fig. 3, Supplementary Figs. 7–9 and Table 1). In the complex structure of SMN-compound **1**, compound **1** binds to the aromatic cage formed by W102, Y109, Y127 and Y130, which otherwise accommodates dimethylarginine of its physiological ligands (Fig. 3a–c and Supplementary Fig. 7a, b). W102 and Y130 sandwich compound **1** rings. In addition, compound **1** forms a hydrogen bond between its imino group and the side chain of N132. This hydrogen bond boosts the ligand binding ability of SMN, because mutating N132 to alanine significantly reduced its binding affinity (Fig. 3d).

**Table 1:** Data collection and refinement statistics

Complex	SMN-Cmpd 1	SMN-Cmpd 4	SMN-Cmpd 6	UHRF1-Cmpd 1	TDRD3-Cmpd 1
**PDB code**	4QQ6	7W2P	7W30	4QQD	6V9T
**Data reduction**					
Space group	P6_5_	P4_1_2_1_2	P322_1_	P6_1_	H3_2_
**Cell dimensions**					
* a*, *b*, *c* (Å)	27.8, 27.8, 112.8,	35.9, 35.9, 92,	70.7, 70.7, 119.9,	87.3, 87.3, 83.9,	83.9, 83.9, 114.0,
α, β, γ (°)	90, 90, 120	90, 90, 90	90, 90, 120	90, 90, 120	90, 90, 120
Resolution (Å)	37.61–1.75 (1.79–1.75)	46.00–1.15 (1.17–1.15)	35.36–1.80 (1.84–1.80)	41.96–2.28 (2.36–2.28)	44.85–2.15 (2.22–2.15)
*R*_merge_	0.078 (0.806)	0.078 (0.991)	0.063 (1.308)	0.093 (0.944)	0.104 (0.982)
No. of Reflections	5006 (283)	22319 (1064)	32704 (1857)	16676 (1642)	8558 (703)
Mean *I*/σ	19.8 (2.9)	21.5 (2.2)	29.4 (2.0)	21.9 (2.9)	18.9 (2.6)
CC_1/2_	0.999 (0.825)	0.999 (0.785)	1.000 (0.774)	0.999 (0.801)	0.998 (0.884)
Completeness (%)	100.0 (100.0)	100.0 (99.6)	99.4 (96.6)	100.0 (100.0)	99.8 (98.0)
Multiplicity	10.5 (10.3)	13.4 (10.7)	10.5 (10.2)	11.3 (11.4)	11.1 (11.1)
**Model refinement**					
Resolution (Å)	24.07–1.75	33.44–1.15	35.36–1.80	41.96–2.28	44.85–2.15
No. of reflections work / free	4942/508	22243/1144	32536/1243	16644/1208	8557/699
*R*_work_/*R*_free_	0.165/0.242	0.126/0.152	0.210/0.258	0.200/0.250	0.193/0.232
No. of atoms / mean *B*-factor (Å^2^)	505/27.1	623/13.6	2031/32.2	2292/41.4	961/47.0
Protein	462/26.8	549/12.9	1827/32.3	2163/41.8	877/47.1
Inhibitor	14/29.8	21/9.2	65/31.1	70/31.4	70/48.7
Water	26/31.0	38/21.4	110/32.3	54/37.6	11/36.4
RMSD bonds (Å) / angles (°)	0.017/1.7	0.026/2.5	0.016/1.9	0.014/1.4	0.016/2.0

Values in parentheses are for the highest resolution shell.

In the TDRD3-compound **1** structure, the binding mode is largely conserved (Supplementary Figs. 7c, d and 8), but Y566 of TDRD3 might not stack with the compound as effectively as W102 in SMN (Fig. 3e and Supplementary Fig. 10), which may explain weaker binding affinity of TDRD3 to compound **1** (Fig. 1c). Consistent with this hypothesis, the W102Y mutant of SMN showed a binding affinity to compound **1** similar to wild-type TDRD3, and the Y566W mutant of TDRD3 displayed a binding affinity to compound **1** similar to wild-type SMN (Fig. 3d and Supplementary Fig. 11). Although we did not determine the corresponding complex structure of SND1, the structure-based sequence alignment revealed that W102 of SMN corresponds to F740 of SND1 (Fig. 3e and Supplementary Fig. 10). Consistent with this, the W102F mutant of SMN only weakly bound to compound **1**, while the F740W mutant of SND1 displayed a significantly enhanced binding affinity to compound **1** (Fig. 3d and Supplementary Fig. 11). In addition, the W102A and Y130A mutants of SMN did not bind to compound **1** (Fig. 3d). This lack of binding is consistent with our failure to observe binding for the other tested proteins. On the other hand, the mutations of the SMN cage residues Y109 and Y127 weakened, but did not abrogate the binding of SMN to compound **1** (Fig. 3d). Hence, the sandwich stacking interactions of compound **1** by W102 and Y130 of SMN play a more critical role in the specific compound **1** recognition by SMN.

### UHRF1 recognizes compound 1 via an arginine-binding pocket

To uncover the specific interactions between UHRF1_TTD and compound **1**, we also solved the crystal structure of UHRF1_TTD in complex with compound **1** (Supplementary Figs. 7e, f and 9). Two UHRF1_TTD molecules are present in each asymmetric unit of the UHRF1-compound **1** complex structure (Supplementary Fig. 9a), but we only observed the expected disc-shaped electron density of compound **1** in the histone H3K9me3-binding cage of one UHRF1_TTD molecule, while we found a differently shaped blob in the histone H3K9me3-binding cage of the other UHRF1_TTD molecule (Supplementary Fig. 9b). In the complex structure of UHRF1_TTD-PHD and the H3K9me3 peptide (PDB code: 3ASK), an arginine residue R296 in the linker between the TTD and PHD domains of UHRF1 is found in a pocket formed by D142, E153, A208, M224, W238 and F278 from the TTD domain^19^ (Supplementary Figs. 1a and 9c). R296 is locked in the pocket by forming a salt bridge with D142. Intriguingly, we found the disc-shaped density that resembles compound **1** in the R296-binding pockets of both UHRF1_TTD molecules, and compound **1** is stacked between the indole ring system of W238 and the guanidinium group of R209 in both UHRF1_TTD molecules (Supplementary Fig. 9b, d).

Several lines of evidence suggested that the R296-binding pocket is the major binding site and the methyllysine-binding aromatic cage is just a minor or non-specific binding site. First, when we mutated the aromatic cage residues of UHRF1_TTD that have been shown to be critical for histone H3K9me3 binding to alanine, the binding affinity of compound **1** is not affected significantly. In contrast, when we mutated the R296-binding pocket residues, the binding is totally disrupted (Supplementary Fig. 9e). Second, the electron density inside the H3K9me3 aromatic cage is either smear and can be modeled in multiple orientations of compound **1**, which implies that compound **1** does not bind to the cage specifically, or is of no defined density shape (Supplementary Fig. 9b). Third, the aromatic cage has a propensity to accommodate small molecules non-specifically. For instance, some molecules in buffer have been found in the aromatic cage of TDRD3^20^. For the case of UHRF1_TTD, some ethylene glycol molecules from the crystallization buffer are found in the H3K9me3 aromatic cage and the R296-binding pocket of the apo-UHRF1_TTD structure^21^ (Supplementary Fig. 9f). Fourth, in the SMN-compound **1** complex structure, compound **1** is stacked between the aromatic rings of W102 and Y130. However, the three aromatic residues in the aromatic cage of UHRF1 are perpendicular to each other, which could not stack the compound like SMN does (Supplementary Fig. 9g). Taken together, UHRF1 used the R296-binding pocket to specifically bind to compound **1**, and this R296-binding pocket could serve as a therapeutic venue for designing potent small molecule allosteric regulators of the UHRF1 functions. Based on the structural information we obtained from our UHRF1_TTD-compound **1** complex and the UHRF1_TTD-PHD-H3K9me3 complex (PDB code: 3ASK), it is conceivable that the compound **1** binding pocket of UHRF1 is occupied by R296 of the full-length UHRF1 protein, which would prevent compound **1** from chemiprecipitating UHRF1 from the U2OS cell lysate.

### The imino group of compound 1 plays a critical role in binding to SMN

To explore if we could identify more potent compounds than compound **1**, we procured commercially available analogs of compound **1** that include single, double and triple ring molecules and measured their binding affinities to SMN or UHRF1, respectively (Table 2). SMN bound to all the four triple-ring compounds with affinities between 2.6 and 31 μM (Table 2 and Supplementary Fig. 12). The four triple-ring compounds have a 4-iminopyridine scaffold in common, and none of them is more potent than the original hit. Although the binding affinities of SMN and these compounds are not high, SMN binds to these compounds much stronger than its physiological ligands such as symmetric dimethylarginine or R1810me2s-POLR2A, which showed a binding affinity of 476 μM^22^ or 175 μM^9^, respectively.

**Table 2:** Binding affinities of compound 1 analogs reveal the importance of the triple-ring and imino group of compound 1

Compound	Structure	SMN	UHRF1
		*K*_*d*_ (µM)	
**1**		2.6 ± 0.1	16 ± 1
**2**		9.3 ± 0.6	No binding
**3**		31 ± 2	No binding
**4**		13 ± 1	No binding
**5**		12 ± 1	No binding
**6**		12 ± 2	Weak binding
**7**		No binding	No binding
**8**		No binding	No binding
**9**		No binding	No binding
**10**		No binding	No binding
**11**		No binding	No binding
**12**		No binding	No binding
**13**		No binding	No binding
**14**		No binding	No binding
**15**		No binding	No binding
**16**		No binding	No binding
**17**		No binding	No binding
**18**		No binding	No binding
**19**		No binding	No binding
**20**		No binding	No binding

ITC data shown are representative of two independent experiments. Source data are provided as a Source Data file. *K*_*d*_: binding affinity.

Due to the electronic similarity between the N-methyl and 4-imino sites on the 4-iminopyridine core, we did not expect to resolve the orientation of compound **1** based on electron density alone, and could not exclude the possibility that the imino group would instead protrude into the solvent. However, the positive binding results of 1-substituted pyridine cores to SMN presented here could confirm that N132 does interact with the imino group and substituents on the pyridine nitrogen would point away from the Tudor domain, as larger 1-substituents would otherwise clash inside the aromatic cage. In addition, the modifications at the N-methyl site of compound **1**, such as CCVJ-Cmpd **1** and biotin-Cmpd **1**, did not disrupt the interaction of SMN and the modified compounds, which further validates our statement that SMN binds to the 4-imino group of compound **1** to entail a hydrogen bond between the 4-imino group and N132. Indeed, our crystal structure of SMN in complex with compound **4** also confirms that the imino group of compound **4** forms a hydrogen bond with N132 (Supplementary Figs. 7g, h and 13a, b).

In addition to triple-ring compounds **1** to **4**, SMN also bound to two of twelve double-ring compounds, compounds **5** and **6** (Table 2 and Supplementary Fig. 12). Both compounds retain the imino group, which pinpoints the importance of the imino group-mediated hydrogen bond in the compound binding and is consistent with our crystal structures of SMN in complex with compounds **1**, **4**, and **6** (Fig. 3 and Supplementary Figs. 7 and 13). None of the single-ring compounds bound to SMN, which may not be able to provide strong enough π–π stacking interaction to hold the compounds. UHRF1, however, did not bind to any other three-ring compounds, because the substituents on the pyridine nitrogen of these compounds are too large to accommodate the more enclosed arginine-binding pocket of UHRF1.

### SMN antagonists disrupt the SMN-RNAP II interaction

**Fig. 4:**
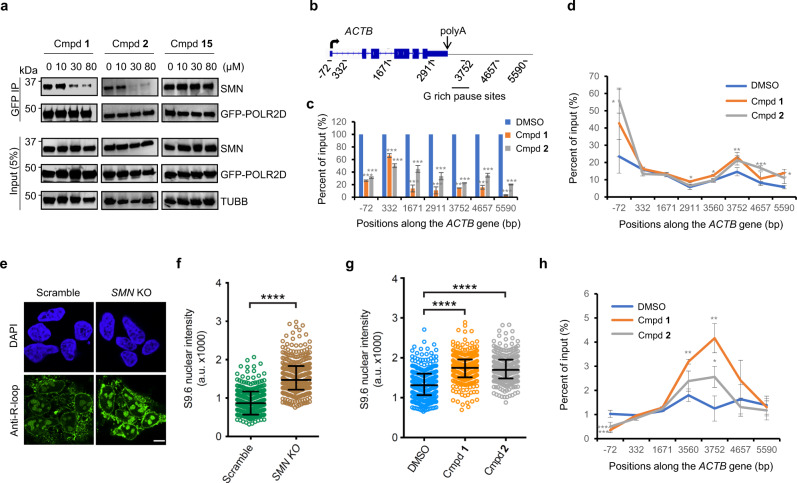
Effects of SMN antagonists on the interaction between SMN and RNAP II, RNAP II pause, and R-loop accumulation. **a** SMN antagonists disrupt binding of SMN to RNAP II. Data shown are representative of three independent experiments. **b** Illustration of *ACTB* gene and the qPCR amplification positions. **c** SMN antagonists reduce SMN association at *ACTB* gene locus. Quantification of SMN qPCR data from ChIP experiments using SMN antibody at the indicated *ACTB* amplification positions. The SMN levels in DMSO controls were set as 100%. **d** SMN antagonists lead to RNAP II pause. Quantification of RNAP II qPCR data from ChIP experiments using POLR2A antibody at the indicated *ACTB* amplification positions. **e** *SMN* knockout (KO) leads to R-loop accumulation. Representative single-plane images of Z-stacks of the R-loop levels in scramble vs *SMN* KO cells of three independent experiments. Scale bar: 5 μm. **f** Global nuclear R-loop accumulation in *SMN* KO cells. **g** SMN antagonists cause global nuclear R-loop accumulation. **h** SMN antagonists lead to R-loop accumulation at *ACTB* gene locus. Quantified DNA immunoprecipitation using primers along *ACTB* locus by using GFP antibody, in cell extracts that were transfected with GFP-RNase H1 R-loop-binding domain (GFP-HB) fusion construct for R-loop detection. **a**–**h** HEK293 cells; **c**, **d**, **h** data were presented as the mean ± S.E.M. of three independent experiments (*P < 0.05, **P < 0.01, ***P < 0.001 for the two-tailed Student’s *t*-test); **f**, **g** scatter plots representing data from single-cell and R-loop immunofluorescence analysis (number of cells = 377, 332, 295, 262, 265 for scramble, *SMN* KO, DMSO, Cmpd **1**, Cmpd **2** condition, respectively; Mean ± Quartiles; ****P < 0.0001 for the two-tailed Mann–Whitney test; a.u.: arbitrary units). Source data are provided as a Source Data file.

We previously showed that R1810 in the CTD of the mammalian RNAP II subunit POLR2A is symmetrically dimethylated by PRMT5 and the R1810 methylated CTD directly recruits the Tudor-domain protein SMN, which contributes to the assembly of an R-loop resolving complex on the RNAP II CTD^9^. Hence, we asked whether these small molecule antagonists might be able to disrupt the interaction of SMN with RNAP II in cells. To test this possibility, we treated the HEK293 cells with a series of concentrations of either compound **1**, compound **2** or negative control compound **15** for 72 h, and then performed immunoprecipitation using the cell extracts. We demonstrated that coimmunoprecipitation of SMN with GFP fusion protein of POLR2D, a component of RNAP II, was inhibited by compound **1** and compound **2** on a dose-dependent manner, but not by negative compound **15** or DMSO (Fig. 4a). Furthermore, the coimmunoprecipitation of POLR2A with SMN was also disrupted on the treatment of compound **1** and compound **2**, whereas no significant effect was observed for the cells treated with DMSO as a control (Supplementary Fig. 14). These results provide convincing evidence that the small molecule antagonists compound **1** or compound **2** of SMN disrupts the interaction between SMN and the RNAP II complex. Since 20 µM of either compound **1** or compound **2** is enough to exhibit significant inhibition of the interaction between SMN and POLR2A (Supplementary Fig. 14), we used this concentration in the following assays.

### SMN antagonists disrupt SMN gene occupancy and lead to RNAP II pause

Our previous ChIP study has shown that SMN occupies the *ACTB (β-actin)* gene from its promoter to the termination regions with the highest level of occupancy at the 3′-end of the gene^9^. PRMT5 depletion or POLR2A R1810 mutation leads to a decreased SMN occupancy^9^. To examine whether the small molecule antagonists of SMN have any effects on the SMN occupancy at its target genes during transcription, the SMN ChIP assay was performed using the primers along the *ACTB* gene (Fig. 4b). Similar to the effects of PRMT5 depletion or POLR2A R1810 mutation, treatment of either compound **1** or compound **2** significantly reduced the occupancy levels of SMN along the *ACTB* gene (Fig. 4c). Given that POLR2A CTD R1810A mutation or depletion of *SMN* leads to the accumulation of RNPA II at genes^9^, the SMN antagonists might have similar effects. To explore this possibility, we performed RNAP II ChIP experiments and found that addition of either compound **1** or compound **2** (20 µM, 72 h) significantly increased the occupancy levels of RNAP II at the promoter regions and 3′-end of the *ACTB* gene as detected by quantitative PCR (qPCR) (Fig. 4d). These results indicate that the SMN antagonists could cause the accumulation of RNAP II at both the promoter and 3′ pause site of its target genes.

### SMN depletion or its inhibition causes R-loop accumulation

Our previous studies demonstrated that PRMT5 or SMN depletion, or POLR2A R1810 mutation leads to R-loop accumulation at the *ACTB* gene^9^. Here, we further confirmed that CRISPR/Cas9 mediated *SMN* knockout increased global R-loop accumulation in HEK293 cells as detected by immunofluorescence staining (Fig. 4e, f and Supplementary Fig. 15). Overexpression of RNase H1 significantly decreased the levels of R-loops in the *SMN* knockout cells, validating the authenticity of the R-loop signals (Supplementary Fig. 16). Similar to the *SMN* knockout, treatment with either compound **1** or compound **2** significantly increased R-loop levels in comparison to the DMSO controls (Fig. 4g), indicating the global effects of SMN antagonists in R-loop accumulation. Consistently, treatment of either compound **1** or compound **2** significantly increased R-loop signals at the 3′-end of the *ACTB* gene (Fig. 4h).

## Discussion

In this study, we identified some low micromolar antagonists with a 4-iminopyridine scaffold targeting the Tudor domain of SMN, and compound **1** shows >4-fold selectivity over other tested methyllysine or methylarginine binding domains. Although the binding affinity of SMN and compound **1** is not high, SMN binds to compound **1** 60–180-fold more tightly than its physiological ligands such as symmetric dimethylarginine or R1810me2s-POLR2A (*K*_*d*_ of 2.6 μM for compound **1** vs 476 μM for symmetric dimethylarginine^22^ or 175 μM for R1810me2s-POLR2A^9^). We then utilized different cellular on-target engagement assays to validate that compound **1** specifically recognizes SMN in a cellular context, and showed that compound **1** would prevent the interaction of SMN with R1810me2s of DNA-directed RNA polymerase II subunit POLR2A and result in transcription termination and R-loop accumulation. Hence, compound **1** is a potent and selective antagonist of SMN.

Our structural and mutagenesis studies provide mechanistic insights into the selectivity of compound **1** for SMN. Our protein-compound complex structures uncover that compound **1** is an antagonist targeting methylated arginine binding protein and the sandwich stacking interactions of compound **1** by W102 and Y130 of SMN play a critical role in the compound **1** recognition. The larger binuclear ring structure of tryptophan provides a stronger π–π interaction with compound **1** than tyrosine or phenylalanine. In order to explore if mutating Y130 to tryptophan would increases its binding to compound **1** further, we made a Y130W mutant, which renders the protein to become insoluble, presumably due to steric clash around the aromatic cage. In addition, our structural study uncovers that UHRF1 used an arginine-binding pocket to specifically bind to compound **1**, which indicates that the arginine-binding pocket could serve as a therapeutic venue for designing potent small molecule allosteric regulators of the UHRF1 functions.

Although the causative link between SMN deficiency and SMA was established 20 years ago^4^, it remains elusive how deficiency of a protein, which is ubiquitously expressed and causes widespread defects in pre-mRNA splicing in cell culture and mouse models of SMA, would result in a cell-type-specific phenotype: motor neuron dysfunction^23^. A *Drosophila* model suggests that involvement of SMN in snRNP biogenesis does not explain locomotion and viability defects of *Smn* null mutants, implying that SMN may have other functions contributing to the etiology of SMA^24^. Indeed, in addition to its role in snRNP assembly, SMN is also involved in regulation of nuclear architecture by interacting with arginine methylated coilin^6,7^, local axonal translation in neurons by participating in miR-183/mTOR pathway^8^, and transcription termination by interacting with arginine methylated POLR2A^9^. All of these findings may have important implications for understanding the cell-specific functions of SMN, and shed light on the molecular mechanism of SMA pathology^6–8,10^. SMN was also proposed to have other functions. It interacts with the mSin3A/HDAC transcription corepressor complex and thus represses transcription in an HDAC-dependent manner^25^. In contrast, by interacting with the nuclear transcription activator E2 of papillomavirus, SMN positively regulates E2-dependent transcription^26^. The Tudor domain of SMN recognizes arginine methylated Epstein-Barr virus nuclear antigen 2 (EBNA2), the main viral transactivator of Epstein-Barr virus (EBV)^4^, and regulates EBV-mediated B-cell transformation^27^. Infection with the EBV can lead to a number of human diseases including Hodgkin’s and Burkitt’s lymphomas. SMN interacts with the fused in sarcoma (FUS) protein, a genetic factor in amyotrophic lateral sclerosis, which links the two motor neuron diseases^28^.

In order to facilitate the elucidation of the biological functions of SMN in different pathways and molecular etiology of SMA, we set out to develop SMN-specific chemical probes, and identified compound **1**, a 2.6 μM antagonist. Although compound **1** could not be claimed as a chemical probe, compound **1** and even weaker binding compound **2** bind to SMN much stronger than its physiological ligand R1810me2s-POLR2A, and our cellular studies still display that these SMN antagonists prevent SMN interaction with R1810me2s-POLR2A, resulting in the over-accumulation of active RNAP II and R-loop, mimicking depletion of *SMN*. These small molecule compounds specifically compete with methylated arginine for the binding pocket of SMN. Application of these small molecules has the advantage of maintaining the normal cellular SMN levels without disrupting methylarginine independent functions of SMN. Thus, in addition to the antisense, RNAi and CRISPR/Cas9 techniques, these potent SMN antagonists may be used as efficient tools in the study of SMN biology and its related neurological diseases.

## Methods

### Protein expression and purification

The coding DNA fragments of following Tudor domains were cloned into pET28-MHL vector: SMN (aa 82–147), UHRF1 (aa 126–285), SMNDC1 (aa 53–130), TDRD3 (aa 554–611), SND1 (aa 650–910), TDRD2 (aa 327–420), FXR1 (aa 2–132), PHF1 (aa 28–87), SGF29 (aa 115–293), JMJD2A (aa 897–1101), 53BP1 (aa 1483–1606), SETDB1 (aa 190–410), LBR (aa 1–67), ZGPAT (aa 120–271). The coding regions of chromodomain of CBX7 (aa 8–62), PWWP domain of DNMT3A (aa 275–417), WD40 repeats of WDR5 (aa 24–334) and CW domain of ZCWPW2 (aa 21–78) were also subcloned into pET28-MHL vector to generate N-terminally His-tagged fusion protein. The MBT repeats of L3MBTL1 (aa 200–522) and L3MBTL2 (aa 170–625) were subcloned into pET28GST-LIC vector to generate N-terminally GST-His-tagged fusion protein. All the plasmids were generated by ligase independent cloning (Vazyme Biotech, C112 or ABclonal Technology, RK21020). The recombinant proteins were overexpressed in *E*. *coli* BL21 (DE3) Codon plus RIL (Stratagene, 230280) at 15 °C under induction of 0.25 mM IPTG (isopropyl-β-D-thiogalactoside) and purified by affinity chromatography on Ni-nitrilotriacetate resin (Qiagen, or Nanjing Qingning Bio-Technology Co., Ltd.) followed by TEV (for the N-terminally His-tagged fusion protein) or thrombin (for the N-terminally GST-His-tagged fusion protein) protease treatment to cleave the tag. The buffer condition for Ni-affinity chromatography is as following: lysis buffer: 20 mM Tris-HCl, pH 7.5, 250 mM NaCl, 5% glycerol and 5 mM β-mercaptoethanol; wash buffer: 20 mM Tris-HCl, pH 7.5, 1 M NaCl and 40 mM imidazole; elution buffer: 20 mM Tris-HCl, pH 7.5, 250 mM NaCl and 250 mM imidazole; dialysis buffer: 20 mM Tris-HCl, pH 7.5, 150 mM NaCl and 5 mM β-mercaptoethanol. The proteins were further purified by Superdex75 or Superdex200 gel-filtration column (GE Healthcare) in a buffer containing 20 mM Tris-HCl, pH 7.5, 150 mM NaCl and 1 mM DTT. For crystallization experiments, purified proteins were concentrated to 18 mg/mL for SMN, 23 mg/mL for UHRF1 and 10 mg/mL for TDRD3 in the gel-filtration buffer. All the mutations were introduced with the QuikChange II XL site-directed mutagenesis kit (Stratagene, 200522) and confirmed by DNA sequencing. Mutant proteins were also expressed in *E. coli* BL21 (DE3) Codon plus RIL and purified using the same procedures described above. The molecular weight of all protein samples was measured by mass spectrometry.

For mammalian expression, the coding DNAs of full-length SMN, SND1 and TDRD3 were cloned into mCherry2-C1 or GFP-C1 vector through digestions with restriction endonucleases *Hind* III/*Bam*H I and ligation with T4 DNA ligase. All the mutations of full-length SMN were introduced with the QuikChange II XL site-directed mutagenesis kit (Stratagene, 200522) and confirmed by DNA sequencing. All the primers used in this research were shown in Supplementary Table 2.

### Small molecule fragment-based screening of UHRF1 tandem Tudor domain

A small molecule fragment library with 2040 compounds was screened against TTD of UHRF1 by fluorescein polarization-based peptide displacement assay according to previous reports^29^. Briefly, the screening was performed in 10 μL at a protein concentration of 8 μM premixed with a 40 nM FITC-labeled H3K9me3 peptide (aa 1–25, Tufts University Core Services), and then adding a single concentration of 2 mM compound in a buffer of 20 mM Tris-HCl, pH 8.8, 50 mM NaCl, and 0.01% Triton X-100. The hits were further confirmed by dose response analysis with 1 mM as the highest concentration with 11 sequential 2-fold dilutions. All the assays were performed in duplicate in 384-well plates (Greiner, 784290), using the Synergy 4 microplate reader (BioTek), with an excitation wavelength of 485 nm and an emission wavelength of 528 nm. Data were corrected by background of the free labeled peptides and analyzed by GraphPad Prism version 5 software. All the compounds were purchased from Maybridge, Sigma or Specs company.

### Chemical synthesis and compound characterization

Synthesis of CCVJ and biotin conjugated compound **1** (CCVJ-Cmpd **1** and biotin-Cmpd **1)** and their characterization are described in the Supplementary Methods.

### Isothermal titration calorimetry (ITC)

For the ITC measurement, the concentrated proteins were diluted into 20 mM Tris-HCl, pH 7.5, 150 mM NaCl (ITC buffer); the lyophilized compounds were dissolved in the same buffer, and the pH value was adjusted by adding 2 M NaOH or 2 M HCl. The compounds that could not be dissolved in the ITC buffer were dissolved in DMSO with the accessible highest concentration. Compound concentrations were calculated from the mass and the volume of the solvent. For the ITC assay with compound dissolved in DMSO, the protein was diluted by ITC buffer containing same final concentration of DMSO. All measurements were performed in duplicate at 25 °C, using a VP-ITC (MicroCal, Inc.), an iTC-200 (MicroCal, Inc.), or a Nano-ITC (TA, Inc.) microcalorimeter. The protein with a concentration of 50–100 μM was placed in the cell chamber, and the compounds with a concentration of 0.5–2 mM in syringe was injected in 25, 20, or 20 successive injections with a spacing of 180, 150, or 120 s for VP-ITC, iTC-200, or Nano-ITC, respectively, as previously described^30–32^. iTC-200 or Nano-ITC data were consistent with those from the VP-ITC instrument, based on ITC results of SMN-compound **1** detected by using all the instruments. Control experiments were performed under identical conditions to determine the heat signals that arise from injection of the compounds into the buffer. Data were fitted using the single-site binding model within the Origin software 7.0 package (MicroCal, Inc.) or the independent model within the Nano-Analyze software package (TA, Inc.).

### Protein crystallization

For the complex crystal of SMN-compound **1**, SMN was crystallized in a buffer containing 2 M ammonium sulfate, 0.2 M potassium/sodium tartrate, 0.1 M sodium citrate, pH 5.6, and soaked with compound **1** at a molar ratio of 1:5 for 24 h. For the complex crystals of UHRF1-compound **1**, SMN-compound **4**/**6,** and TDRD3-compound **1**, purified proteins were mixed with the compounds at a molar ratio of 1:5 and co-crystallized using the sitting drop vapor diffusion method at 18 °C by mixing 0.5 μL of the protein with 0.5 μL of the reservoir solution. The complex of UHRF1-compound **1** was crystallized in a buffer containing 20% PEG 3350, 0.2 M magnesium nitrate; SMN-compound **4** was crystallized in a buffer containing 1.8 M sodium acetate, pH 7.0, 0.1 M Bis-Tris propane, pH 7.0; SMN-compound **6** was crystallized in a buffer containing 2 M ammonium sulfate, 0.2 M sodium chloride, 0.1 M HEPES, pH 7.5; and TDRD3-compound **1** was crystallized in a buffer containing 1.2 M sodium citrate, 0.1 M Tris-HCl, pH 8.5. Before flash-freezing crystals in liquid nitrogen, crystals were soaked in a cryoprotectant consisting of 85% reservoir solution and 15% glycerol.

### Data collection and structure determination

The program PHASER^33^ was used for molecular replacement (MR) when needed. Models were interactively rebuilt, refined and validated using COOT^34^, REFMAC^35^ and MOLPROBITY^36,37^ software, respectively. MarvinSketch (Chemaxon.com) was used for the calculation of some SMILES strings during preparation of small molecule geometry restraints. PDB_EXTRACT^38^ and CCTBX^39^ library were used during preparation of the crystallographic models for PDB deposition and publication. Diffraction data and model refinement statistics for the structures are displayed in Table 1. Some structure determination details for specific structures are as follows. *SMN in complex with compound* **1**: Diffraction images were collected on a copper rotating anode source and initially reduced to merged intensities with DENZO/SCALEPACK^40^/AIMLESS^41^. For later refinement steps, data were reduced with XDS^42^/AIMLESS. The crystal structure was solved by placement of atomic coordinates from isomorphous PDB entry 1MHN^43^ in the asymmetric unit. Geometry restraints for compound **1** were prepared on the GRADE server^44,45^. *SMN in complex with compound* **4**: Diffraction data were collected at APS/NE-CAT beam line 24-ID-E and reduced with XDS/AIMLESS. The structure was solved by MR with diffraction data from an additional, isomorphous crystal and coordinates from PDB entry 4QQ6 (SMN in complex with compound **1**, above). Geometry restraints for compound **4** were prepared with PRODRG^46^. Anisotropic displacement parameters were analyzed on the PARVATI server^47^. *SMN in complex with compound* **6**: Diffraction data were collected on a rotating copper anode source and reduced with XDS/AIMLESS. The structure was solved by MR with coordinates from PDB entry 4QQ6. Geometry restraints for compound **6** were prepared with ELBOW^48^, which in turn used MOGUL. *UHRF1 in complex with compound* **1**: Diffraction data were collected at APS/SBC-CAT beamline 19ID and reduced to merged intensities with XDS/AIMLESS. The structure was solved by MR with coordinates derived from PDB entry 3DB3^49^. *TDRD3 in complex with compound* **1**: Diffraction data were collected at CLS/CMCF beamline 08ID and reduced to intensities with DENZO/SCALEPACK. Intensities were converted to the MTZ format with COMBAT^50^ or, alternatively, POINTLESS^51^ before symmetry-related intensities were merged with AIMLESS. The structure was solved by MR with coordinates from PDB entry 3PMT^20^.

### Fluorescence analysis

U2OS cells (ATCC, HTB-96) were plated in a 35 mm FluoroDish with a 0.17 mm coverslip bottom (World Precision Instruments, FD35-100) for 12–24 h and transfected with 1.0 μg mCherry-SMN WT (wild-type) or mutant plasmids. Media was changed 4–6 h after transfection and cells were cultured for another 24 h. 10 μM CCVJ-Cmpd **1** was added for 24 h treatment. Then the treated cells were rinsed by PBS and the media replaced with phenol-free FluoroBright DMEM (Thermo Fisher, A1896701) for analysis by using Zeiss LSM880 microscopy.

### Pulldown and western blotting

U2OS cells were lysed in RIPA buffer (140 mM NaCl, 10 mM Tris-HCl, pH 7.6, 1% Triton, 0.1% sodium deoxycholate, 1 mM EDTA) containing protease inhibitors (Roche, 05892791001). The cell lysate was incubated with 20 μM biotin-Cmpd **1** overnight at 4 °C, then 30 μL streptavidin beads (Thermo Fisher, 20353) was added and incubated at 4 °C for 1 h. The beads were then washed with RIPA buffer for 3 times, 10 min/time and loading buffer was added and boiled for 5 min for elution. The eluted samples were separated by SDS-PAGE for western blotting (SMN monoclonal antibody, BD Transduction Laboratories, 610646, clone No. 8, dilution of 1:1000; TDRD3 rabbit monoclonal antibody, Cell signaling, 5492, clone No. 5492, dilution of 1:1000; SND1 polyclonal antibody, Bethyl, A302-883A, dilution of 1:2000; RFP polyclonal antibody, Abcam, ab62341, dilution of 1:1000; GFP monoclonal antibody, Santa Cruz Biotechnology, sc-9996, dilution of 1:1000; Streptavidin-Horseradish Peroxidase (HRP), Invitrogen, SA10001, dilution of 1:5000) as previously described^52,53^. For the competition analysis, 200 μM biotin-free compound **1** or compound **15** was added at the same time when the biotin-Cmpd **1** was incubated with the cell lysates.

### Affinity-purification and mass spectrometry (AP-MS)

The samples for AP-MS were prepared following pulldown procedure as previously described with minor modifications^54^. Briefly, U2OS cells were lysed in RIPA buffer and the cell lysate was equally divided into two groups: (1) DMSO group (control group): the cell lysate was incubated with 20 μM biotin-labeled compound **1**; (2) Cpmd **1** group (sample group): the cell lysate was incubated with 20 μM biotin-labeled compound **1** and 200 μM free compound **1**. Each group contains three biological replicates. They were incubated overnight at 4 °C, then 30 μL streptavidin beads (Thermo Fisher, 20353) was added and incubated at 4 °C for 1 h. The beads were then washed with RIPA buffer for 3 times, 10 min/time.

Beads were rinsed twice with 50 mM TEAB, pH 7.55, before eluting proteins with 25 μL of 5% SDS, 50 mM TEAB, pH 7.55. The sample was then centrifuged at 17,000 × *g* for 10 min to remove any debris. Proteins were reduced with 20 mM TCEP (Thermo Fisher, 77720) and incubated at 65 °C for 30 min. The sample was cooled to room temperature and 1 μL of 0.5 M iodoacetamide acid was added and allowed to react for 20 min in dark. Phosphoric acid (12%, 2.75 μL) was added to the protein solution, followed by adding 165 μL of binding buffer (90% methanol, 100 mM TEAB, pH 7.1). The resulting solution was added to S-Trap spin column (protifi.com) and passed through the column using a bench top centrifuge (30 s spin at 4000 × *g*). The spin column was washed with 400 μL of binding buffer and centrifuged. This step was repeated two more times. Then trypsin was added to the protein mixture at a mass ratio of 1:25 in 50 mM TEAB, pH 8.0, and the sample was incubated at 37 °C for 4 h. Peptides were eluted with 80 μL of 50 mM TEAB, followed by 80 μL of 0.2% formic acid, and finally 80 μL of 50% acetonitrile, 0.2% formic acid. The combined peptide solution was then dried in a speed vac and resuspended in 2% acetonitrile, 0.1% formic acid, 97.9% water and placed in an autosampler vial.

Samples were analyzed by nanoLC-MS/MS (nanoRSLC, Thermo Fisher) using an Aurora series (Ion Opticks) reversed phase HPLC column (25 cm length × 75 µm inner diameter) and directly injected to an Orbitrap Eclipse (Thermo Fisher) using a 120 min gradient (mobile phase A = 0.1% formic acid, mobile phase B = 99.9% acetonitrile with 0.1% formic acid; hold 2% B for 5 min, 2–6% B in 0.1 min, 6–25% in 100 min, 25–50% in 15 min) at a flow rate of 350 nL/min. Eluted peptide ions were analyzed using a data-dependent acquisition (DDA) method with resolution settings of 120,000 and 15,000 (at m/z 200) for MS1 and MS2 scans, respectively. DDA-selected peptides were fragmented using stepped high energy collisional dissociation (27, 32, and 37%).

The data of AP-MS were analyzed as a previously described method with minor modifications^55^. Briefly, the raw MS files were searched with MaxQuant software (version 1.6.1.1, www.maxquant.org)^56^ against the UniProt human protein database (www.uniprot.org) concatenated with common contaminants and the decoy database. The mass tolerance for precursor ions was set to 20 ppm and 4.5 ppm for the first and main search, respectively. The cysteine carbamidomethylation was set as fixed modification and methionine oxidation and N-terminal acetylation as variable modifications. Enzyme specificity was set to trypsin and a maximum missed cleavage was set as 2. The 1% false discovery rate (FDR) at both peptide and protein levels was applied for the analysis. Relative protein quantification was based on the label-free quantification incorporated in the MaxQuant software. The iBAQ intensity of proteins was obtained for the control and experimental samples. The missing iBAQ intensity was replaced by a random number, which was calculated from a normal distribution with a width of 0.3 and a downshift of 1.8 defined by Perseus software (version 1.6.5.0). The *P*-value was calculated by performing two-sample Student’s *t*-test. Log_2_ FC (Cmpd **1**/DMSO) and −Log_10_ (*P*-value) from three biological replicates were used to construct the volcano plot using OriginPro 9.0.

### Cell culture

HEK293 cells (ATCC, CRL-1573) were grown in DMEM (SLRI media facility) plus 10% FBS (Sigma, F1051). For analysis of SMN chemical antagonists, HEK293 cells were treated with DMSO or a series of concentrations (0, 2, 6, 10, 20, 30, 40, and 80 μM) of compound **1**, compound **2** or negative compound **15** for 72 h. CRISPR-mediated *SMN1* gene knockout was performed according to our previous study^9^. Briefly, 2 μg of CRISPR/Cas9 plasmid (pCMV-Cas9-GFP), which expresses scrambled guide RNA, or guide RNA that targets the *SMN1* gene exon1 (gRNA target sequence: ATTCCGTGCTGTTCCGGCGCGG) or exon3 (gRNA target sequence: GTGACATTTGTGAAACTTCGGG) was transfected into HEK293 cells. Cells were sorted by BD FACSAria flow cytometry at Donnelly Center, University of Toronto 24 h after transfection, and single GFP-positive cells were seeded into a 48-well plate. The expression levels of SMN in each clone were detected by immunofluorescence. The transfection of GFP-RNase H1 R-loop-binding domain (GFP-HB) for R-loop detection into HEK293 cells was performed with the FuGENE Transfection reagent (Roche, E269A).

### Immunoprecipitation (IP) and western blotting

The experiments were performed following procedure as previously described with minor modifications^57^. Briefly, HEK293 cells were subjected to three freeze-thaw cycles in high-salt lysis buffer (10 mM Tris-HCl, pH 7.9, 10% glycerol, 420 mM NaCl, 0.1% Nonidet P-40, 2 mM EDTA, 2 mM DTT, 10 mM NaF, 0.25 mM Na_3_VO_4_, and 1× protease inhibitor mixture (Sigma, P8340)), followed by centrifugation at 18,400 × *g* for 1 h at 4 °C to remove insoluble materials. The supernatant cell lysates were sonicated with five on and off cycles of 0.3 s/0.7 s per mL and incubated for 30 min at 4 °C with 12.5–25 units/mL benzonase nuclease (Sigma, E1014) to remove RNA and DNA, followed by centrifugation at 18,400 × *g* for 30 min at 4 °C. The supernatant cell lysates were incubated with 2 μg of antibody overnight at 4 °C, followed by the addition of 20 μL of Dynabeads Protein G beads (Invitrogen, 10004D) for an additional incubation for 4 h. After washing with low-salt buffer (10 mM Tris-HCl, pH 7.9, 100 mM NaCl, and 0.1% Nonidet P-40) 3 times, 10 min/time, associated proteins were eluted into protein-loading buffer and separated by Tris 4–20% SDS-polyacrylamide (Mini-PROTEAN TGX Precast Protein Gel, BioRad, 4561096), and transferred to nitrocellulose or PVDF membranes (Immu-Blot PVDF, BioRad, 1620112 or 1620177). Transferred samples were immunoblotted with primary antibodies (POLR2A, Abcam, ab5408, monoclonal antibody, clone No. 4H8; SMN, Santa Cruz Biotechnology, sc-15320, polyclonal antibody; ACTB, Sigma, A5441, monoclonal antibody, clone No. AC-15; GFP, Invitrogen, G10362, rabbit monoclonal antibody; TUBB, Santa Cruz Biotechnology, sc-9104, polyclonal antibody) at a dilution of 1:2000 to 1:5000, followed by horseradish peroxidase-conjugated goat anti-mouse or mouse anti-rabbit secondary antibody (Jackson Immuno Research, 115-035-174 or 211-032-171) at a dilution of 1:10,000. Western blot detection was performed with enhanced chemiluminescence (Pierce ECL Western Blotting Substrate, Thermo Fisher, 32209). For analysis of SMN chemical antagonists, HEK293 cells were treated with DMSO, compound **1** or compound **2** with a series of concentrations for 72 h, before being processed for IP and western blotting.

### Chromatin immunoprecipitation (ChIP)

ChIP was performed using the EZ-ChIP™ A - Chromatin Immunoprecipitation Kit (Millipore, 17-371) according to the manufacturer’s instruction. Antibodies were used with a range of 1–2 μg, and IgG (Millipore, polyclonal antibody, 12-370) was used as a background control. After immunoprecipitation, genomic DNA was de-crosslinked in ChIP elution buffer containing 5 M NaCl at 65 °C overnight and purified with the Qiaex II kit (Qiagen, 20021), and eluted in water for PCR amplification. Immunoprecipitated and input DNAs were used as templates for qPCR. The qPCR primer sequences for *ACTB* gene are the same as described earlier^9^ and shown in the Supplementary Table 2. For analysis of SMN chemical antagonists, HEK293 cells were treated with DMSO, compound **1** or compound **2** (a final concentration of 20 μM) for 72 h, before processing for ChIP.

### Immunofluorescence and microscopic R-loop quantification

Global nuclear R-loop detection was ascertained via immunofluorescence using the S9.6 antibody (Kerafast, ENH001)^58^. At 24 h prior to immunofluorescence, 40,000 cells were seeded on to Poly-L-Lysine (PLL) coated coverslips. Cells were fixed using 1% formaldehyde for 15 min, washed three times with PBS, permeabilized with 0.3% Triton X-100 and washed again three times with PBS. Coverslips were blocked using 5% BSA for 1 h at room temperature and transferred to humidified chambers for antibody incubations. Coverslips were incubated with 60 μL of monoclonal S9.6 antibody (Kerafast, ENH001, 1:500) for 1 h at room temperature. After washing with PBS, cells were incubated with a secondary goat anti-mouse Alexa Fluor 488 antibody (Thermo Fisher, A11001, 1:1000) for 1 h in a dark chamber. Following further washing and DAPI staining, coverslips were mounted onto microscope slides using DAKO fluorescent mounting medium (Agilent, S302380-2) and then sealed with nail polish. For RNase H1 overexpression analysis, scramble and *SMN* knockout cells were seeded in 6-well plates and transfected 24 h later with 0.9 μg pcDNA3-Empty vector or pcDNA3-RNase H1. At 48 h post-transfection, cells were harvested and re-seeded onto PLL-coated coverslips. The coverslips were processed for immunofluorescence 24 h later using the primary antibodies monoclonal S9.6 (Kerafast, ENH001, 1:500), or polyclonal anti-RNase H1 (Proteintech, 15606-1-AP, 1:500) and the secondary antibodies goat anti-mouse Alexa Fluor 488 (Thermo Fisher, A11001, 1:1000) or goat anti-rabbit Alexa Fluor 568 (Thermo Fisher, A11011, 1:1000) to quantify R-loop and confirm RNase H1 overexpression, respectively. For the analysis of SMN chemical antagonists, HEK293 cells were treated with DMSO, compound **1** or compound **2** at a final concentration of 20 μM for 72 h before processing for S9.6 immunofluorescence.

We employed a Nikon C2+ confocal microscope coupled to NIS-elements AR software (Nikon). For R-loop microscopy in HEK293 cells, random fields identified by DAPI staining were captured at 100× magnification. For any given image, 5–6 2D imaging planes were acquired along the *z*-axis to generate 3D confocal image stacks. DAPI was used to stain nuclei and S9.6 intensity values for individual cells were obtained as maximum intensity planes via the NIS-elements AR software (Nikon). Representative single-plane images from z-stacks were adjusted for background and contrast in Photoshop (Adobe).

### Reporting summary

Further information on research design is available in the Nature Research Reporting Summary linked to this article.

## Supplementary information

Supplementary Information

Reporting Summary

## Data Availability

The coordinates and structure factors generated in this study have been deposited in the Protein Data Bank (PDB) with accession codes 4QQ6 (SMN-compound **1**), 4QQD (UHRF1-compound **1**), 7W2P (SMN-compound **4**), 7W30 (SMN-compound **6**), 6V9T (TDRD3-compound **1**). The mass spectrometry proteomics data generated in this study have been deposited to the ProteomeXchange Consortium via the iProX partner repository^59^ with the dataset identifier PXD034927 (Identification of the target proteins for compound **1** in U2OS cells). The structural data used in this study are available in the Protein Data Bank (PDB) under accession codes 1MHN^43^ (SMN Tudor domain structure), 3DB3^49^ (UHRF1-H3K9me3 complex), 3PMT^20^ (TDRD3 Tudor domain structure), 3ASK^19^ (UHRF1-H3K9me3 complex), 5YYA^21^ (UHRF1 bound to ethylene glycol). The uncropped and unprocessed versions of blots, all original ITC curves, synthesis and characterization of CCVJ and biotin conjugated compound **1** (CCVJ-Cmpd **1** and biotin-Cmpd **1**) generated in this study are provided in the Supplementary Information and the Source Data file. Source data are provided with this paper.

## Source data

Source Data
