# Supplementary material for: A small molecule antagonist of SMN disrupts the interaction between SMN and RNAP II
Source: Nat Commun. 2022 Sep 16;13:5453. doi: 10.1038/s41467-022-33229-5 (PMC9481570; doi:10.1038/s41467-022-33229-5)
Supplement: Supplementary file 1 — Supplementary Information [file 41467_2022_33229_MOESM1_ESM.pdf]

**A small molecule antagonist of SMN disrupts the interaction between SMN and RNAP II**

**Liu *et al.***

# Supplementary Information

**Supplementary methods**

Synthesis of CCVJ and biotin conjugated compound **1** (CCVJ-Cmpd **1** and biotin-Cmpd **1**) and the respective characterization.

**Chemistry**

**General**: All reagents and chemicals were reagent grade and were used without further purification. All reactions were carried out in flame-dried glassware and monitored by thin layer chromatography. NMR spectra were recorded on a INOVA NMR spectrometer at 400 MHz. Chemical shifts are reported in parts per million relative to tetramethylsilane as internal standard.

**The synthesis of CCVJ-Cmpd 1**

**1,2,3,5,6,7-hexahydropyrido[3,2,1-*ij*]quinoline-9-carbaldehyde (I-2):** Vilsmeier-Haack salt was synthesized by adding $POCl_3$ (1.06 g, 6.926 mmol) drop-wise to a dry round bottom flask containing DMF (0.506 g, 6.926 mmol) maintaining temperature at 0 °C under $N_2$ atmosphere for 1 hour. Later, a solution of 1,2,3,5,6,7-hexahydropyrido[3,2,1-*ij*]quinoline (**I-1**, 1.0 g, 5.771 mmol) in 5 mL of DMF was added to the above complex at a stretch. The resulting solution was stirred at RT for about 2 hours. The container was quenched to ice cold water while stirring. The solid obtained was filtered, washed with excess of water and then purified by column chromatographic technique using ethyl acetate and hexane as eluent to give compound **I-2** as brown solid. Yield: 0.9 g (77 %). $^1$H NMR (400 MHz, $CDCl_3$) δ 9.59 (s, 1H), 7.29 (s, 2H), 3.29 (t, $J$ = 5.5 Hz, 4H), 2.76 (t, $J$ = 6.0 Hz, 4H), 2.03 – 1.89 (m, 4H). $^{13}$C NMR (101 MHz, $CDCl_3$) δ 190.1, 147.9, 129.4, 124.0, 120.3, 50.0, 27.7, 21.3.

**(*E*)-2-cyano-3-(1,2,3,5,6,7-hexahydropyrido[3,2,1-ij]quinolin-9-yl)acrylic acid (I-3[1])**: To a stirred solution of **I-2** (0.3 g, 1.491 mmol) in acetonitrile was added piperidine (0.221 mL, 2.23 mmol) and 2-cyanoacetic acid (0.190 g, 2.23 mmol), and the resulting mixture was heated to 60 °C and stirred for 5 hours. The reaction mixture was then cooled to RT and concentered under reduced pressure. the crude material was purified by column chromatography on silica gel using MeOH and DCM system to give the

desired compound **I-3** as a red solid. Yield: 0.250 g (62 %). $^1$H NMR (400 MHz, DMSO-d$_6$) δ 7.81 (s, 1H), 7.46 (s, 2H), 3.30 (t, *J* = 5.1 Hz, 4H), 2.66 (t, *J* = 5.6 Hz, 4H), 1.86 (d, *J* = 4.9 Hz, 4H).

**(*Z*)-tert-butyl (2-(2-cyano-3-(1,2,3,5,6,7-hexahydropyrido[3,2,1-*ij*]quinolin-9-yl)acrylamido) ethyl)carbamate (I-5)**: To a stirred solution of **I-3** (0.1 g, 0.372 mmol) in DMF/DCM (1:10, 5 mL) at 0 ºC, EDC.HCl (78 mg, 0.410 mmol) and HOBT (55 mg, 0.410 mmol) were added. The reaction mixture was stirred at 0 ºC for 1 hour and then tert-butyl (2-aminoethyl)carbamate (59 mg, 0.372 mmol) and DIPEA (114 mg, 1.118 mmol) were added. The reaction mixture was stirred at RT overnight. The reaction mixture was concentrated under reduced pressure and the obtained residue was purified using column chromatography on silica gel using ethyl acetate and hexane system to give compound **I-5** as a light yellow solid. Yield: 120 mg (78 %). $^1$H NMR (400 MHz, DMSO-d$_6$) δ 7.95 (s, 1H), 7.80 (s, 1H), 7.42 (s, 2H), 6.89 (s, 1H), 3.22 (d, *J* = 5.5 Hz, 2H), 3.05 (d, *J* = 5.3 Hz, 2H), 2.89 (s, 2H), 2.73 (s, 2H), 2.67 (s, 4H), 1.86 (s, 4H), 1.37 (s, 9H).

**(*Z*)-N-(2-aminoethyl)-2-cyano-3-(1,2,3,5,6,7-hexahydropyrido[3,2,1-*ij*]quinolin-9-yl) acrylamide (I-6):** To a stirred solution of **I-5** (0.1 g, 0.243 mmol) in DCM (5 mL), TFA (2.5 mL) was added at 0 ºC. The reaction mixture was stirred at RT for 5 hours, and then was concentrated under reduced pressure. The crude product was purified using column chromatography on silica gel using ethyl acetate and hexane system to give compound as a yellow solid. Yield: 60 mg (80 %) $^1$H NMR (400 MHz, DMSO-d$_6$) *δ* 8.09 (s, 1H), 7.85 (s, 1H), 7.73 (s, 2H), 7.45 (s, 2H), 3.43 (d, *J* = 5.7 Hz, 2H), 3.25 (s, 4H), 2.93 (s, 2H), 2.64 (d, *J* = 24.9 Hz, 4H), 1.87 (s, 4H). $^{13}$C NMR (101 MHz, DMSO-d$_6$) δ 163.8, 163.8, 151.2, 147.3, 130.8, 121, 119.2, 118.0, 94.4, 45.0, 38.0, 27.5, 21.1.ES-API: *m/z* 311.9 $(M+H)^+$.

**(*E*)-2-cyano-3-(1,2,3,5,6,7-hexahydropyrido[3,2,1-ij]quinolin-9-yl)-*N*-(2-(2-(8-imino-2,3,6,7-tetrahydrodicyclopenta[*b*,*e*]pyridin-4(1*H*,5*H*,8*H*)-yl)acetamido)ethyl)acrylamide (CCVJ-Cmpd 1)**: To a stirred solution of **I-6** in DMF (5 mL) at 0 ºC, EDC.HCl (45.3 mg, 0.236 mmol) and HOBT (32 mg, 0.236 mmol) were added. The reaction mixture stirred at 0 ºC for 1 hour and then **I-7** (96.4 mg, 0.215

mmol) and DIPEA (83 mg, 0.645 mmol) were added. The reaction mixture was stirred at RT overnight. The reaction mixture was concentrated under reduced pressure and the crude product was purified using column chromatography on silica gel using methanol and dichloromethane system to give the title compound as a red solid. Yield: 60 mg (53 %). $^{1}$H NMR (400 MHz, DMSO-$d_6$) $\delta$ 8.58 (s, 1H), 8.03 (s, 1H), 7.81 (s, 1H), 7.63 (s, 2H), 7.40 (s, 1H), 4.74 (s, 2H), 3.31 – 3.23 (m, 8H), 2.97 (s, 4H), 2.72 (d, *J* = 7.3 Hz, 4H), 2.65 (t, *J* = 15.4 Hz, 4H), 2.17 – 2.05 (m, 4H), 1.87 (s, 4H); ES-API: *m/z* 524.8 $(M+H)^+$.

**The synthesis of biotin-Cmpd 1**

**tert-butyl (1-(8-imino-2,3,6,7-tetrahydrodicyclopenta[b,e]pyridin-4(1*H*,5*H*,8*H*)-yl)-2-oxo-6,9,12-trioxa-3-azatetradecan-14-yl)carbamate (I-9)**: To a stirred solution of **I-7** (250 mg, 1.07 mmol) in DMF (10 mL) at 0 °C, EDC.HCl (226 mg, 1.177 mmol) and HOBT (159 mg, 1.177 mmol) were added. The reaction mixture was stirred at 0 °C for 1 hour and then a solution of **I-8** (315 mg, 1.07 mmol) and DIPEA (0.417 g, 0.321 mmol) were added. The reaction mixture was stirred at RT for 16 hours. Then the reaction mixture was concentrated under reduced pressure and the crude product was purified using column chromatography on silica gel using methanol and dichloromethane system to give the title compound as a red solid. Yield: 500 mg (91 %). $^{1}$H NMR (400 MHz, DMSO-$d_6$) δ 8.59 (s, 1H), 7.66 (s, 1H), 6.77 (s, 1H), 4.79 (s, 2H), 3.62 – 3.42 (m, 12H), 3.30 – 3.22 (m, 3H), 3.05 (d, *J* = 5.6 Hz, 2H), 2.97 (t, *J* = 7.2 Hz, 4H), 2.76 (t, *J* = 7.0 Hz, 4H), 2.20 – 2.09 (m, 4H), 1.36 (s, 9H); $^{13}$C NMR (101 MHz, DMSO-$d_6$) δ 165.5, 156.4, 156.1, 152.5, 121.4, 78.1, 70.2, 70.2, 70.1, 70.0, 70.0, 69.6, 69.2, 67.1, 54.1, 39.0, 31.4, 28.7, 28.5, 22.1; ES-API: *m/z* 506.8 $(M+H)^+$.

***N*-(2-(2-(2-(2-aminoethoxy)ethoxy)ethoxy)ethyl)-2-(8-imino-2,3,6,7-tetrahydrodicyclopenta [*b*,*e*]pyridin-4(1*H*,5*H*,8*H*)-yl)acetamide (I-10)**: To a stirred solution of **I-9** (450 mg, 0.889 mmol) in DCM (2 mL) was added TFA (0.304 mg, 2.666 mmol) at 0 °C. The resulting mixture was warmed to RT and was stirred for another 16 hours. The reaction mixture was concentrated and the obtained residue was purified on silica gel (230-400 mesh) to give the desired compound as yellow gummy liquid. Yield:

300 mg (83 %). $^{1}$H NMR (400 MHz, DMSO-$d_6$) δ 8.60 (s, 1H), 7.87 (s, 2H), 7.66 (s, 1H), 4.79 (s, 2H), 3.54 (d, *J* = 8.3 Hz, 6H), 3.52 (s, 4H), 3.45 (t, *J* = 5.3 Hz, 2H), 3.27 (d, *J* = 5.3 Hz, 2H), 2.96 (t, *J* = 5.9 Hz, 7H), 2.76 (t, *J* = 7.0 Hz, 4H), 2.21 – 2.07 (m, 4H).

***N*-(1-(8-imino-2,3,6,7-tetrahydrodicyclopenta[b,e]pyridin-4(1*H*,5*H*,8*H*)-yl)-2-oxo-6,9,12-trioxa-3-azatetradecan-14-yl)-5-((3*aS*,4*S*,6*aR*)-2-oxohexahydro-1*H*-thieno[3,4-d]imidazol-4-yl)pentanamide (biotin-Cmpd 1):** To a stirred solution of biotin (50 mg, 0.184 mmol) in DMF (5 mL) at 0 °C, EDC.HCl (54 mg, 0.284 mmol) and HOBT (27 mg, 0.203 mmol) were added. The reaction mixture was stirred at 0 °C for 1 hour and then **I-10** (75 mg, 0.184 mmol) and DIPEA (0.143 ml, 0.554 mmol) were added. The reaction mixture was stirred at RT for 16 hours, and was concentrated under reduced pressure. The obtained residue was purified using column chromatography on alumina using methanol and dichloromethane as eluent to give the title compound as a yellow gummy compound. Yield: 40 mg (51 %). $^{1}$H NMR (400 MHz, DMSO-$d_6$) δ 8.63 (s, 1H), 8.29 (s, 2H), 7.84 (s, 1H), 7.65 (s, 1H), 6.36 (d, *J* = 18.4 Hz, 2H), 4.77 (s, 2H), 4.29 – 4.23 (m, 1H), 4.08 (s, 1H), 3.47 (d, *J* = 3.7 Hz, 7H), 3.41 (t, *J* = 5.3 Hz, 2H), 3.35 (t, *J* = 5.8 Hz, 2H), 3.23 (d, *J* = 5.2 Hz, 2H), 3.18 – 3.10 (m, 2H), 3.05 (s, 1H), 2.93 (t, *J* = 7.2 Hz, 4H), 2.77 – 2.67 (m, 4H), 2.53 (d, *J* = 12.5 Hz, 1H), 2.20 – 2.06 (m, 4H), 2.02 (t, *J* = 7.1 Hz, 2H), 1.62 – 1.51 (m, 1H), 1.50 – 1.34 (m, 3H), 1.32 – 1.17 (m, 2H). ES-API: *m/z* 632.5 $(M+H)^+$.

**The synthesis of intermediate I-7**

**8-amino-4-(2-(tert-butoxy)-2-oxoethyl)-1,2,3,5,6,7-hexahydrodicyclopenta[b,e]pyridin-4-ium (I-14):** **I-12** (1.0 g, 9.25 mmol), **I-13** (0.77 g, 9.253 mmol), and 1.383 g of anhydrous $ZnCl_2$ in 30 mL of dry xylene were boiled with a reflux condenser for 5 hours. Then the reaction mixture was cooled to RT, and the solvent was decanted. The gummy residue was ground in the 30 mL of 5 N KOH, and was filtered. The precipitate was washed with water, and then was suspended in hot $CHCl_3$, and filtered. The filtrate was concentrated, and the obtained residue was purified on silica gel (230-400 mesh) using MeOH/DCM as a mobile phase. The major fraction

was concentrated, and the substance was crystallized from aqueous alcohol to give the intermediate as a white solid. Yield: 1.2 g (74 %).

To a stirred suspension of the intermediate (1.0 g, 5.74 mmol) in acetone was added tert-butyl 2-bromoacetate (1.67 g, 8.62 mmol) at RT. The reaction mixture was stirred at RT for 1 hour. The reaction mixture was filtered and the precipitate was washed with cold acetone to give the title compound as a white solid. Yield: 1.4 g (84 %). $^1$H NMR (400 MHz, DMSO-$d_6$) δ 7.76 (s, 2H), 5.01 (s, 2H), 2.98 (t, $J$ = 7.0 Hz, 4H), 2.77 (t, $J$ = 6.9 Hz, 4H), 2.26 – 2.10 (m, 4H), 1.45 (s, 9H).

**tert-butyl 2-(8-imino-2,3,6,7-tetrahydrodicyclopenta[*b*,*e*]pyridin-4(1*H*,5*H*,8*H*)-yl)acetate (I-15)**: **I-14** (1.3 g, 4.49 mmol) and potassium tertbutoxide (0.504 g, 4.49 mmol) were dissolved in THF (75 mL). The reaction mixture was stirred at RT for 1.5 hours. The solvent was removed in vacuo, and the residue was extracted with toluene (500 mL). After filtration, the solvent was removed in vacuo to afford **I-15** as a white solid. Yield: 1.1 g (85 %). $^1$H NMR (400 MHz, DMSO-$d_6$) δ 7.76 (s, 1H), 5.00 (s, 2H), 2.97 (s, 4H), 2.77 (s, 4H), 2.16 (s, 4H), 1.44 (s, 9H).

**2-(8-imino-2,3,6,7-tetrahydrodicyclopenta[b,e]pyridin-4(1*H*,5*H*,8*H*)-yl)acetic acid (I-7):** To a stirred solution of **I-15** (1.0 g, 3.47 mmol) in DCM (10 mL), TFA (1.18 g, 10.41 mmol) was added at 0 °C. The reaction mixture was stirred at RT for 16 hours, and then was concentrated under reduced pressure. The obtained solid was washed with diethyl ether and was used for next step without further purification. Yield: 700 mg (86 %). $^1$H NMR (400 MHz, DMSO-$d_6$) δ 8.31 (s, 1H), 7.69 (s, 1H), 4.97 (s, 2H), 3.00 (s, 4H), 2.77 (s, 4H), 2.15 (s, 4H).

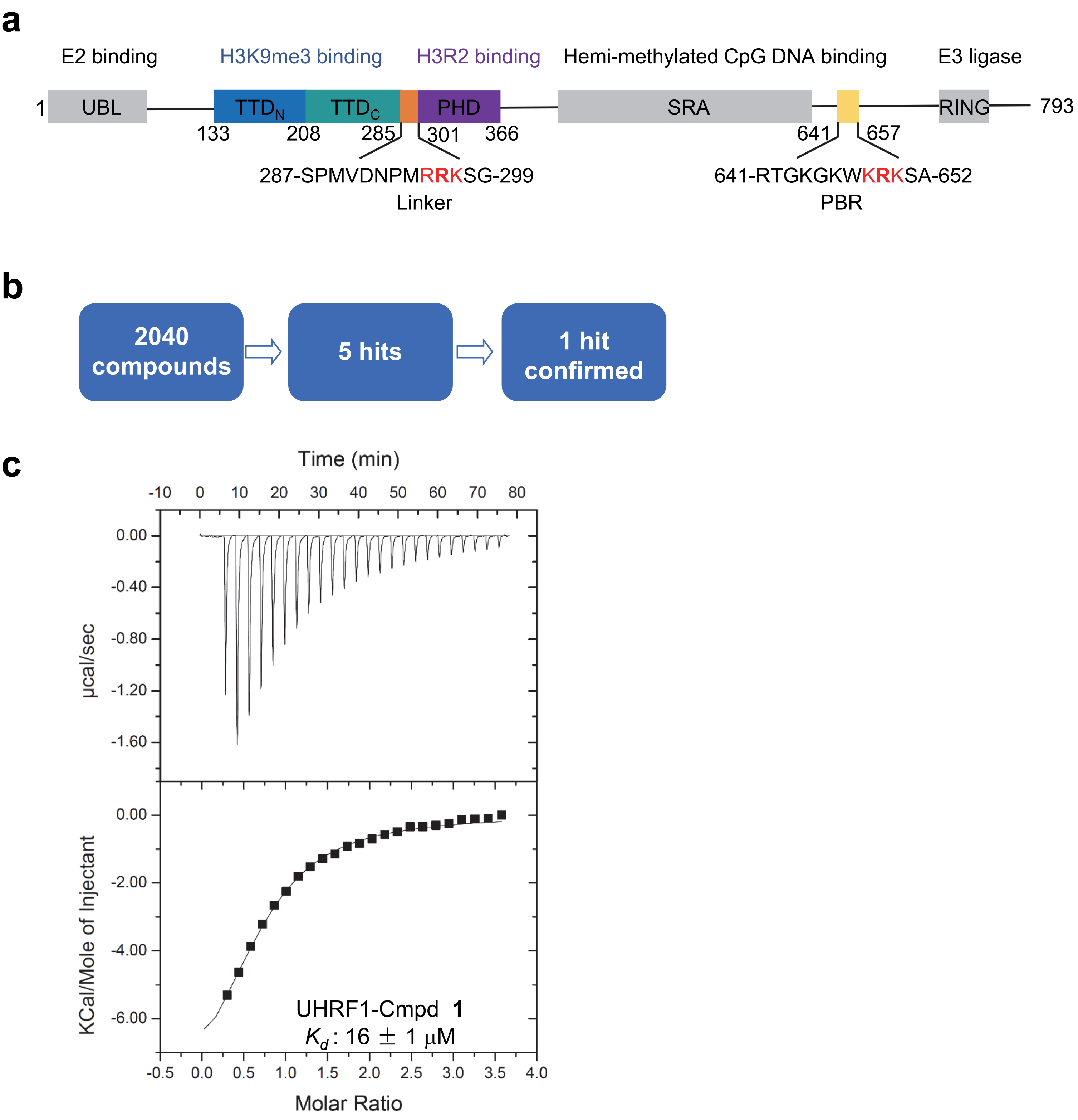


**Supplementary Fig. 1** Fragment screening for tandem Tudor domain (TTD) of UHRF1. **a** Domain structure of UHRF1. UBL, ubiquitin-like domain; TTD, tandem Tudor domain, containing $TTD_N$ and $TTD_C$ sub-domains; PHD, plant homeodomain; SRA, SET and RING associated domain; PBR, polybasic region; RING, really interesting new gene. **b** Flow-chart of fragment screening for TTD of UHRF1. **c** ITC binding curve for the titration of compound **1** to TTD of UHRF1. ITC data shown are representative of two independent experiments.

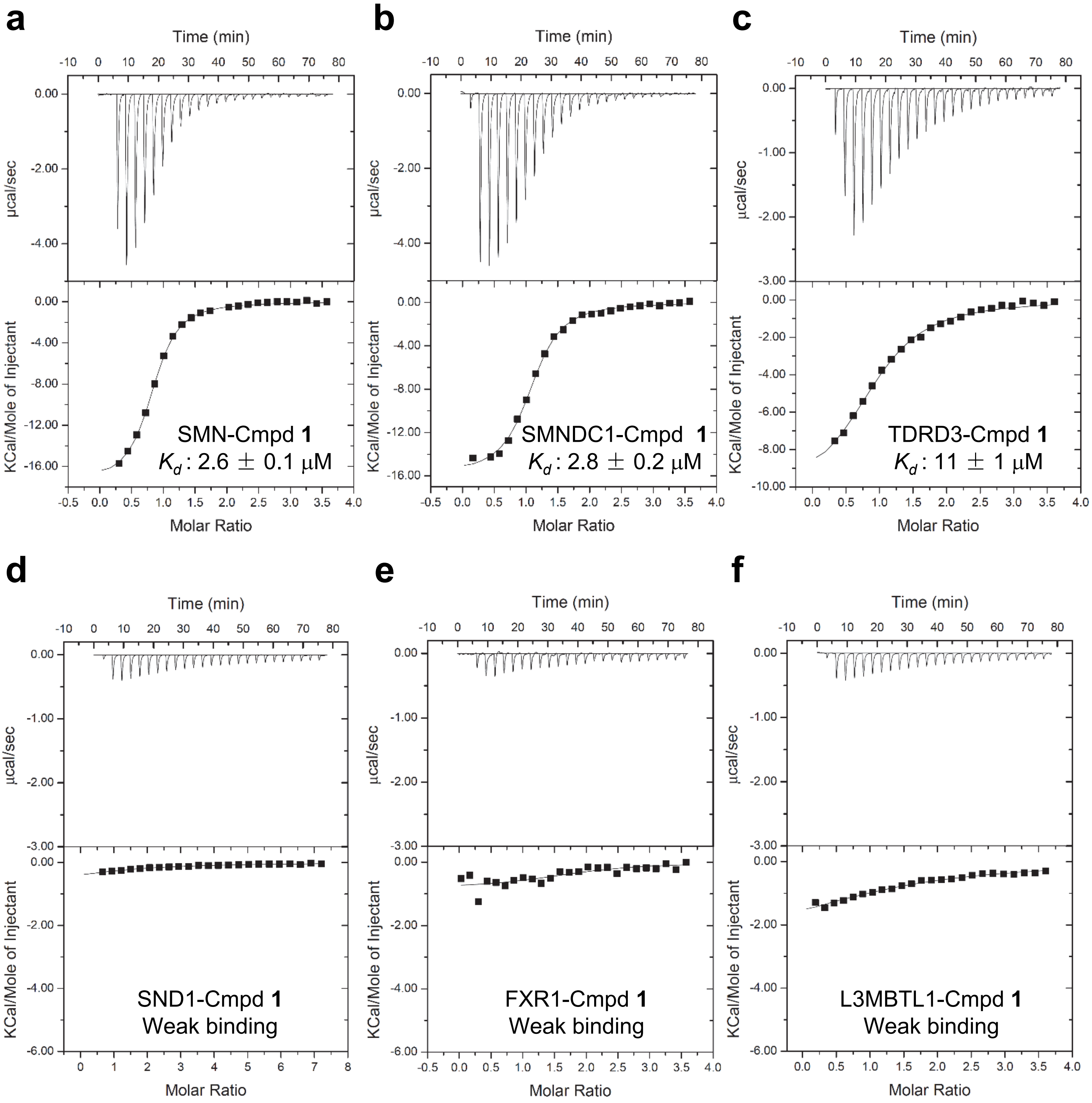


**Supplementary Fig. 2** Compound **1** prefers to binding to Tudor domain of SMN. ITC binding curves for the titration of compound **1** to the Tudor domain of **a** SMN, **b** SMNDC1, **c** TDRD3, **d** SND1, **e** FXR1, and **f** MBT repeats of L3MBTL1, respectively. ITC data shown are representative of two independent experiments.

**a**

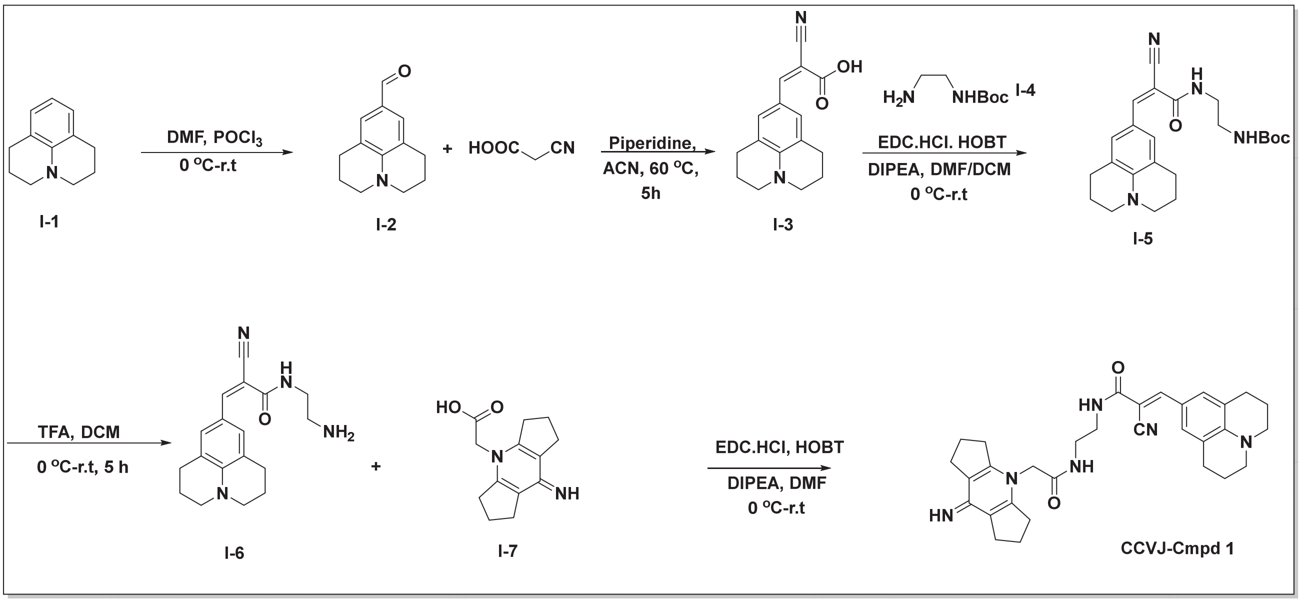


## A scheme outlining synthesis of CCVJ-Cmpd 1

**b**

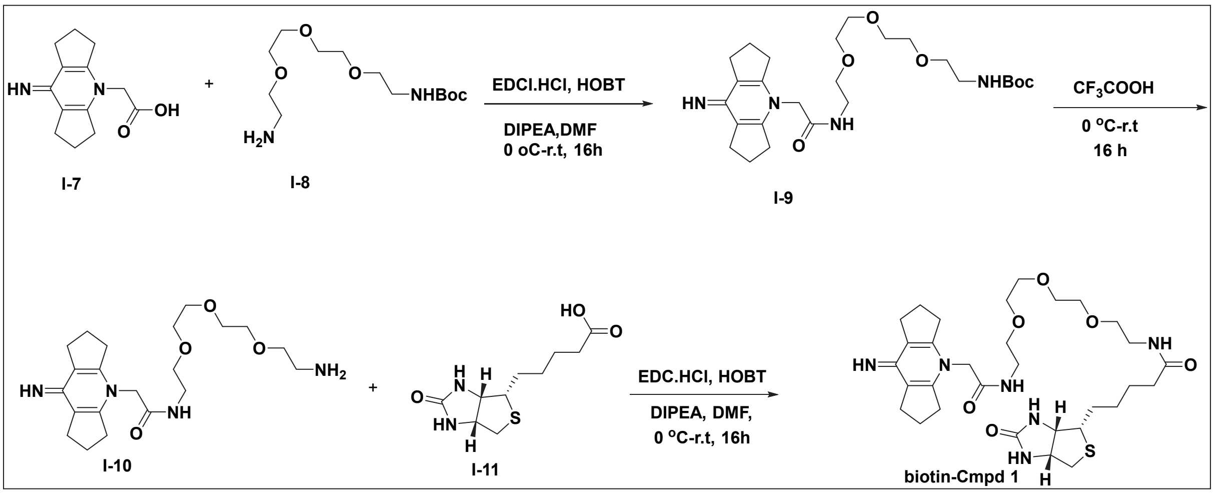


## A scheme outlining synthesis of biotin-Cmpd 1

**c**

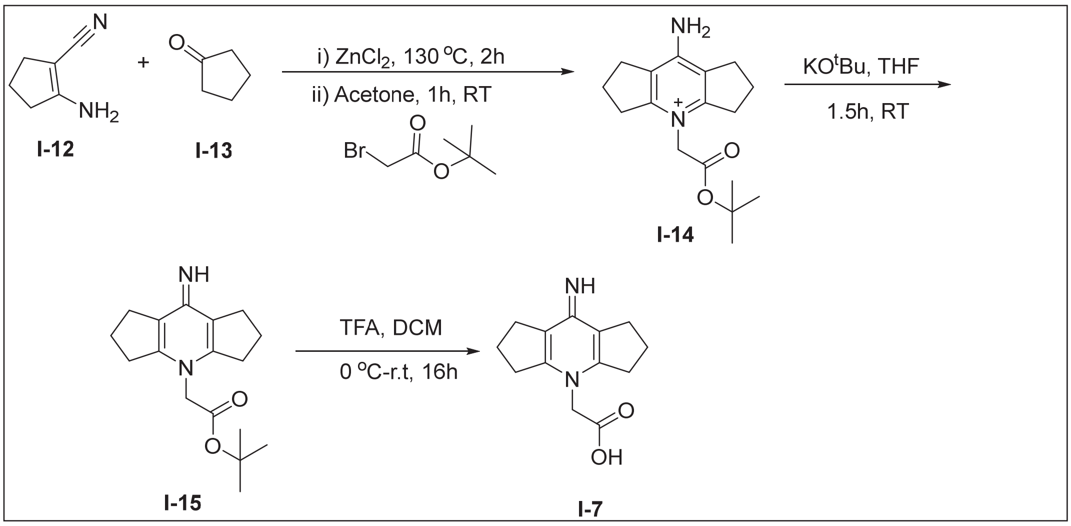


## A scheme outlining synthesis of intermediate I-7

**Supplementary Fig. 3** Synthesis and characterization of CCVJ-Cmpd **1**, biotin-Cmpd **1** and associated intermediate compounds. (To be continued)

d

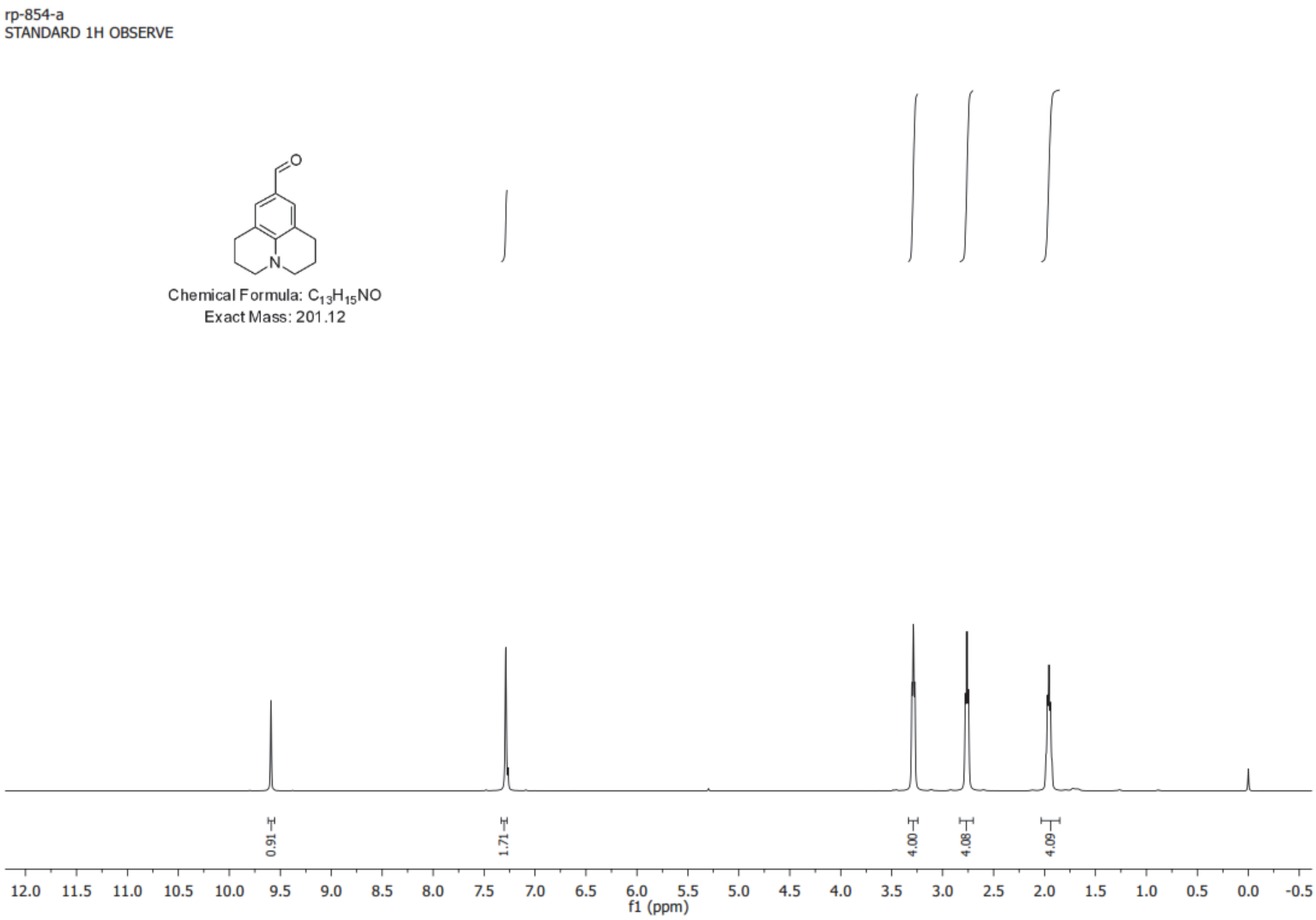


**$^1$H NMR spectrum of compound I-2**

e

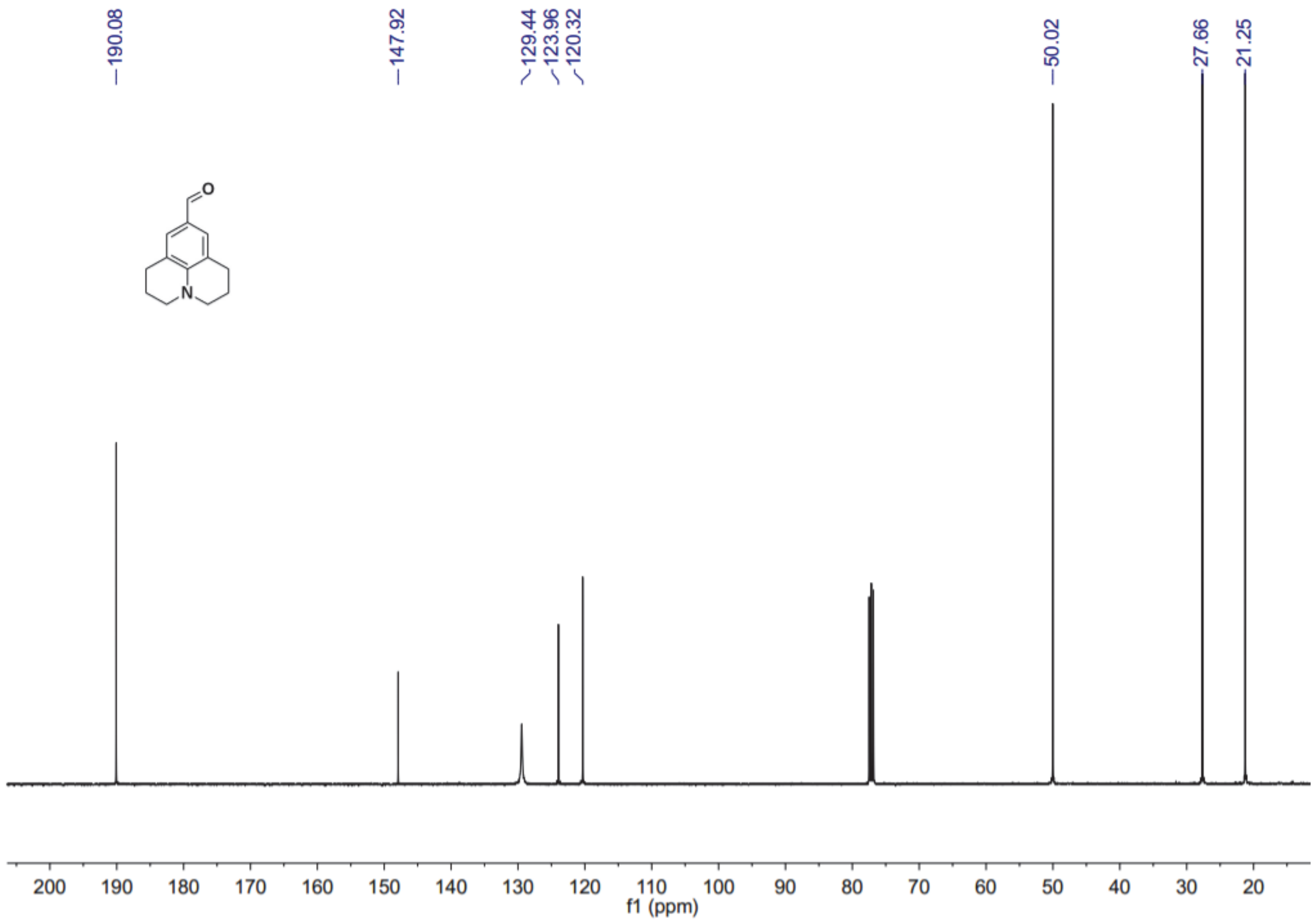


**$^{13}$C NMR spectrum of compound I-2**

**Supplementary Fig. 3** Synthesis and characterization of CCVJ-Cmpd **1**, biotin-Cmpd **1** and associated intermediate compounds. (To be continued)

**f**

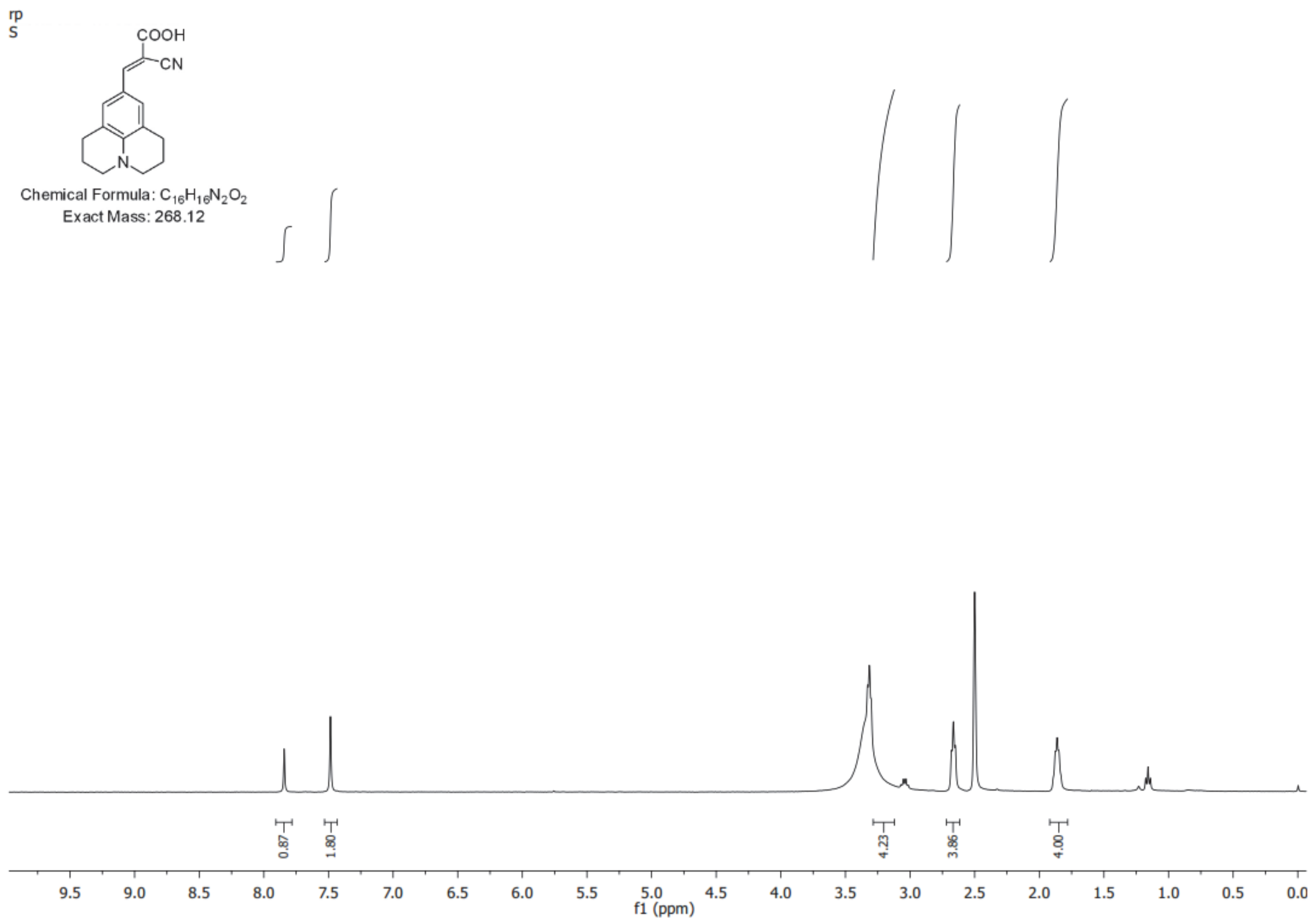


$^1$H NMR spectrum of compound I-3

**g**

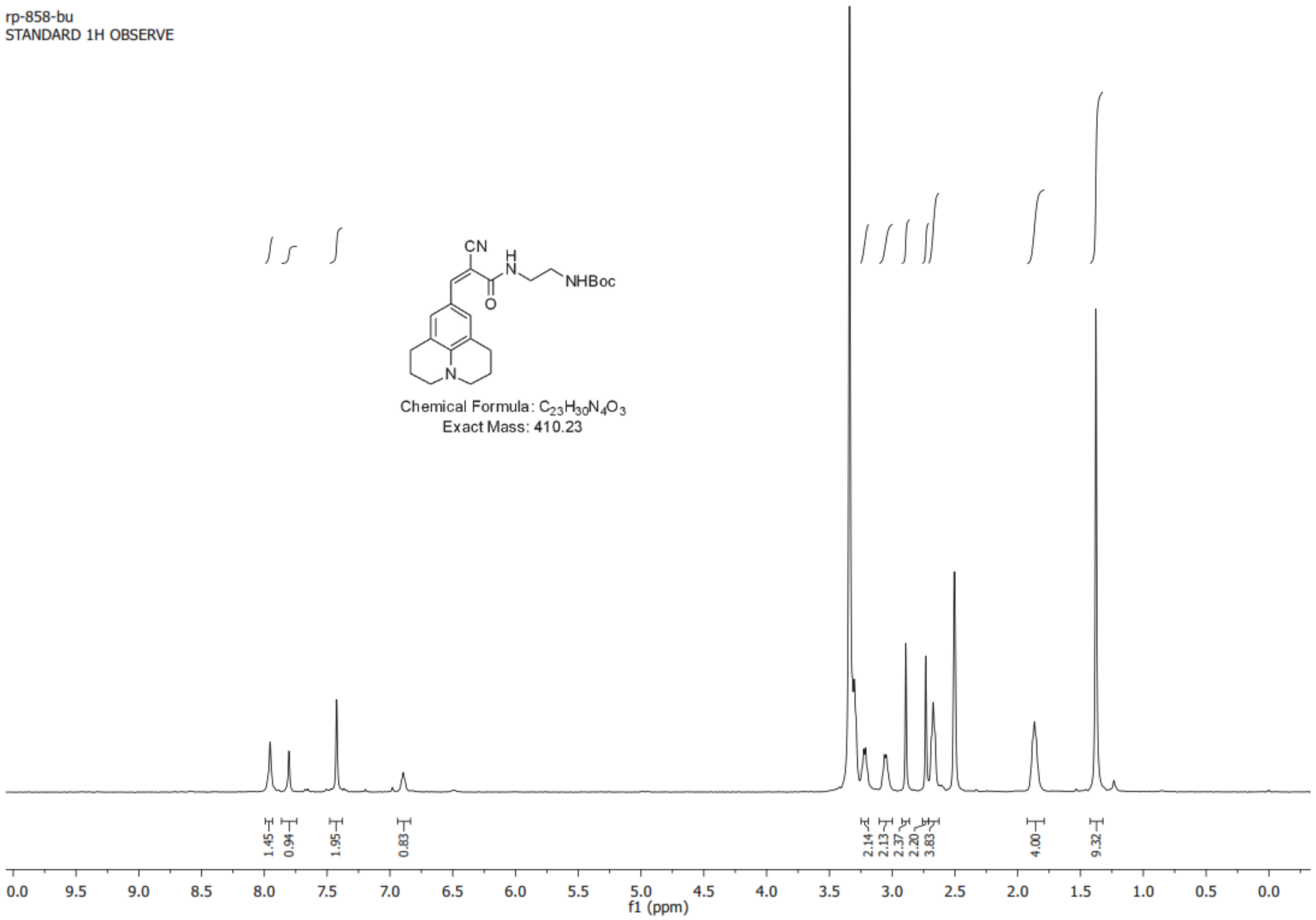


$^1$H NMR spectrum of compound I-5

**Supplementary Fig. 3** Synthesis and characterization of CCVJ-Cmpd **1**, biotin-Cmpd **1** and associated intermediate compounds. (To be continued)

**h**

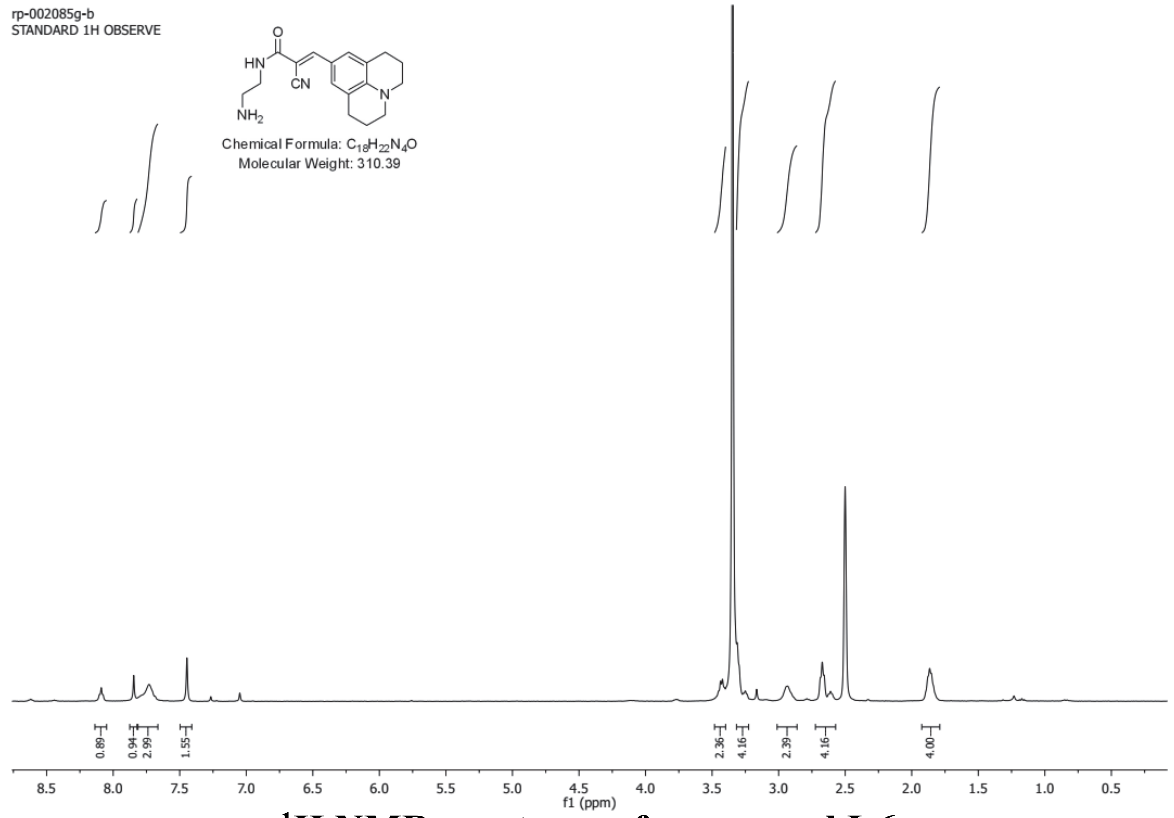


$^1$H NMR spectrum of compound I-6

**i**

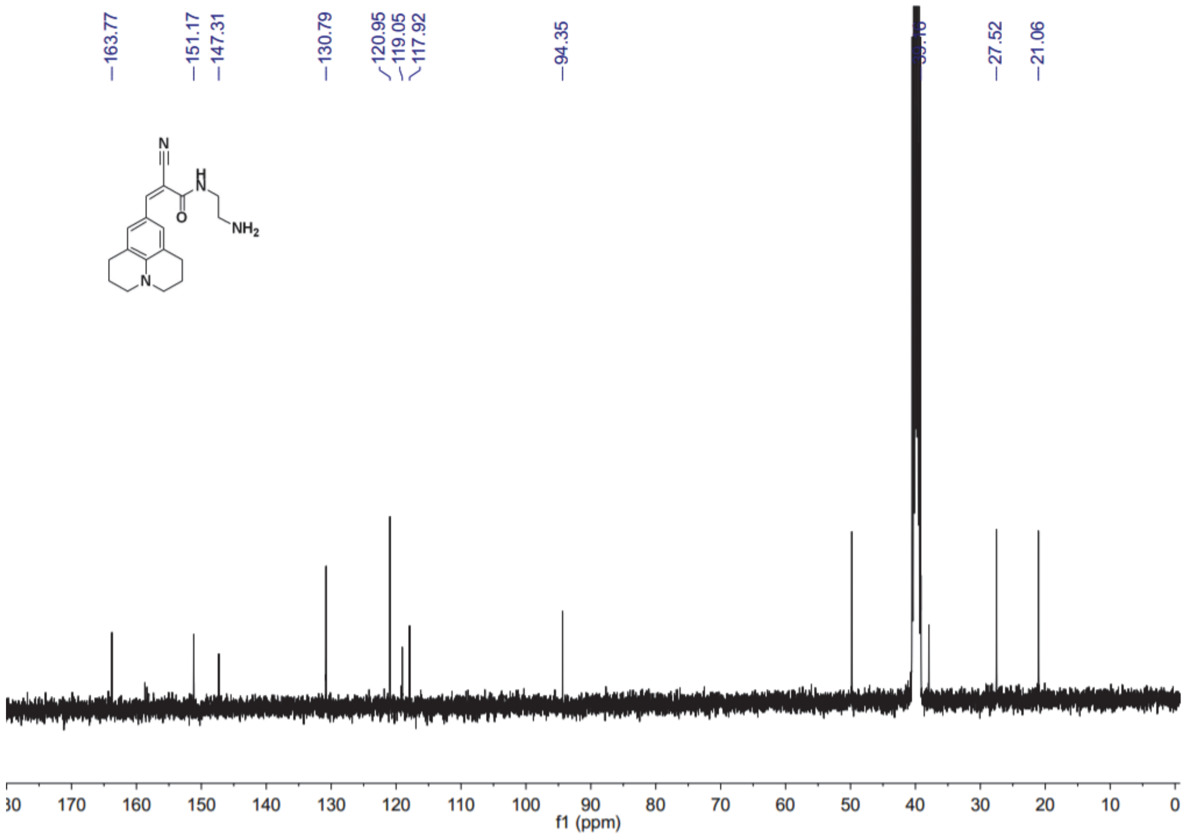


$^{13}$C NMR spectrum of compound I-6

**j**

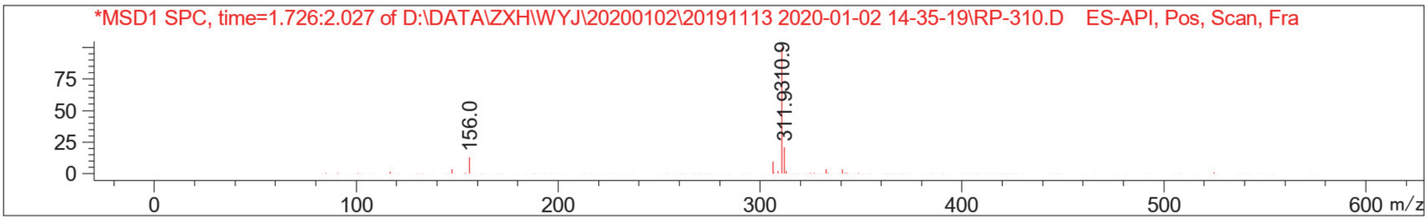


Mass spectrum of compound I-6

**Supplementary Fig. 3** Synthesis and characterization of CCVJ-Cmpd **1**, biotin-Cmpd **1** and associated intermediate compounds. (To be continued)

**k**

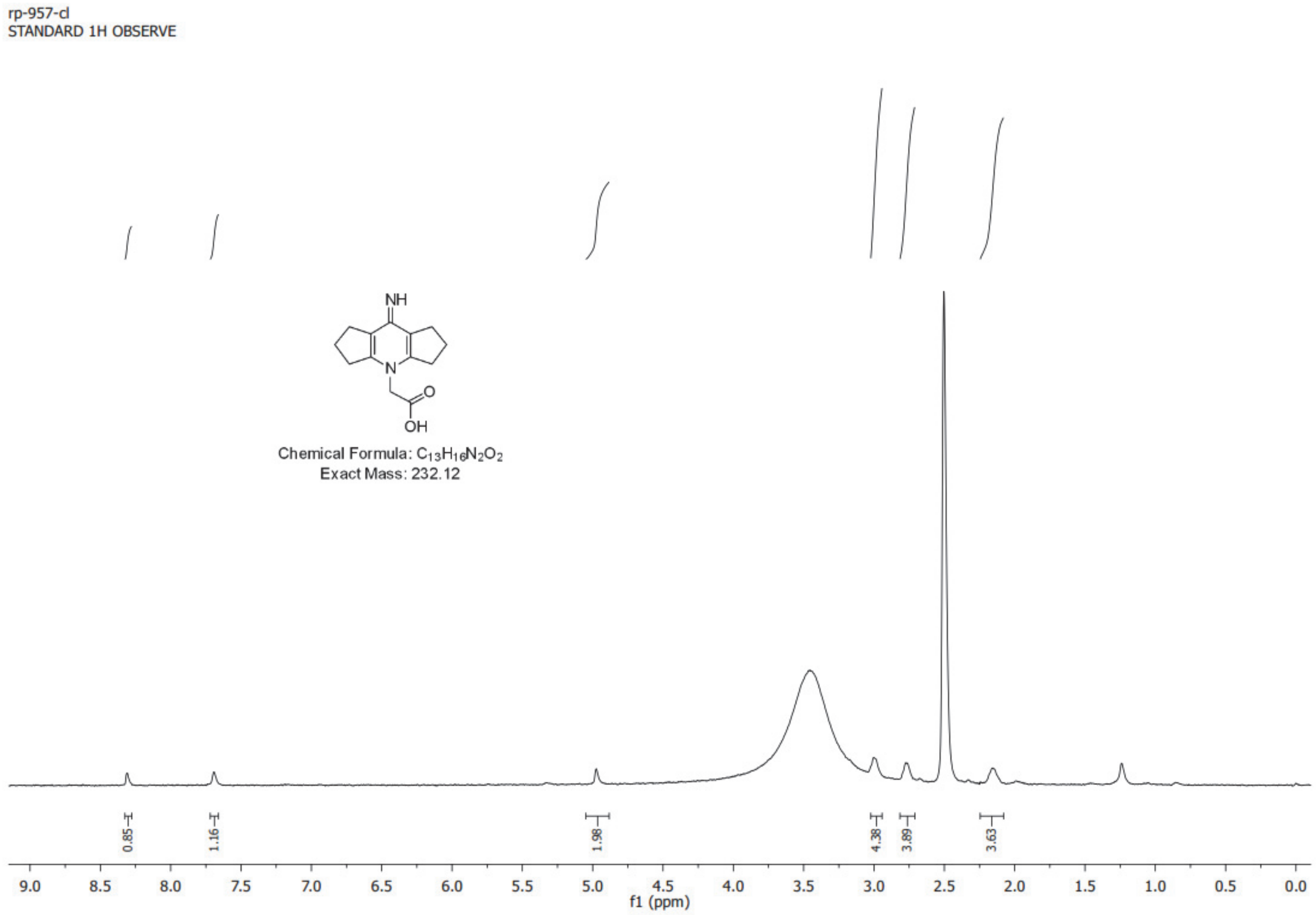


**$^1$H NMR spectrum of compound I-7**

**l**

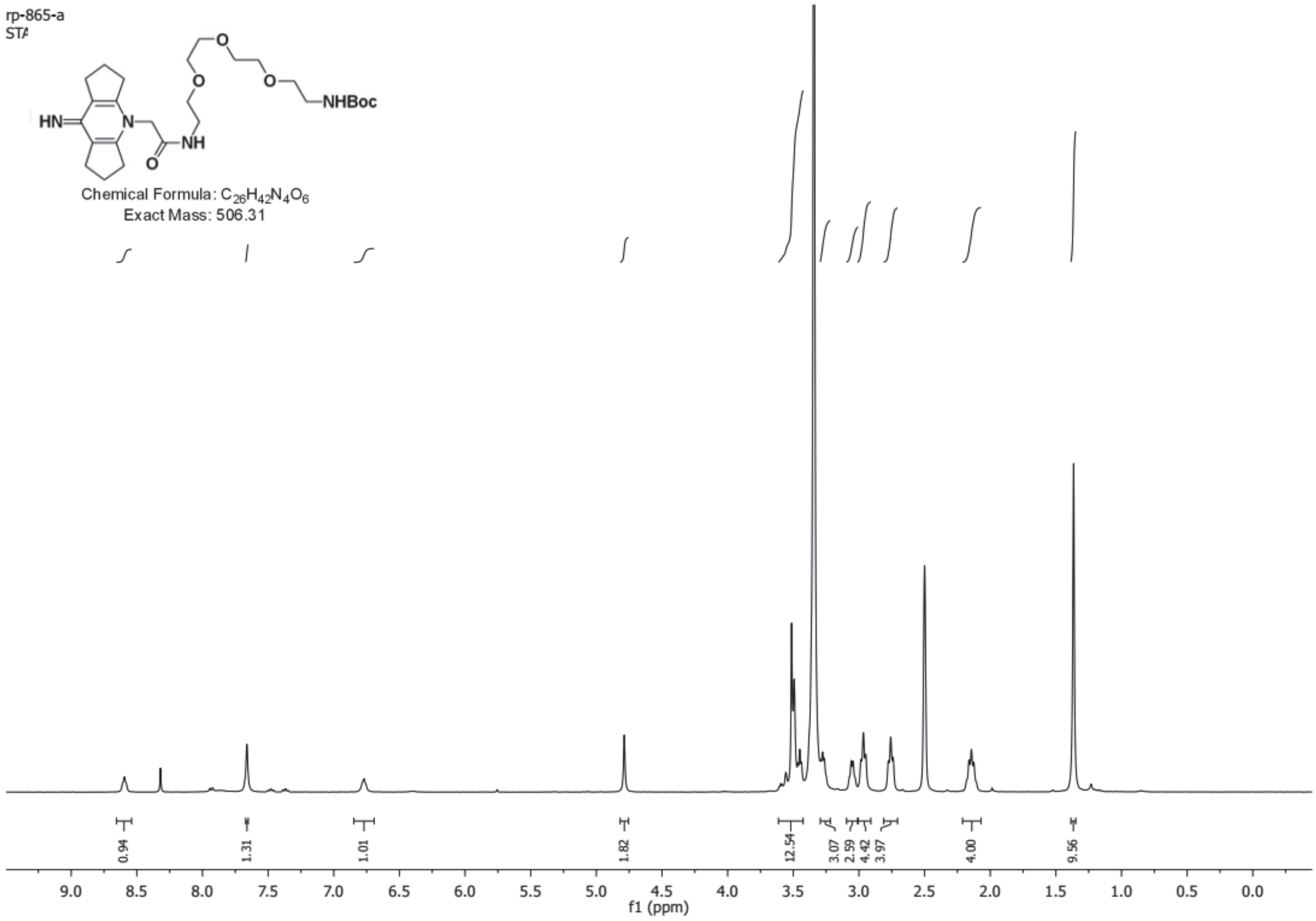


**$^1$H NMR spectrum of compound I-9**

**Supplementary Fig. 3** Synthesis and characterization of CCVJ-Cmpd **1**, biotin-Cmpd **1** and associated intermediate compounds. (To be continued)

**m**

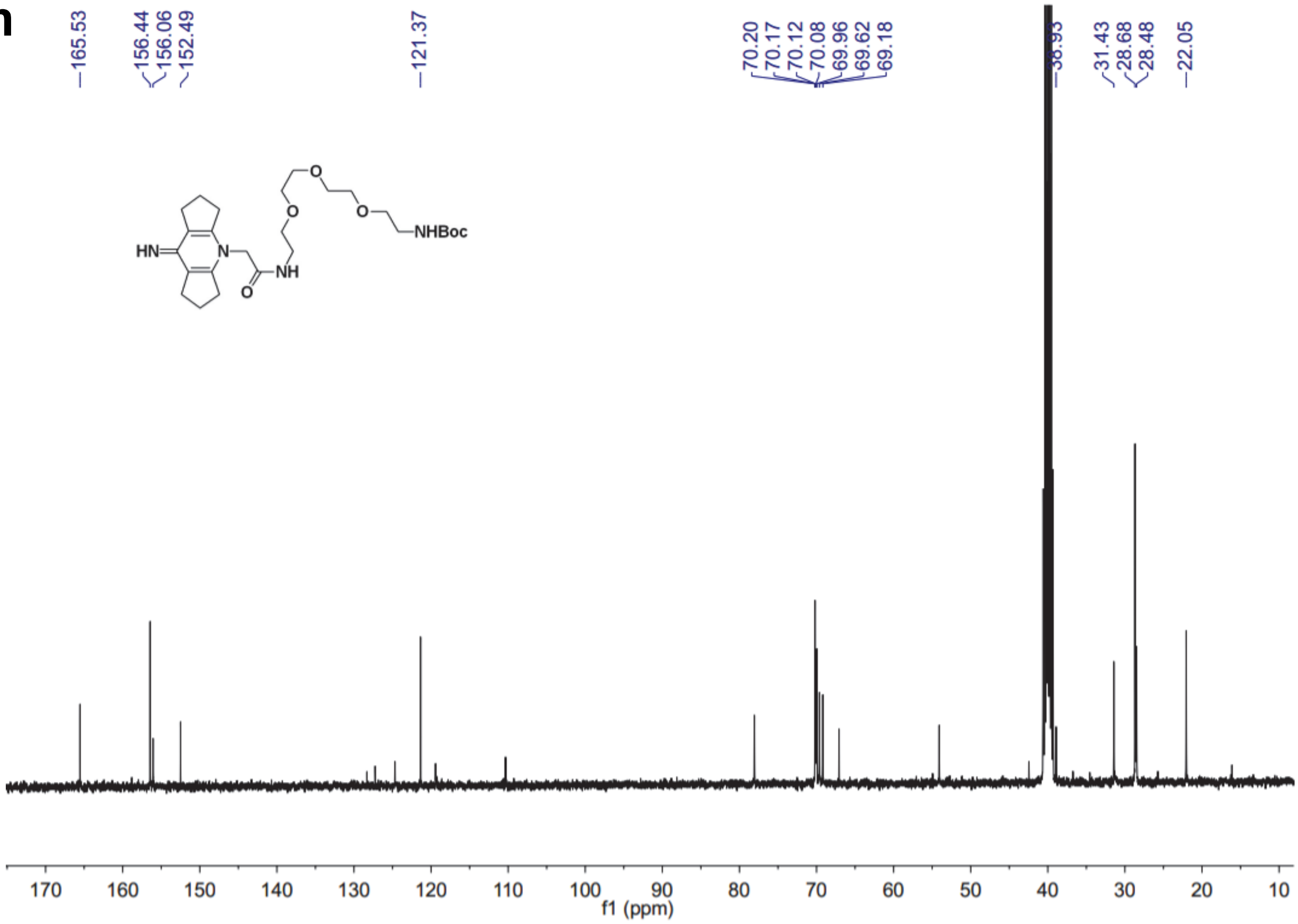


**$^{13}$C NMR spectrum of compound I-9**

**n**

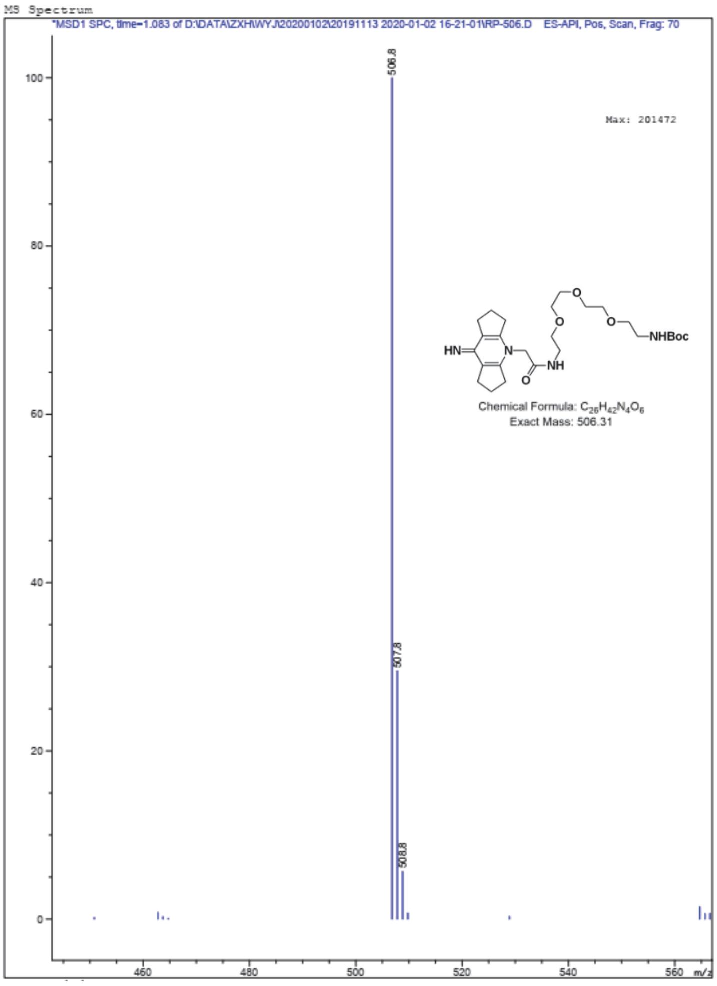


**Mass spectrum of compound I-9**

**Supplementary Fig. 3** Synthesis and characterization of CCVJ-Cmpd **1**, biotin-Cmpd **1** and associated intermediate compounds. (To be continued)

**o**

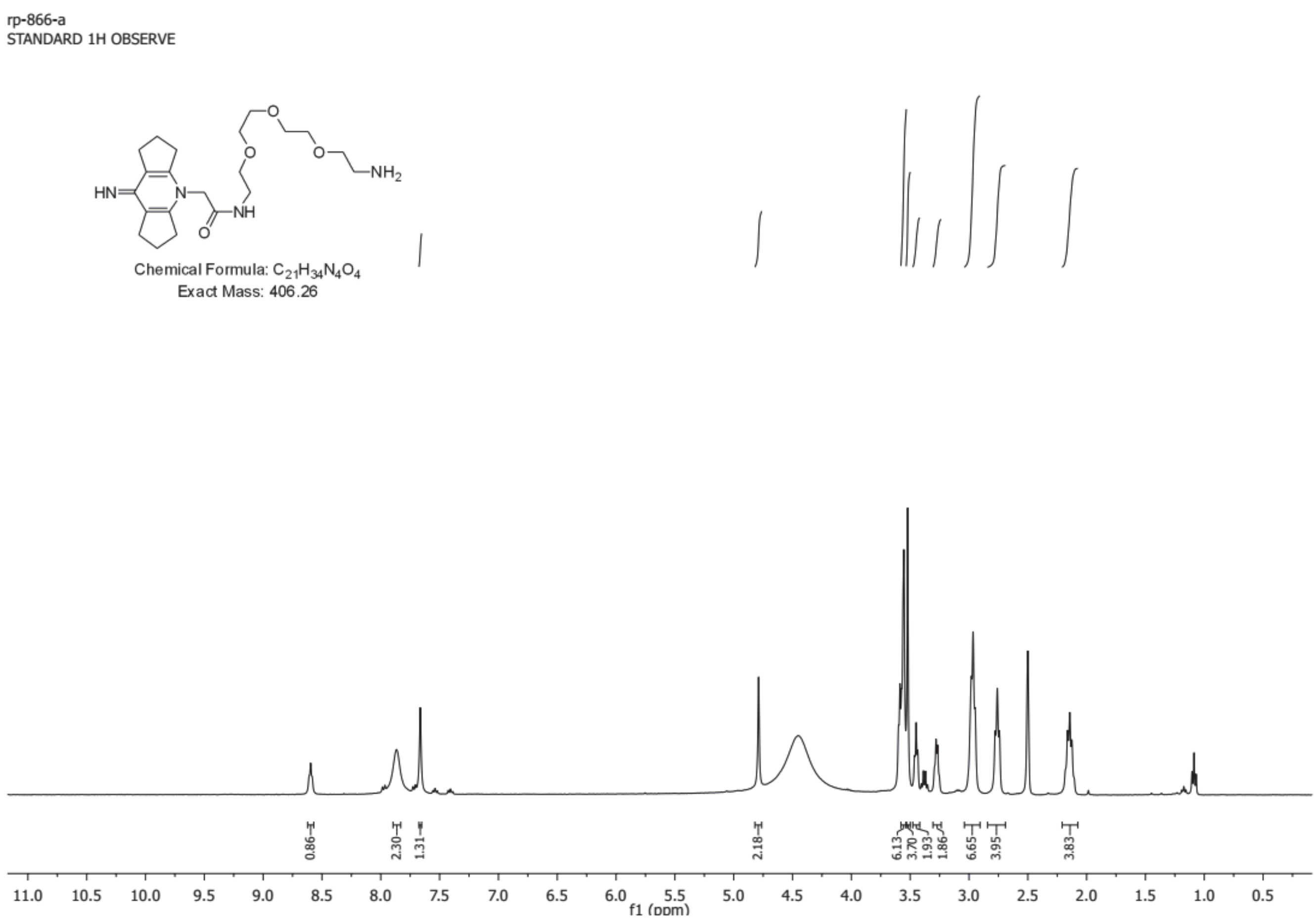


**$^1$H NMR spectrum of compound I-10**

**p**

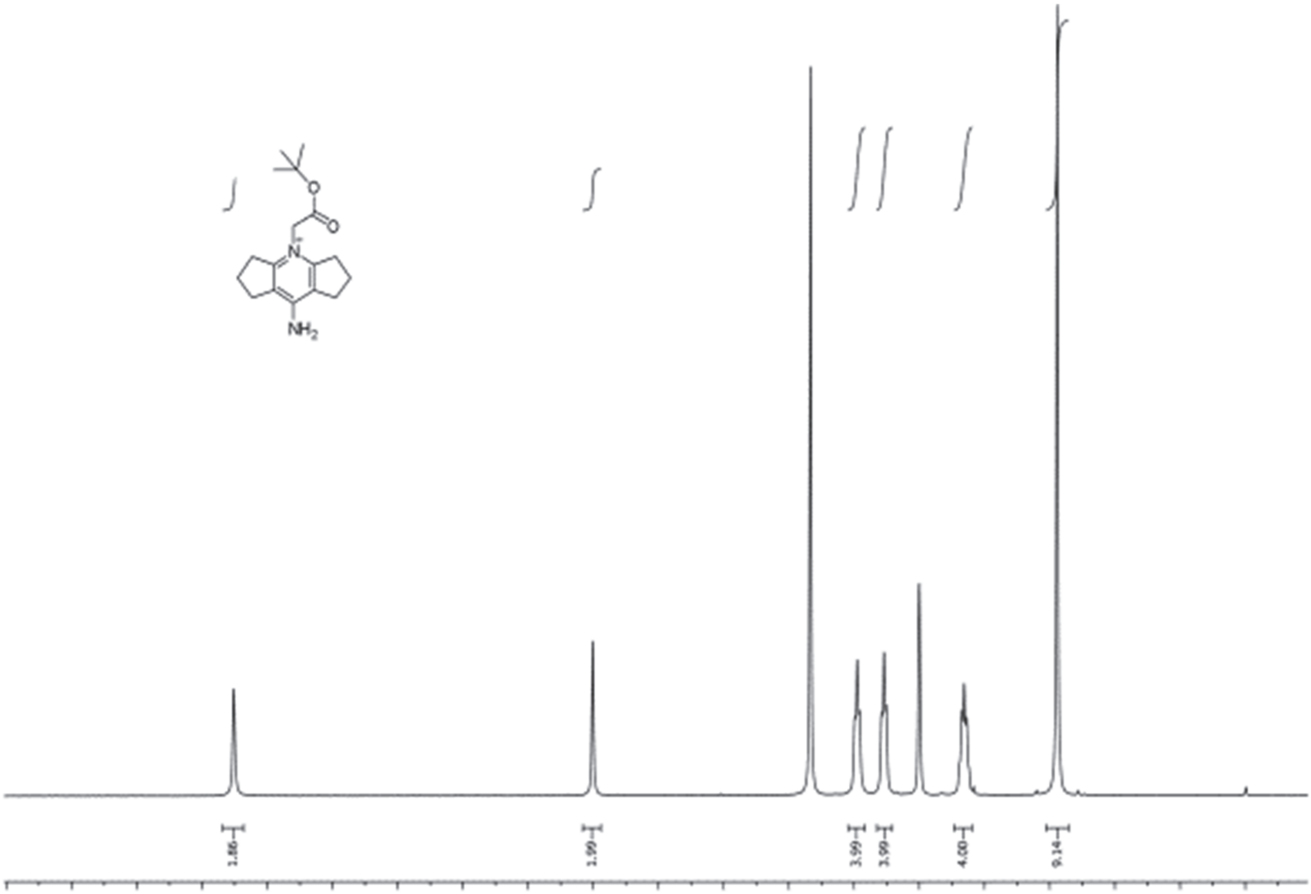


**$^1$H NMR spectrum of compound I-14**

**Supplementary Fig. 3** Synthesis and characterization of CCVJ-Cmpd **1**, biotin-Cmpd **1** and associated intermediate compounds. (To be continued)

**q**

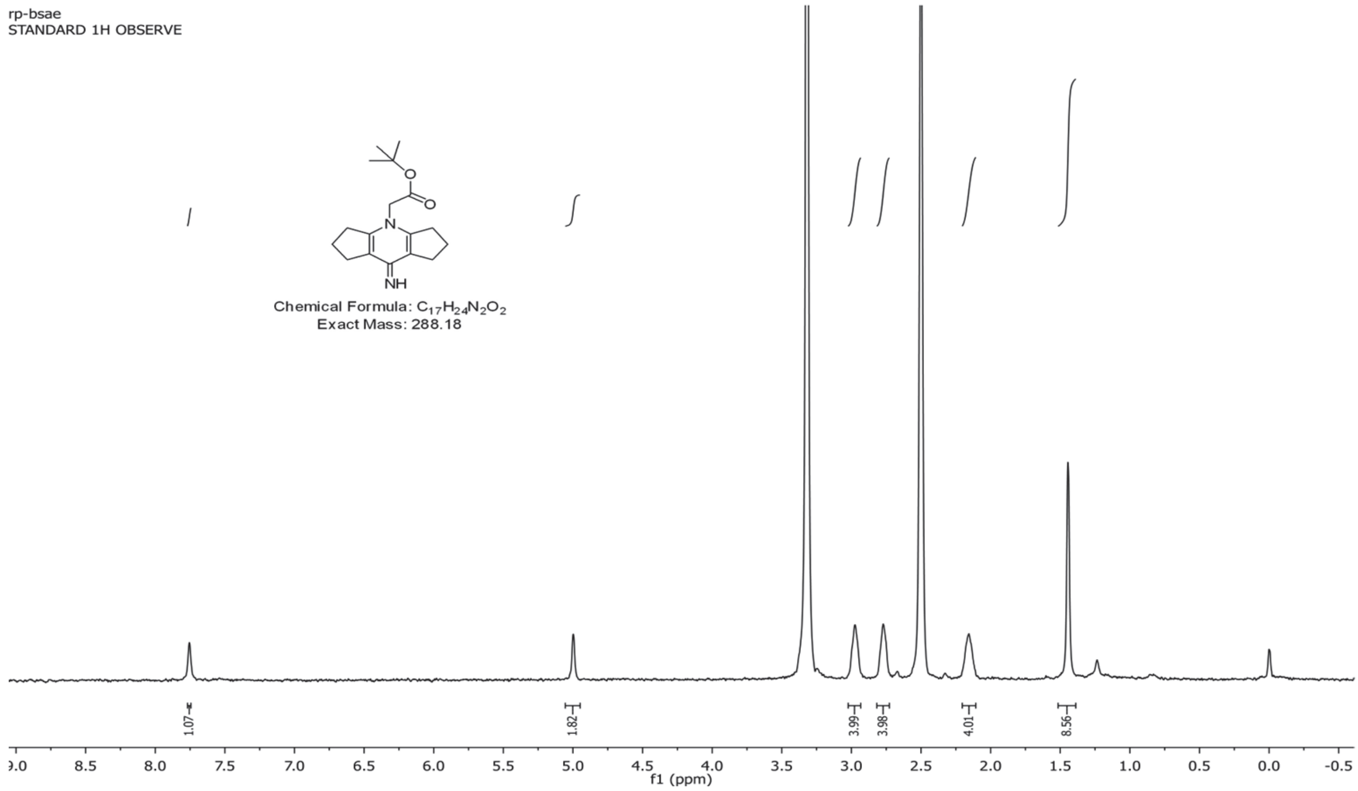


**$^1$H NMR spectrum of compound I-15**

**r**

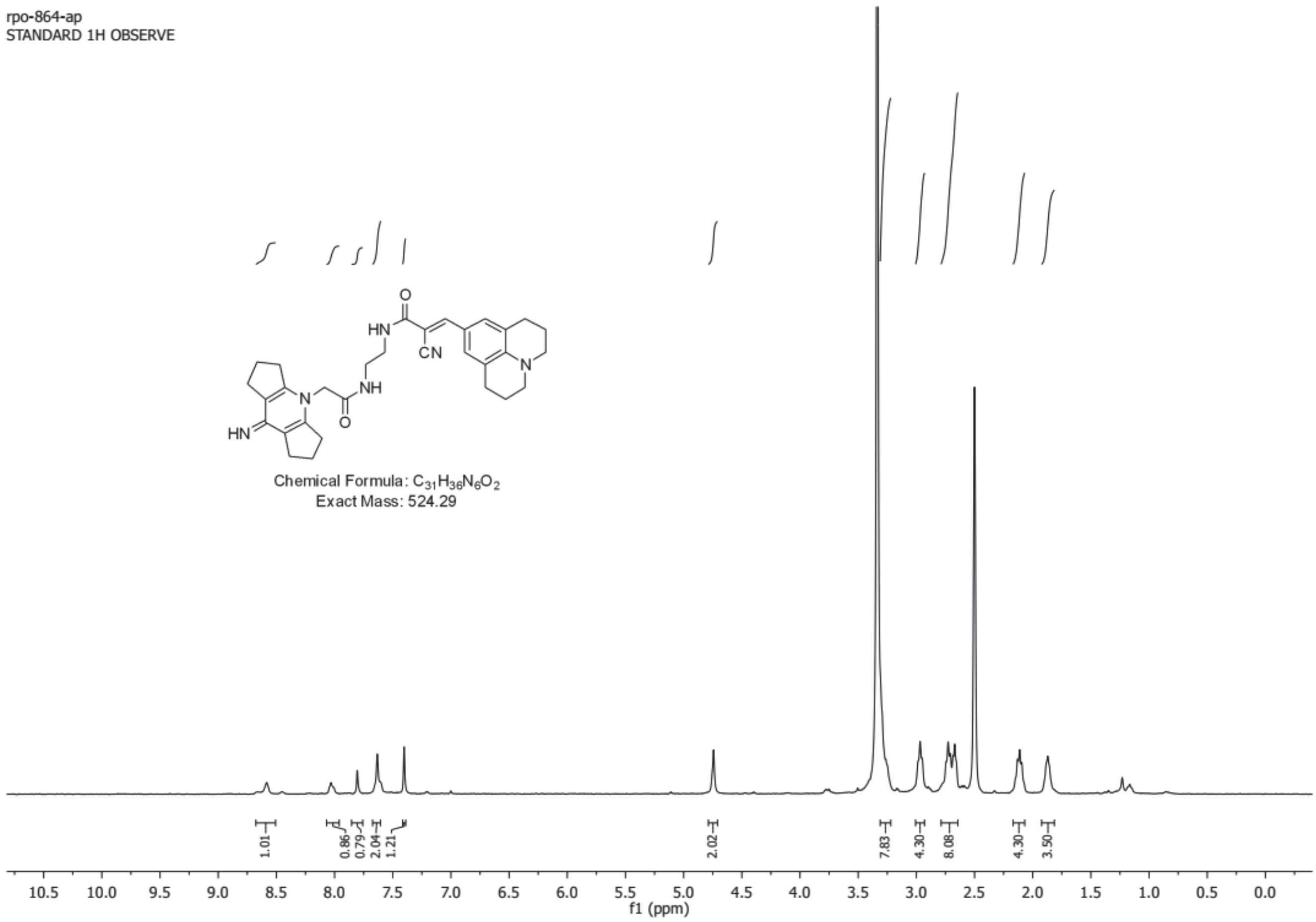


**$^1$H NMR spectrum of compound CCVJ-Cmpd 1**

**Supplementary Fig. 3** Synthesis and characterization of CCVJ-Cmpd **1**, biotin-Cmpd **1** and associated intermediate compounds. (To be continued)

**s**

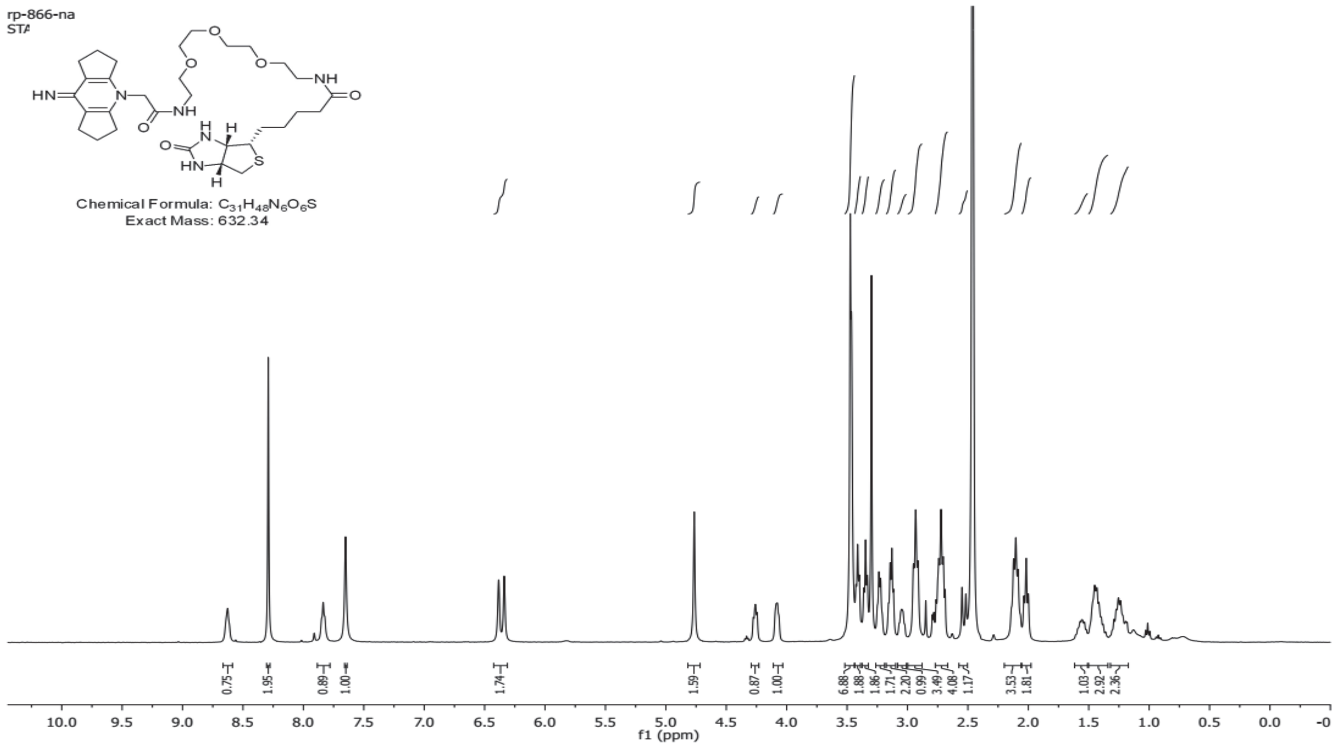


## $^1$H NMR spectrum of compound biotin-Cmpd 1

**t**

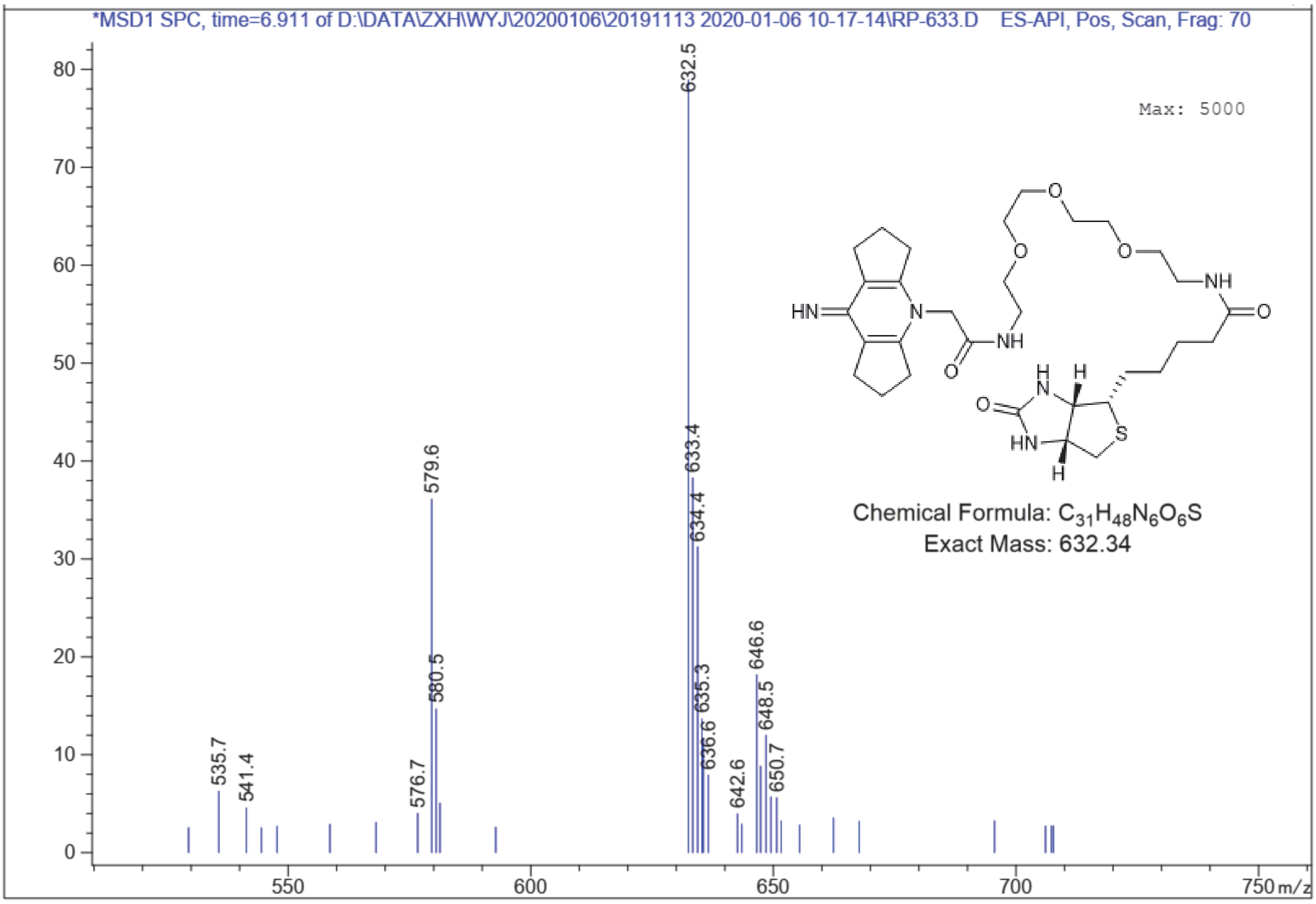


## Mass spectrum of compound biotin-Cmpd 1

**Supplementary Fig. 3** Synthesis and characterization of CCVJ-Cmpd **1**, biotin-Cmpd **1** and associated intermediate compounds. **a-c** Scheme outlining synthesis of CCVJ-Cmpd **1** (**a**) , biotin-Cmpd **1** (**b**) , intermediate I-7 (**c**). **d-q** NMR or mass spectrum of associated intermediate compounds. **r-t** NMR or mass spectrum of CCVJ-Cmpd **1** (**r**) , biotin-Cmpd **1** (**s-t**).

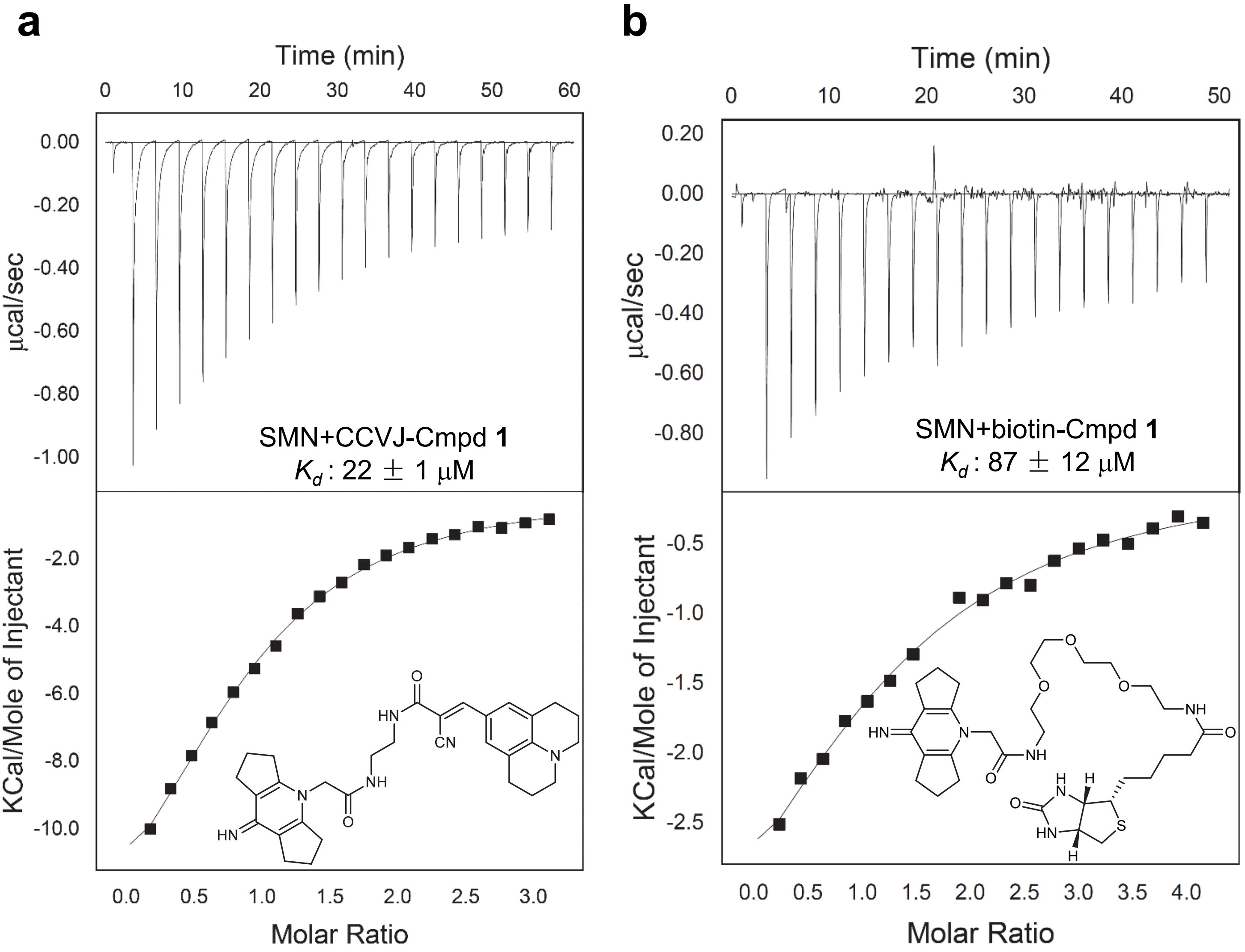


**Supplementary Fig. 4** Modified compound **1**s still bind to Tudor domain of SMN. ITC binding curves for the titration of CCVJ-Cmpd **1** (**a**) or biotin-Cmpd **1** (**b)** to the Tudor domain of SMN, respectively. ITC data shown are representative of two independent experiments.

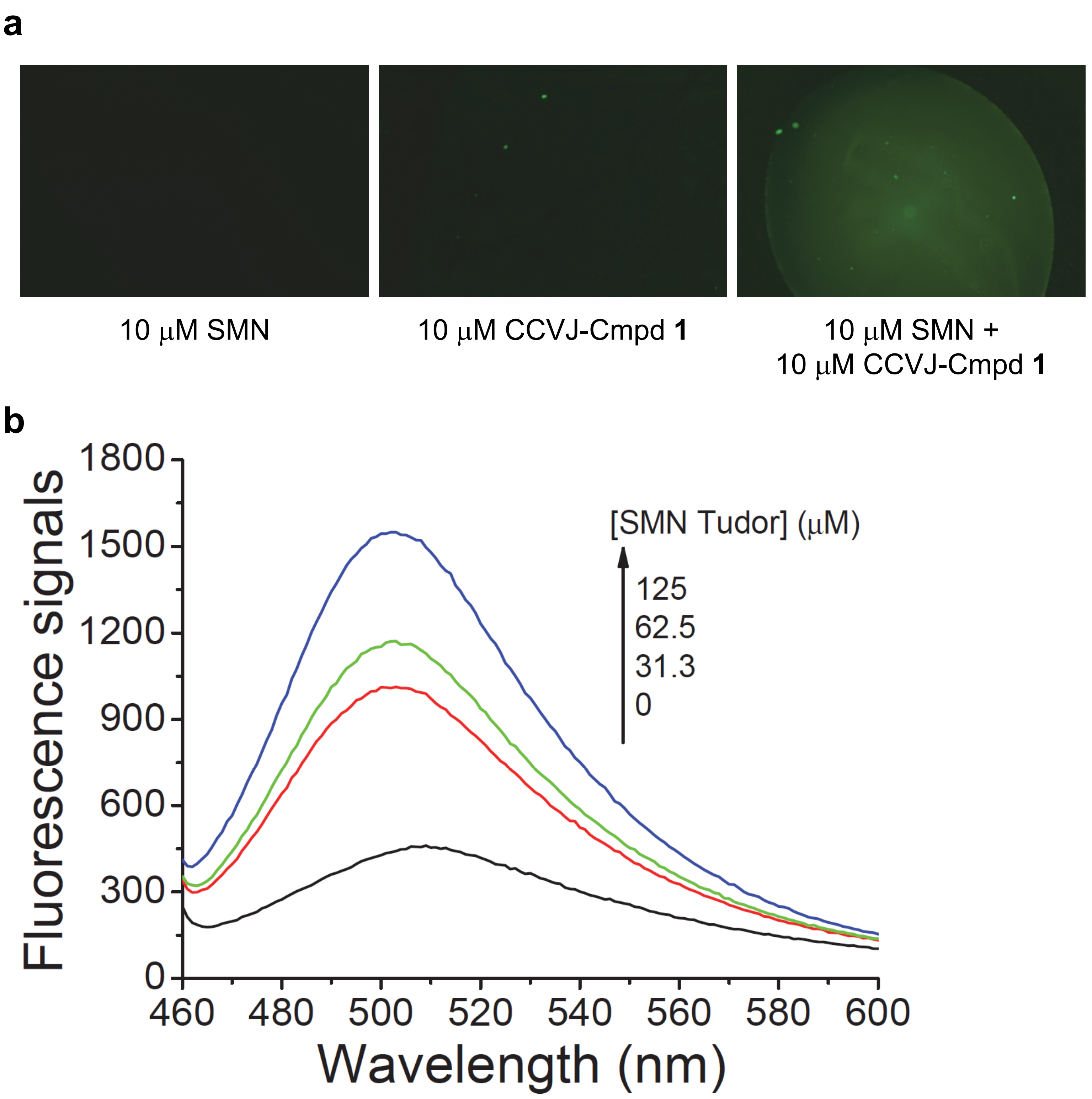


**Supplementary Fig. 5** Interaction between Tudor domain of SMN and CCVJ-Cmpd **1** triggers fluorescence signals. **a** Fluorescence signals of 10 μM SMN Tudor, 10 μM CCVJ-Cmpd **1**, and mixture of 10 μM SMN Tudor with 10 μM CCVJ-Cmpd **1** were detected by fluorescence microscope (Nikon, ECLIPSE Ti-E), under the excitation of blue light. The CCVJ-Cmpd **1** only showed weak fluorescence signal, which was enhanced by addition of the SMN Tudor domain protein. Exposure time: 1 s. **b** Fluorescence signals for addition of different concentrations of the SMN Tudor domain protein to 5 μM CCVJ-Cmpd **1** detected by Infinite M1000 Pro microplate reader (Tecan) with an excitation wavelength of 440 nm and an emission wavelength of 460-600 nm. Data shown are representative of three independent experiments.

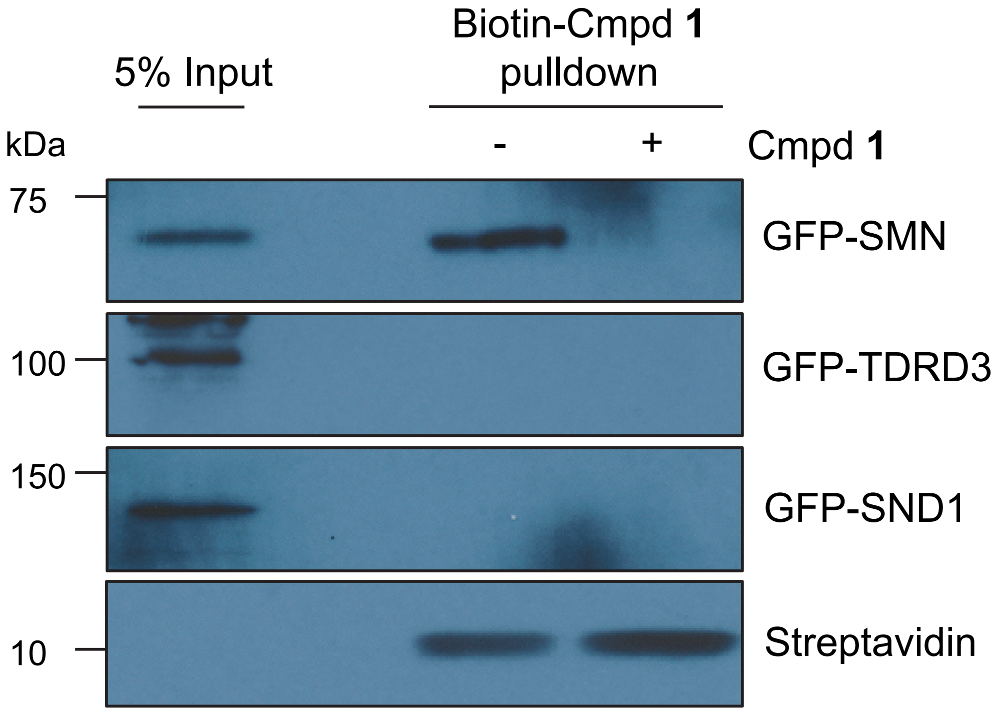


**Supplementary Fig. 6** Biotin-Cmpd **1** selectively chemiprecipitates SMN but not TDRD3 or SND1, two other Tudor domain-containing proteins from GFP-SMN/TDRD3/SND1 co-transfected U2OS cells. Data shown are representative of three independent experiments. Source data are provided as a Source Data file.

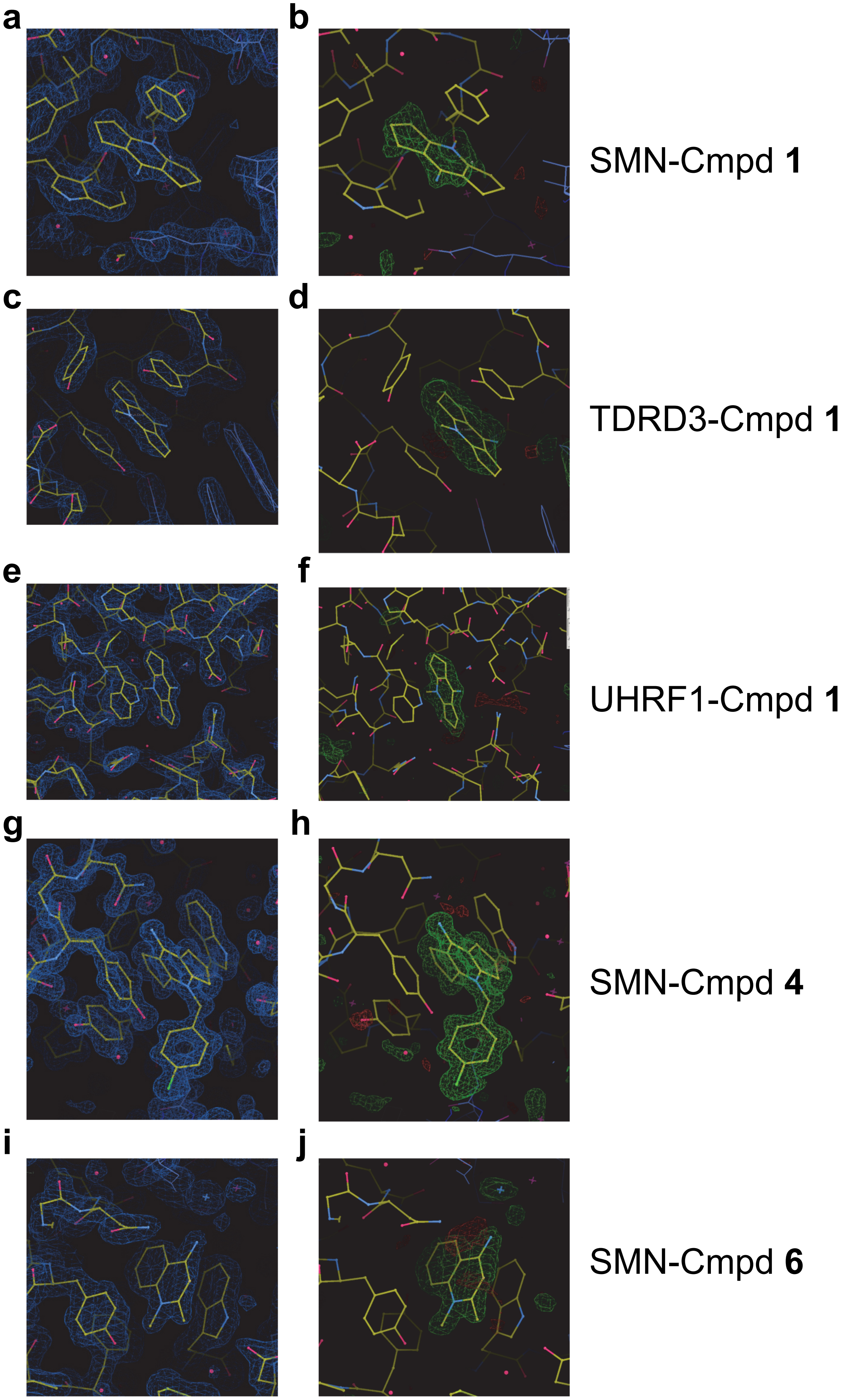


**Supplementary Fig. 7** Portion of the electron density map and omit map of different complexes. **a, c, e, g, i,** 2Fo-Fc electron density map contoured at 3σ. **b, d, f, h, j,** Fo-Fc omit map contoured at 1σ.

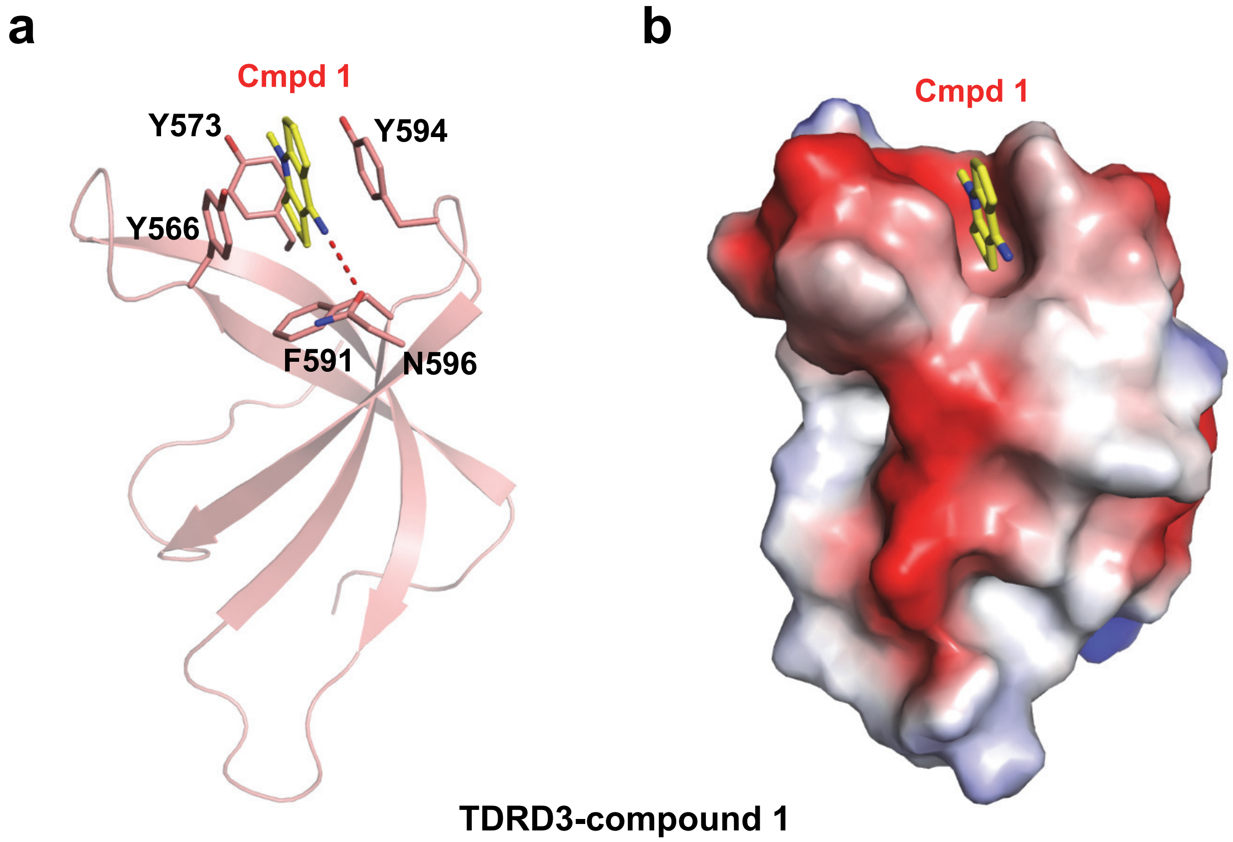


**Supplementary Fig. 8** Complex structure of Tudor domain of TDRD3 and compound **1**. **a** The TDRD3 complex structure is shown in a cartoon mode. The Tudor domain of TDRD3 is colored in salmon, with the interacting residues shown in sticks and the intermolecular hydrogen bonds shown in red dashes. **b** Electrostatic potential surface representation of the complex.

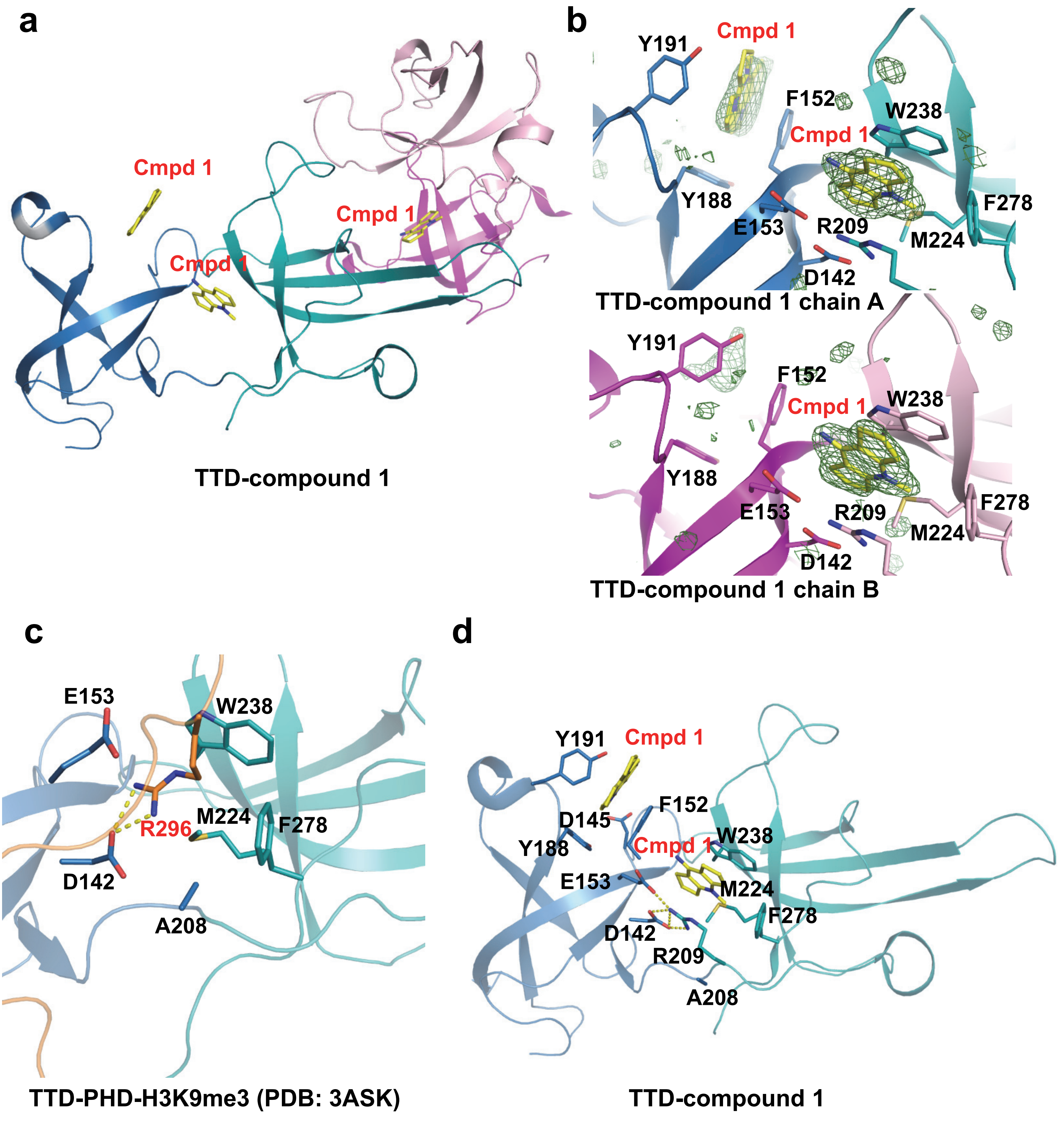


**Supplementary Fig**. 9 The aromatic cage of UHRF1 is not the binding site for compound **1**. (To be continued)

**e**

| Wild type or mutants | $K_d$ (μM) |
|---|---|
| UHRF1_WT | 16 ± 1 |
| UHRF1_D145A | 16 ± 1 |
| UHRF1_F152A | 17 ± 2 |
| UHRF1_Y188A | 6.1 ± 1.2 |
| UHRF1_Y191A | 14 ± 1 |
| UHRF1_F152A/Y191A | 12 ± 1 |
| UHRF1_R209A | NB |
| UHRF1_F278A | NB |
| UHRF1_D142A | NB |
| UHRF1_E153A | WB |
| UHRF1_W238A | insoluble |

NB, no detectable binding; WB, weak binding.

**f**

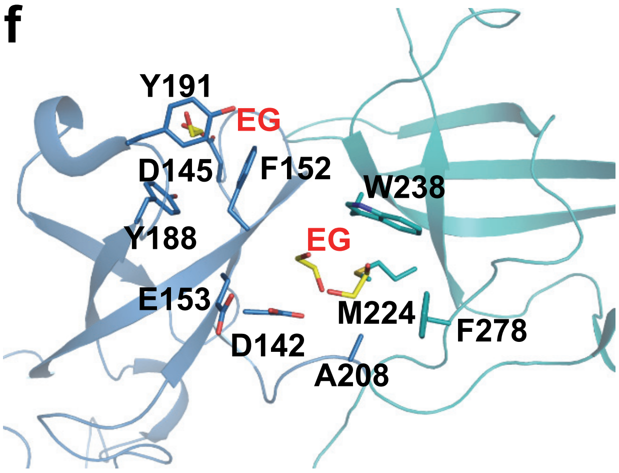


**g**

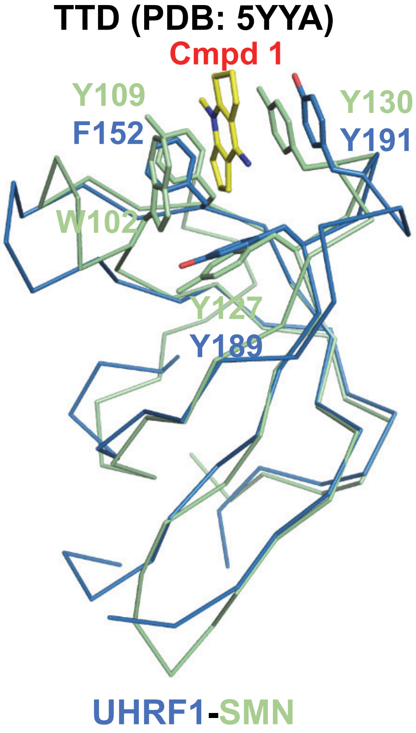


**Supplementary Fig. 9** The aromatic cage of UHRF1 is not the binding site for compound **1**. **a** Overall structure of TTD domain of UHRF1 in complex with compound **1**. The $TTD_N$ and $TTD_C$ of the two UHRF1 molecules were colored in blue, green (chain A) and magenta, pink (chain B), respectively. **b** Complex structure of UHRF1_TTD-compound **1** with the Fo–Fc omit map of compound **1** contoured at 3 σ and TTD shown in a ribbon mode. **c** Complex structure of TTD-PHD of UHRF1 with an H3K9me3 peptide (PDB: 3ASK). The binding pocket of residue R296 from the TTD-PHD linker is shown in a stick mode and the intramolecular hydrogen bonds were shown in yellow dashes. **d** Complex structure of TTD of UHRF1 and compound **1** is shown in a cartoon mode with the interacting residues shown in sticks and the intramolecular hydrogen bonds indicated by yellow dashes. **e** Binding affinities of compound **1** to different UHRF1 TTD mutants determined by ITC. ITC data shown are representative of two independent experiments. **f** Apo structure of the UHRF1 TTD domain with small molecules (EG, ethylene glycol from the crystallization buffer) located in the H3K9me3 and arginine binding pockets. **g** Comparison of the methyllysine binding cage of UHRF1 to the methylarginine binding cage of SMN. The cage forming residues and compound **1** are shown in sticks. Source data are provided as a Source Data file.

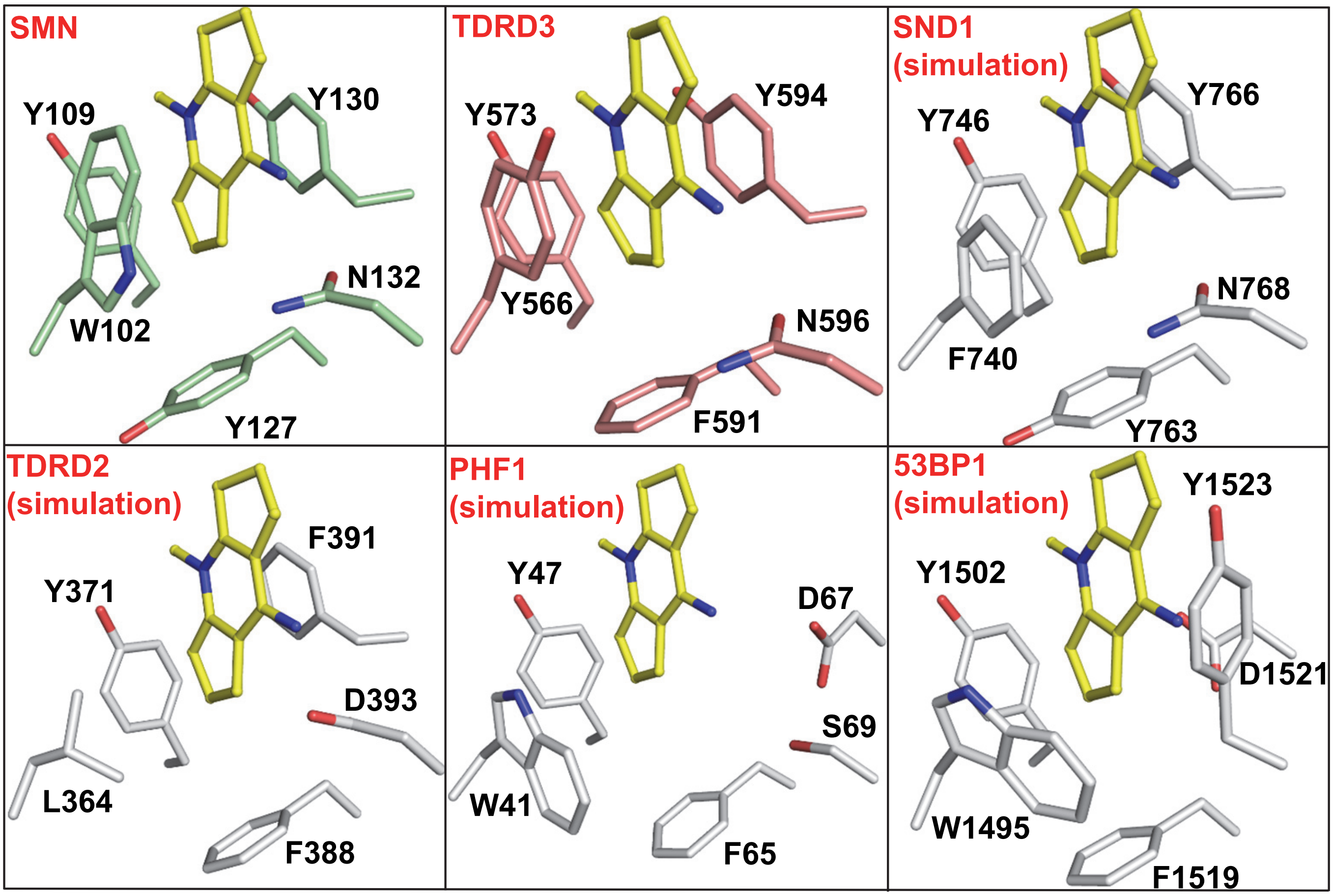


**Supplementary Fig. 10** Complex structures or complex models of different Tudor domains with compound **1**. The complex models were built based on superposition of different Tudor domain structures onto the SMN-compound **1** complex structure.

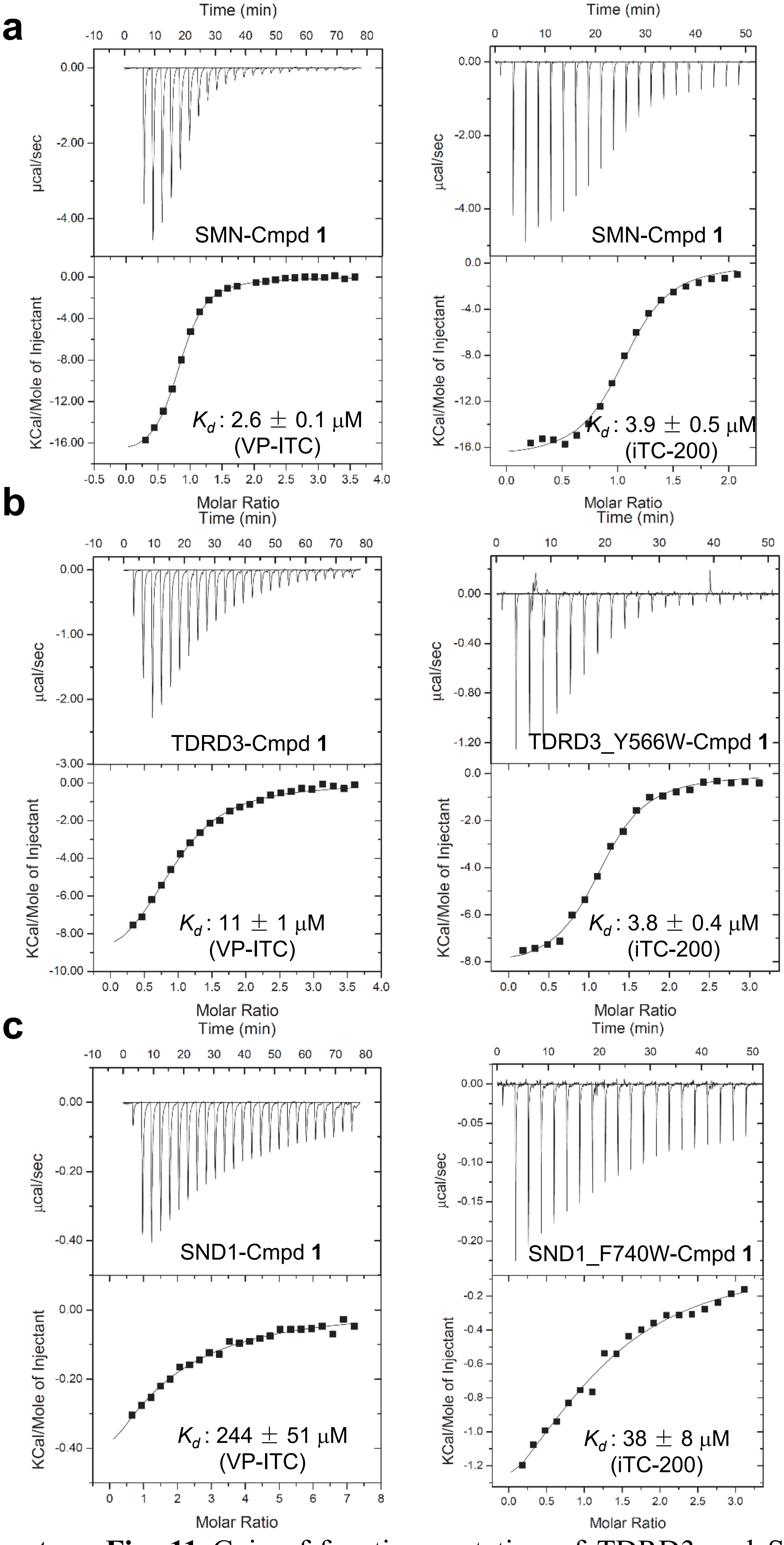


**Supplementary Fig. 11** Gain-of-function mutation of TDRD3 and SND1 increases the binding affinity to compound **1**. **a** Similar binding affinities of SMN-Cmpd **1** measured by VP-ITC (left) or iTC-200 (right). **b, c** Comparison of ITC data of wild-type and mutant of TDRD3 and SND1, respectively. ITC data shown are representative of two independent experiments.

## Group A

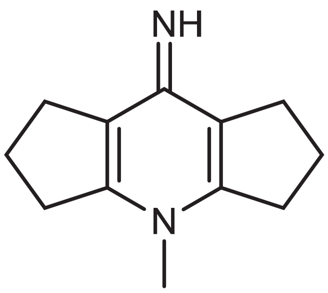


compound **1**

4-methyl-2,3,4,5,6,7-hexahydrodicyclopenta[*b*,*e*]pyridin-8(1*H*)-imine

**$K_d$: 2.6 μM**

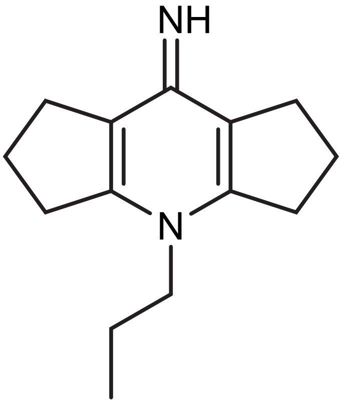


compound **2**

4-propyl-2,3,4,5,6,7-hexahydrodicyclopenta[*b*,*e*]pyridin-8(1*H*)-imine

**$K_d$: 9.3 μM**

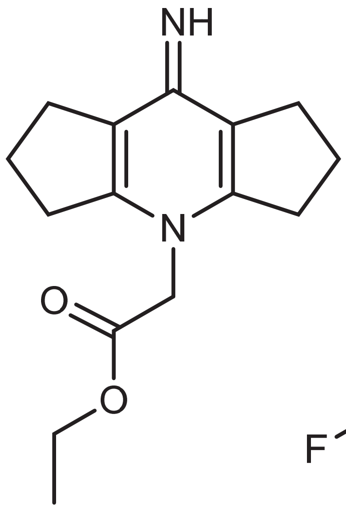


compound **3**

ethyl 2-(8-imino-2,3,5,6,7,8-hexahydrodicyclopenta[*b*,*e*]pyridin-4(1*H*)-yl)acetate

**$K_d$: 31 μM**

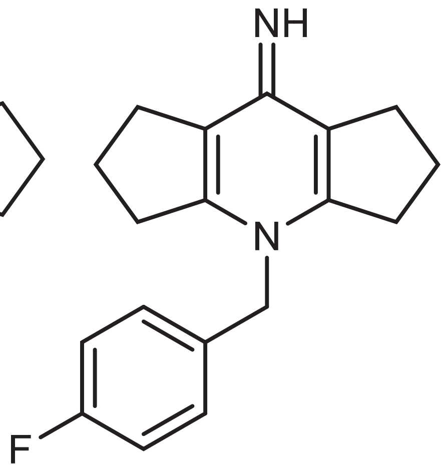


compound **4**

4-(4-fluorobenzyl)-2,3,4,5,6,7-hexahydrodicyclopenta[*b*,*e*]pyridin-8(1*H*)-imine

**$K_d$: 13 μM**

**Supplementary Fig. 12** Chemical structure and binding affinity (determined by ITC) of different compounds to SMN. (To be continued)

# Group B

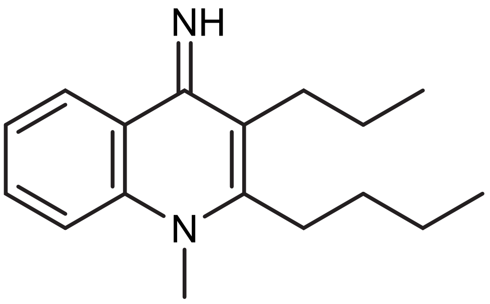

compound **5**

2-butyl-1-methyl-3-propylquinolin-4(1*H*)-imine

***K*$_d$: 12 μM**

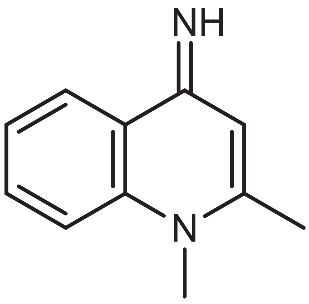

compound **6**

1,2-dimethylquinolin-4(1*H*)-imine

***K*$_d$: 12 μM**

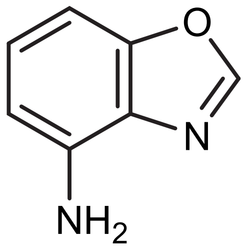

compound **7**

benzo[*d*]oxazol-4-amine

**NB**

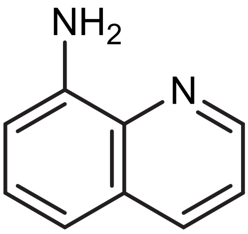

compound **8**

quinolin-8-amine

**NB**

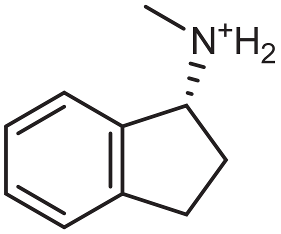

compound **9**

(*R*)-N-methyl-2,3-dihydro-1*H*-inden-1-aminium

**NB**

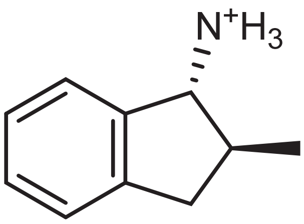

compound **10**

(1*R*,2*S*)-2-methyl-2,3-dihydro-1*H*-inden-1-aminium

**NB**

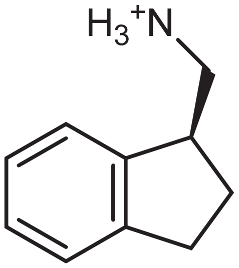

compound **11**

(*S*)-(2,3-dihydro-1*H*-inden-1-yl)methanaminium

**NB**

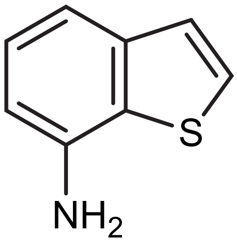

compound **12**

benzo[*b*]thiophen-7-amine

**NB**

**Supplementary Fig. 12** Chemical structure and binding affinity (determined by ITC) of different compounds to SMN. (To be continued)

# Group B

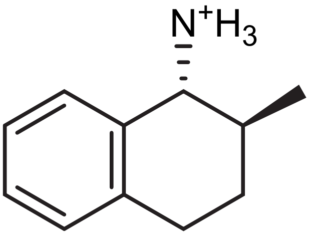

compound **13**

(1*R*,2*S*)-2-methyl-1,2,3,4-tetrahydronaphthalen-1-aminium

**NB**

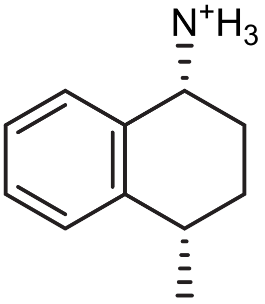

compound **14**

(1*R*,4*S*)-4-methyl-1,2,3,4-tetrahydronaphthalen-1-aminium

**NB**

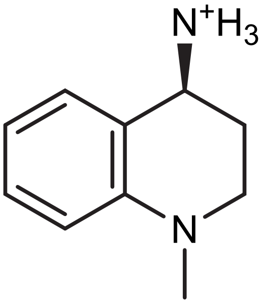

compound **15**

(*S*)-1-methyl-1,2,3,4-tetrahydroquinolin-4-aminium

**NB**

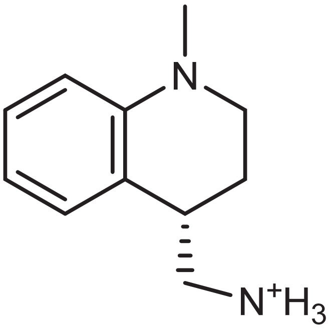

compound **16**

(*S*)-(1-methyl-1,2,3,4-tetrahydroquinolin-4-yl)methanaminium

**NB**

# Group C

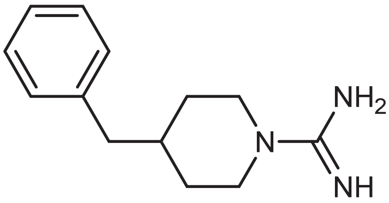

compound **17**

4-benzylpiperidine-1-carboximidamide

**NB**

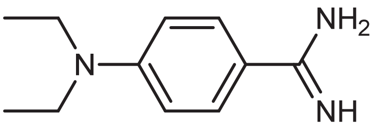

compound **18**

4-(diethylamino)benzimidamide

**NB**

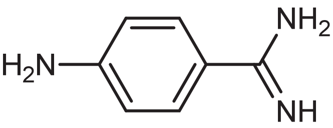

compound **19**

4-aminobenzimidamide

**NB**

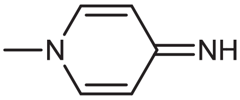

compound **20**

1-methylpyridin-4(1*H*)-imine

**NB**

**Supplementary Fig. 12** Chemical structure and binding affinity (determined by ITC) of different compounds to SMN.

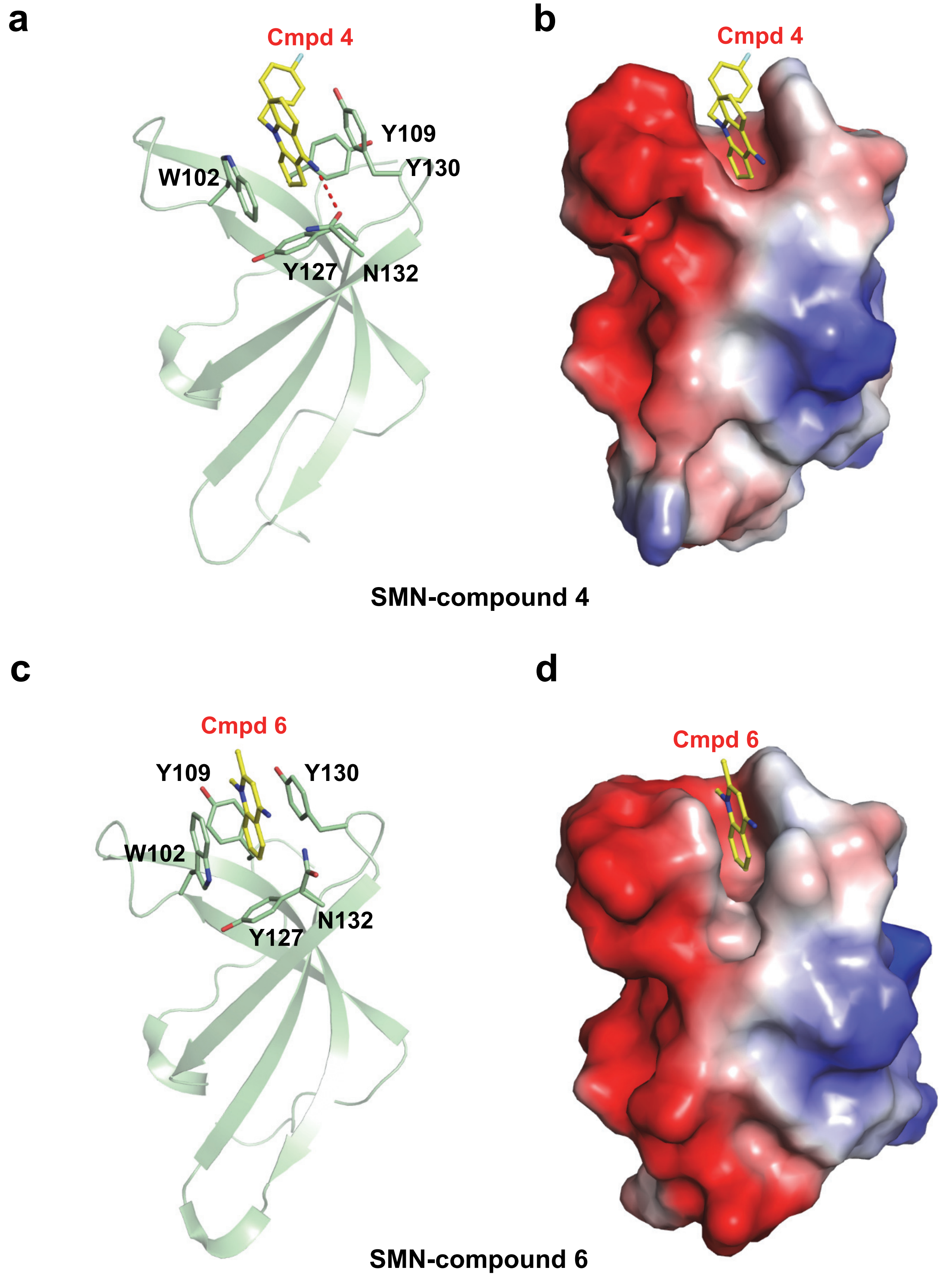


**Supplementary Fig. 13** Complex structures of SMN-compound **4** and SMN-compound **6**. Complex structure of Tudor domain of SMN and compound **4** or **6** shown in a cartoon mode (**a** or **c**) and electrostatic potential surface representation (**b** or **d**), respectively.

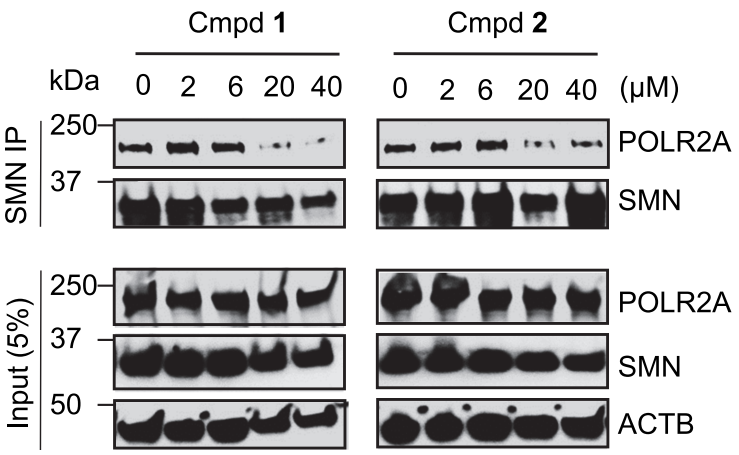


**Supplementary Fig. 14** The SMN antagonists disrupt binding of SMN to RNAP II. IP-western blot experiments were performed by using the indicated antibodies in HEK293 cel lextract treated with indicated concentrations of compound **1**, compound **2**, or DMSO for 7 2h. Data shown are representative of three independent experiments.
Source data are provided as a Source Data file.

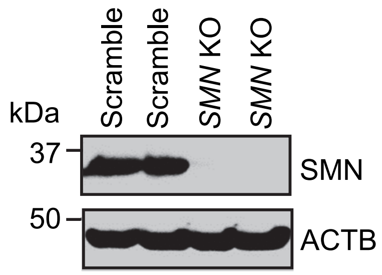


**Supplementary Fig. 15** The efficiency of knockout of SMN by CRISPR/Cas9 wa sexamined by western blot analysis. Data shown are representative of three independe ntexperiments. Source data are provided as a Source Data file.

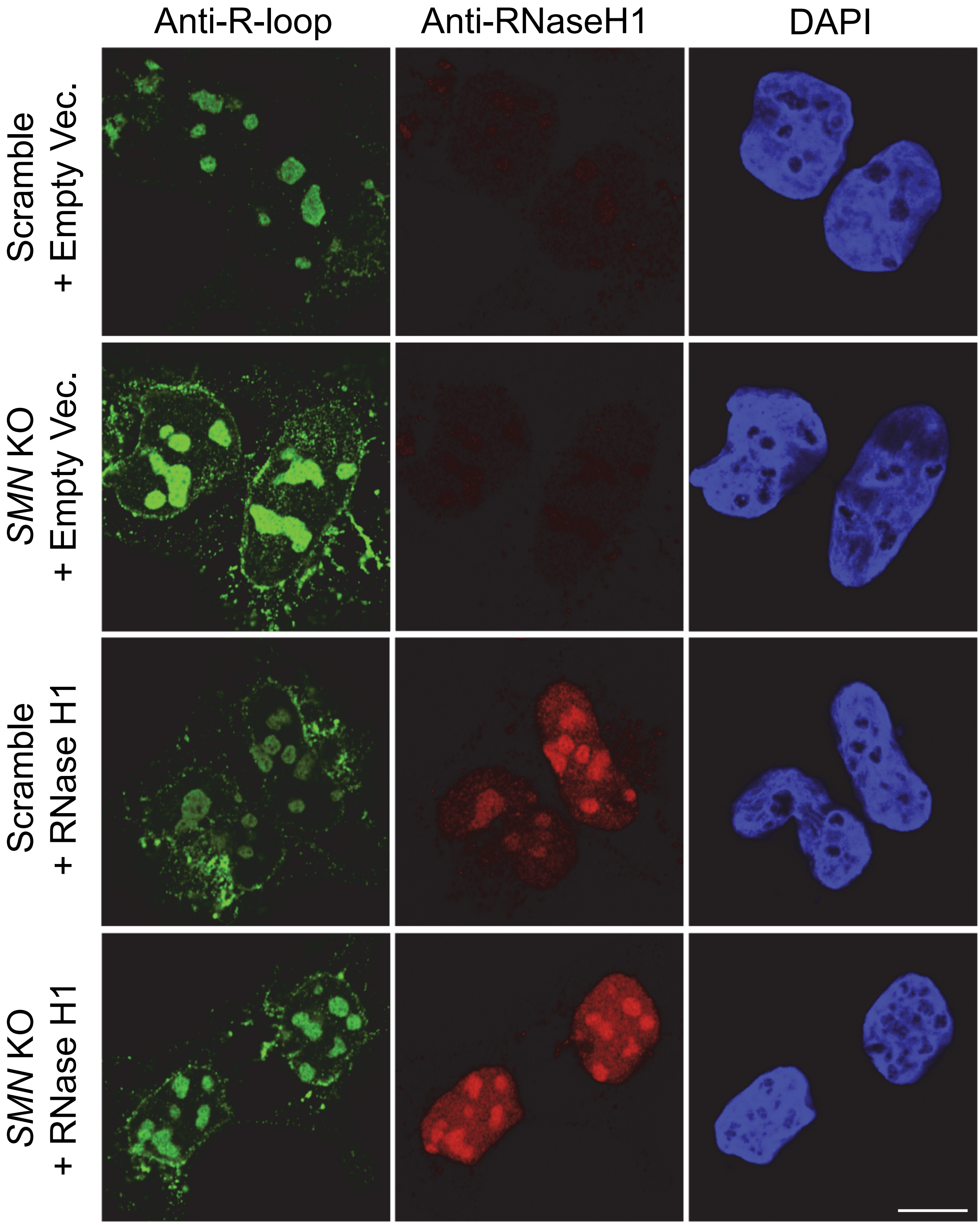


**Supplementary Fig. 16** RNase H1 overexpression decreases R-loop accumulation. Double immunofluorescence coupled to confocal microscopy analysis of R-loop staining in *SMN*-knockout HEK293 cells expressing human RNase H1 or empty control vector. Shown are representative confocal images of three independent experiments. Scale bar: 5 μm.

**Supplementary Table 1.** Small molecule screening data

| Category | Parameter | Description |
|---|---|---|
| Assay | Type of assay | *in vitro* |
| | Target | Screening inhibitors against the histone H3K9me3 binding tandem Tudor domain (TTD) of UHRF1 (uniport ID: Q96T88) |
| | Primary measurement | Fluorescein polarization-based peptide displacement assay |
| | Key reagents | FITC-labeled H3K9me3 peptide (aa 1-25, synthesized by Tufts University Core Services), Fragment library consisting of 2040 compounds comprising 1000 compounds from Maybridge Ro3 Core fragment library supplemented by an additional 1040 compounds from Sigma-Aldrich and Specs. |
| | Assay protocol | A small molecule fragment library with 2040 compounds was screened against tandem Tudor domain of UHRF1 by fluorescein polarization-based peptide displacement assay according to previous reports[2]. Briefly, the screening was performed in 10 μL at a protein concentration of 8 μM premixed with a 40 nM FITC-labeled H3K9me3 peptide (aa 1-25, Tufts University Core Services), and then adding a single concentration of 2 mM compound in a buffer of 20 mM Tris-HCl, pH 8.8, 50 mM NaCl, and 0.01% Triton X-100. The hits were further confirmed by dose response analysis with 1 mM as the highest concentration with 11 sequential 2-fold dilutions. All the assays were performed in duplicate in 384-well plates (Greiner, 784290), using the Synergy 4 microplate reader (BioTek), with an excitation wavelength of 485 nm and an emission wavelength of 528 nm. Data were corrected by background of the free labeled peptides and analyzed by GraphPad Prism version 5 software. All the compounds were purchased from Maybridge, Sigma or Specs company. |
| | Additional comments | |
| Library | Library size | 2040 |
| | Library composition | 1000-compound Maybridge Ro3 Core of fragment library (SOLiD™ Fragment Library Construction Reagents, #4443713) supplemented by 1040 selected compounds from Sigma-Aldrich and Specs. |
| | Source | Commercial compounds from Maybridge, Sigma or |

| | | |
|---|---|---|
| | | Specs company |
| | Additional comments | |
| Screen | Format | 384-well black plates (Greiner, 784290) |
| | Concentration(s) tested | 2 mM compound with 2% DMSO (final concentration), 40 nM FITC-labeled H3K9me3 peptide, 8 μM tandem Tudor domain of UHRF1 protein |
| | Plate controls | Background: FITC-labeled peptide only; negative control: FITC-labeled peptide and protein; positive control: FITC-labeled peptide and protein plus unlabeled H3K9me3 peptide |
| | Reagent/ compound dispensing system | Agilent Bravo robot |
| | Detection instrument and software | Synergy 4 microplate reader (BioTek), GraphPad Prism version 5 software |
| | Assay validation/QC | Z' factor = 0.8 |
| | Correction factors | Background value |
| | Normalization | All the digital values of fluorescence polarization signal subtract the background signal value. |
| | Additional comments | High concentrations (>2.5%) of DMSO might affect peptide binding and signal-to-noise ratios significantly. |
| Post-HTS analysis | Hit criteria | The signal of negative control was set as 100%. When the signal of sample was less than 50% of negative control, it was taken as hit. |
| | Hit rate | 5/2040 |
| | Additional assay(s) | The hits were further confirmed by dose response analysis with 1 mM as the highest concentration of the hits and 11 serial dilutions by 2 times, and then by isothermal titration calorimetry assay. |
| | Confirmation of hit purity and structure | The compound was repurchased (Specs, ID: AG-690/33357017), structure and purity was verified analytically. |
| | Additional comments | The hit compound (namely compound **1** in our manuscript) could be only dissolved in DMSO with a high concentration (100 mM) but not in buffer. |

**Supplementary Table 2** Primer sequences

| Protein fragments | Sequences |
|---|---|
| *E coli* expression constructs[a] | |
| SMN (aa 82-147) | 5': ttgtatttccagggcAAGAAGAATACTGCAGCTTCC |
| | 3': caagcttcgtcatcaTTCACAGATTGGGGAAAGTAG |
| UHRF1 (aa 126-285) | 5': ttgtatttccagggcATGTGGGATGAGACGGAATTG |
| | 3': caagcttcgtcatcaTTCACCCGGCCGCTCAATC |
| SMNDC1 (aa 53-130) | 5': ttgtatttccagggcGCTTCTACTCAACCTACTCATTC |
| | 3': caagcttcgtcatcaCTTCCTTCCTTCTTCTACAGG |
| TDRD3 (aa 554-611) | 5': ttgtatttccagggcAAAATGTGGAAACCTGGAGATG |
| | 3': caagcttcgtcatcaCTCTGTTTGAATGGGCTTGATATT |
| SND1 (aa 650-910) | 5': ttgtatttccagggcGCAAAGCAGAAGAAAGAGAAGG |
| | 3': caagcttcgtcatcaGCGGCTGTAGCCAAATTCGTC |
| TDRD2 (aa 327-420) | 5': ttgtatttccagggcGGCTCCCGCAGCCTGCAATTG |
| | 3': caagcttcgtcatcaACTACATTCTATTGCTTGAAATG |
| FXR1 (aa 2-132) | 5': ttgtatttccagggcGCGGAGCTGACGGTGGAG |
| | 3': caagcttcgtcatcaATCCACTGTGCATTTAAAGAAGG |
| PHF1 (aa 28-87) | 5': ttgtatttccagggcAGGCCTCGGCTTTGGGAGG |
| | 3': caagcttcgtcatcaTTCCTCTCCAGGGAGGGCAG |
| SGF29 (aa 115-293) | 5': ttgtatttccagggcCGCAGAGGGGTGCTGATGAC |
| | 3': caagcttcgtcatcaCTTTTTCTTGGGTTCCTTACAAG |
| JMJD2A (aa 897-1101) | 5': ttgtatttccagggcCAAAGCATCACTGCAGGCCAG |
| | 3': caagcttcgtcatcaCTACTCCATGATGGCCCGG |
| 53BP1 (aa 1483-1606) | 5': ttgtatttccagggcGGAAATAGCTTTGTAGGGCTC |
| | 3': caagcttcgtcatcaTTCATAGGGGCCAAGCCCATAC |
| SETDB1 (aa 190-410) | 5': ttgtatttccagggcGGAGAACTAAGCAAAGATGGTG |
| | 3': caagcttcgtcatcaCTCCAGTGCAGAGGCTGAG |
| LBR (aa 1-67) | 5': ttgtatttccagggcATGCCAAGTAGGAAATTTGCCG |
| | 3': caagcttcgtcatcaTGAGCCACCTTTCCTTTGCC |
| ZGPAT (aa 120-271) | 5': ttgtatttccagggcGAGGAAGAGGGAGAGGACG |
| | 3': caagcttcgtcatcaTGTGCGCAGTGGGGGCAG |
| CBX7 (aa 8-62) | 5': ttgtatttccagggcGAGCAGGTGTTCGCCGTGG |
| | 3': caagcttcgtcatcaCTCCTCCTTCTCCTCGTAGG |
| DNMT3A (aa 275-417) | 5': ttgtatttccagggcGGCGATGACGAGCCAGAGTAC |
| | 3': caagcttcgtcatcaTTCTTCTGGTGGCTCCAGGC |
| WDR5 (aa 24-334) | 5': ttgtatttccagggcACTCAGAGCAAGCCTACACC |
| | 3': caagcttcgtcatcaGCAGTCACTCTTCCACAGTTTAA |
| ZCWPW2 (aa 21-78) | 5': ttgtatttccagggcGTGGAAAACATGTATGTAAACAA |
| | 3': caagcttcgtcatcaGAAGTCTTCTTCAGAAATTGAGC |
| L3MBTL1 (aa 200-522) | 5': gttccgcgtggtagtGGTGAGAAGAAGGAATGCTGG |
| | 3': caagcttcgtcatcaCTCTCTGGGTCCGAGAGGAG |
| L3MBTL2 (aa 170-625) | 5': gttccgcgtggtagtACAGGACAAGACGCTCTGGTC |

| | |
|---|---|
| | 3': caagcttcgtcatcaCTGTTTCTTTTTCTTCTTTGTGG |
| Mutants[b] | |
| SMN1_W102A | 5': AAATGTTCTGCCATTgcgTCAGAAGACGGTTGC |
| | 3': GCAACCGTCTTCTGAcgcAATGGCAGAACATTT |
| SMN1_W102F | 5': AAATGTTCTGCCATTttcTCAGAAGACGGTTGC |
| | 3': GCAACCGTCTTCTGAgaaAATGGCAGAACATTT |
| SMN1_W102Y | 5': AAATGTTCTGCCATTtacTCAGAAGACGGTTGC |
| | 3': GCAACCGTCTTCTGAgtaAATGGCAGAACATTT |
| SMN1_Y109A | 5': GAAGACGGTTGCATTgccCCAGCTACCATTGCT |
| | 3': AGCAATGGTAGCTGGggcAATGCAACCGTCTTC |
| SMN1_Y127A | 5': ACCTGTGTTGTGGTTgccACTGGATATGGAAAT |
| | 3': ATTTCCATATCCAGTggcAACCACAACACAGGT |
| SMN1_Y127F | 5': ACCTGTGTTGTGGTTttcACTGGATATGGAAAT |
| | 3': ATTTCCATATCCAGTgaaAACCACAACACAGGT |
| SMN1_Y130A | 5': GTGGTTTACACTGGAgctGGAAATAGAGAGGAG |
| | 3': CTCCTCTCTATTTCCagcTCCAGTGTAAACCAC |
| SMN1_N132A | 5': TACACTGGATATGGAgctAGAGAGGAGCAAAAT |
| | 3': ATTTTGCTCCTCTCTagcTCCATATCCAGTGTA |
| SMN1_N132D | 5': TACACTGGATATGGAgatAGAGAGGAGCAAAAT |
| | 3': ATTTTGCTCCTCTCTatcTCCATATCCAGTGTA |
| UHRF1_D145A | 5': TACGTCGATGCTCGGgccACGAACATGGGGGCG |
| | 3': CGCCCCCATGTTCGTggcCCGAGCATCGACGTA |
| UHRF1_F152A | 5': AACATGGGGGCGTGGgctGAGGCGCAGGTGGTC |
| | 3': GACCACCTGCGCCTCagcCCACGCCCCCATGTT |
| UHRF1_Y188A | 5': ATTTACCACGTGAAAgccGACGACTACCCGGAG |
| | 3': CTCCGGGTAGTCGTCggcTTTCACGTGGTAAAT |
| UHRF1_Y191A | 5': GTGAAATACGACGACgccCCGGAGAACGGCGTG |
| | 3': CACGCCGTTCTCCGGggcGTCGTCGTATTTCAC |
| UHRF1_R209A | 5': GTCCGAGCGCGCGCCgccACCATCATCAAGTGG |
| | 3': CCACTTGATGATGGTggcGGCGCGCGCTCGGAC |
| UHRF1_F278A | 5': TTCGTGGACGAAGTCgccAAGATTGAGCGGCCG |
| | 3': CGGCCGCTCAATCTTggcGACTTCGTCCACGAA |
| UHRF1_D142A | 5': GTCAATGAGTACGTCgctGCTCGGGACACGAAC |
| | 3': GTTCGTGTCCCGAGCagcGACGTACTCATTGAC |
| UHRF1_E153A | 5': ATGGGGGCGTGGTTTgcgGCGCAGGTGGTCAGG |
| | 3': CCTGACCACCTGCGCcgcAAACCACGCCCCCAT |
| UHRF1_W238A | 5': AAGGAGCGGGGCTTCgcgTACGACGCGGAGATC |
| | 3': GATCTCCGCGTCGTAcgcGAAGCCCCGCTCCTT |
| TDRD3_Y566W | 5': GAATGTTTTGCACTTtatTGGGAAGACAACAAG |
| | 3': CTTGTTGTCTTCCCAataAAGTGCAAAACATTC |
| SND1_F740W | 5': TTCTGCATTGCCAAAtttGTAGATGGAGAATGG |
| | 3': CCATTCTCCATCTACaaaTTTGGCAATGCAGAA |

| | |
|---|---|
| Mammalian expression constructs[c] | |
| SMN_full length | 5': cccaagcttATGGCGATGAGCAGCGGCG<br>3': cgcggatccATTTAAGGAATGTGAGCACCTTC |
| SND1_full length | 5': cccaagcttATGGCGTCCTCCGCGCAG<br>3': cgcggatccGCGGCTGTAGCCAAATTCGTC |
| TDRD3_full length | 5': cccaagcttATGCTGCGATTACAGATGACTG<br>3': cgcggatccGTTCCGAGCCCGGGGTGG |
| qPCR[3] | |
| *ACTB* (-72) | 5': CCGAAAGTTGCCTTTTATGGC<br>3': CAAAGGCGAGGCTCTGTGC |
| *ACTB* (332) | 5': CGGGGTCTTTGTCTGAGC<br>3': CAGTTAGCGCCCAAAGGAC |
| *ACTB* (1671) | 5': TAACACTGGCTCGTGTGACAA<br>3': AAGTGCAAAGAACACGGCTAA |
| *ACTB* (2911) | 5': TGCGCAGAAAACAAGATGAG<br>3': GTCACCTTCACCGTTCCAGT |
| *ACTB* (3560) | 5': TTACCCAGAGTGCAGGTGTG<br>3': CCCCAATAAGCAGGAACAGA |
| *ACTB* (3752) | 5': GGGACTATTTGGGGGTGTCT<br>3': TCCCATAGGTGAAGGCAAAG |
| *ACTB* (4657) | 5': TGGGCCACTTAATCATTCAAC<br>3': CCTCACTTCCAGACTGACAGC |
| *ACTB* (5590) | 5': CAGTGGTGTGGTGTGATCTTG<br>3': GGCAAAACCCTGTATCTGTGA |

Note: a, the lower cases of these primers are the sequences used by T4 ligase-independent cloning; b, the lower cases of these primers are the mutation sites; c, the lower cases of these primers are the sequences associated with restriction enzyme sites, *Hind* III at 5' primer and *Bam*H I at 3' primer.

## Supplementary References


1. Yu, W.T., Wu, T.W., Huang, C.L., Chen, I.C. & Tan, K.T. Protein sensing in living cells by molecular rotor-based fluorescence-switchable chemical probes. *Chem. Sci.* **7**, 301-307 (2016).
2. Senisterra, G. et al. Discovery of small-molecule antagonists of the H3K9me3 binding to UHRF1 tandem Tudor domain. *SLAS Discov.* **23**, 930-940 (2018).
3. Zhao, D.Y. et al. SMN and symmetric arginine dimethylation of RNA polymerase II C-terminal domain control termination. *Nature* **529**, 48-53 (2016).
