# Supplementary material for: A small molecule antagonist of SMN disrupts the interaction between SMN and RNAP II
Source: Nat Commun. 2022 Sep 16;13:5453. doi: 10.1038/s41467-022-33229-5 (PMC9481570; doi:10.1038/s41467-022-33229-5)
Supplement: Supplementary file 2 — Reporting Summary [file 41467_2022_33229_MOESM2_ESM.pdf]

nature portfolio

Corresponding author(s): Yanli Liu, Jinrong Min

Last updated by author(s): Aug 7, 2022

Reporting Summary

Nature Portfolio wishes to improve the reproducibility of the work that we publish. This form provides structure for consistency and transparency in reporting. For further information on Nature Portfolio policies, see our Editorial Policies and the Editorial Policy Checklist.

Statistics

For all statistical analyses, confirm that the following items are present in the figure legend, table legend, main text, or Methods section.

n/a	Confirmed	
☐	☒	The exact sample size (*n*) for each experimental group/condition, given as a discrete number and unit of measurement
☐	☒	A statement on whether measurements were taken from distinct samples or whether the same sample was measured repeatedly
☐	☒	The statistical test(s) used AND whether they are one- or two-sided<br>*Only common tests should be described solely by name; describe more complex techniques in the Methods section.*
☒	☐	A description of all covariates tested
☒	☐	A description of any assumptions or corrections, such as tests of normality and adjustment for multiple comparisons
☐	☒	A full description of the statistical parameters including central tendency (e.g. means) or other basic estimates (e.g. regression coefficient) AND variation (e.g. standard deviation) or associated estimates of uncertainty (e.g. confidence intervals)
☐	☒	For null hypothesis testing, the test statistic (e.g. *F*, *t*, *r*) with confidence intervals, effect sizes, degrees of freedom and *P* value noted<br>*Give P values as exact values whenever suitable.*
☒	☐	For Bayesian analysis, information on the choice of priors and Markov chain Monte Carlo settings
☒	☐	For hierarchical and complex designs, identification of the appropriate level for tests and full reporting of outcomes
☒	☐	Estimates of effect sizes (e.g. Cohen's *d*, Pearson's *r*), indicating how they were calculated

Our web collection on statistics for biologists contains articles on many of the points above.

Software and code

Policy information about availability of computer code

Data collection	Diffraction images were collected on a copper rotating anode source for SMN-compound 1 and SMN-compound 6, respectively; diffraction data were collected at APS/NE-CAT beam line 24-ID-E, at APS/SBC-CAT beamline 19ID, and at CLS/CMCF beamline 08ID for SMN-compound 4, UHRF1-compound 1 and TDRD3-compound 1, respectively. The fluorescence polarization assays were performed by using the Synergy 2 microplate reader (BioTek). All the isothermal titration calorimetry measurements were performed by using a VP-ITC (MicroCal, Inc.), an iTC-200 (MicroCal, Inc.) or a Nano-ITC (TA, Inc.) microcalorimeter. Fluorescence and immunofluorescence data were obtained by using Zeiss LSM880 microscopy and Nikon C2+ confocal microscope, respectively. The AP-MS samples were analyzed by nanoLC-MS/MS (nanoRSLC, ThermoFisher) and Orbitrap Eclipse (Thermo Fisher).
Data analysis	The program PHASER was used for molecular replacement (MR) when needed. Models were interactively rebuilt, refined and validated using COOT, REFMAC and MOLPROBITY software, respectively. MarvinSketch (Chemaxon.com) was used for the calculation of some SMILES strings during preparation of small molecule geometry restraints. PDB_EXTRACT and CCTBX library were used during preparation of the crystallographic models for PDB deposition and publication. DENZO, SCALEPACK, AIMLESS, XDS, PARVATI server, COMBAT, POINTLESS were also used for structure determination. GRADE server, PRODRG, ELBOW were used for preparation of geometry restraints for compound 1, compound 4, and compound 6, respectively. The fluorescence polarization data were analyzed by GraphPad Prism version 5 software. The isothermal titration calorimetry data were fitted using the single-site binding model within the Origin software 7.0 package (MicroCal, Inc.) or the independent model within the Nano-Analyze software package (TA, Inc.). The data of AP-MS were analyzed by MaxQuant software (version 1.6.1.1) and the volcano plot was prepared by using OriginPro 9.0. For the immunofluorescence data, the NIS-elements AR software (Nikon) and Photoshop (Adobe) were used.

For manuscripts utilizing custom algorithms or software that are central to the research but not yet described in published literature, software must be made available to editors and reviewers. We strongly encourage code deposition in a community repository (e.g. GitHub). See the Nature Portfolio guidelines for submitting code & software for further information.

Data

Policy information about availability of data

All manuscripts must include a data availability statement. This statement should provide the following information, where applicable:

- Accession codes, unique identifiers, or web links for publicly available datasets
- A description of any restrictions on data availability
- For clinical datasets or third party data, please ensure that the statement adheres to our policy

The coordinates and structure factors generated in this study have been deposited in the Protein Data Bank (PDB) with accession codes 4QQ6 [https://www.rcsb.org/structure/4QQ6] (SMN-compound 1), 4QQD [https://www.rcsb.org/structure/4QQD] (UHRF1-compound 1), 7W2P [https://www.rcsb.org/structure/7W2P] (SMN-compound 4), 7W30 [https://www.rcsb.org/structure/7W30] (SMN-compound 6), 6V9T [https://www.rcsb.org/structure/6V9T] (TDRD3-compound 1). The mass spectrometry proteomics data generated in this study have been deposited to the ProteomeXchange Consortium via the iProX partner repository with the dataset identifier PXD034927 [http://proteomecentral.proteomexchange.org/cgi/GetDataset?ID=PXD034927] (Identification of the target proteins for compound 1 in U2OS cells). The structural data used in this study are available in the Protein Data Bank (PDB) under accession codes 1MHN [https://www.rcsb.org/structure/1MHN] (SMN Tudor domain structure), 3DB3 [https://www.rcsb.org/structure/3DB3] (UHRF1-H3K9me3 complex), 3PMT [https://www.rcsb.org/structure/3PMT] (TDRD3 Tudor domain structure), 3ASK [https://www.rcsb.org/structure/3ASK] (UHRF1-H3K9me3 complex), 5YYA [https://www.rcsb.org/structure/5YYA] (UHRF1 bound to ethylene glycol). The uncropped and unprocessed versions of blots, all original ITC curves, NMR spectra of synthesized CCVJ and biotin conjugated compound 1 (CCVJ-Cmpd 1 and biotin-Cmpd 1) generated in this study are provided in the Supplementary Information/Source Data file.

Human research participants

Policy information about studies involving human research participants and Sex and Gender in Research.

Reporting on sex and gender	n/a
Population characteristics	n/a
Recruitment	n/a
Ethics oversight	n/a

Note that full information on the approval of the study protocol must also be provided in the manuscript.

Field-specific reporting

Please select the one below that is the best fit for your research. If you are not sure, read the appropriate sections before making your selection.

☒ Life sciences ☐ Behavioural & social sciences ☐ Ecological, evolutionary & environmental sciences

For a reference copy of the document with all sections, see nature.com/documents/nr-reporting-summary-flat.pdf

Life sciences study design

All studies must disclose on these points even when the disclosure is negative.

Sample size	Based pilot studies, experiments were performed at 2-3 times to verify the reproducibility of the experimental findings. The exact sample size of the associated experiments was described in the Method and Figure legends.
Data exclusions	No data were excluded.
Replication	Each assay was performed at 2-3 times. All the attempts at replication were successful.
Randomization	Randomization was not considered within the experimental samples tested in this study, since cell lines used were generally identical.
Blinding	Blinding was not considered within the experimental samples tested in this study, since data generated in this study was not practical and no subjective results were gathered.

Reporting for specific materials, systems and methods

We require information from authors about some types of materials, experimental systems and methods used in many studies. Here, indicate whether each material, system or method listed is relevant to your study. If you are not sure if a list item applies to your research, read the appropriate section before selecting a response.

Materials & experimental systems

n/a	Involved in the study
	☒ Antibodies
	☒ Eukaryotic cell lines
☒	☐ Palaeontology and archaeology
☒	☐ Animals and other organisms
☒	☐ Clinical data
☒	☐ Dual use research of concern

Methods

n/a	Involved in the study
☒	☐ ChIP-seq
☒	☐ Flow cytometry
☒	☐ MRI-based neuroimaging

Antibodies

Antibodies used

Primary antibodies:
SMN: RRID: AB_397973, BD Transduction Laboratories, 610646, mouse monoclonal antibody, clone number 8/SMN; RRID: AB_2193083, Santa Cruz Biotechnology, sc-15320, rabbit polyclonal antibody
TDRD3: Cell signaling, 5492, rabbit monoclonal antibody, clone number 5942
SND1: RRID: AB_10631268, Bethyl, A302-883A, rabbit polyclonal antibody
RFP: RRID: AB_945213, Abcam, ab62341, rabbit polyclonal antibody
GFP: RRID: AB_627695, Santa Cruz Biotechnology, sc-9996, mouse monoclonal antibody, clone number GFP B-2; RRID: AB_2536526, Invitrogen, G10362, recombinant rabbit monoclonal antibody
POLR2A: RRID: AB_304868, Abcam, ab5408, mouse monoclonal antibody, clone number 4H8
ACTB: RRID: AB_476744, Sigma, A5441, mouse monoclonal antibody, clone number AC-15
TUBB: RRID: AB_2241191, Santa Cruz Biotechnology, sc-9104, rabbit polyclonal antibody
S9.6 antibody for R-loop (Anti-DNA-RNA Hybrid [S9.6] Antibody): RRID: AB_2687463, Kerafast, ENH001, mouse monoclonal antibody
anti-RNase H1: RRID: AB_2238624, Proteintech, 15606-1-AP

Secondary antibodies:
horseradish peroxidase-conjugated goat anti-mouse secondary antibody: RRID: AB_2338512, Jackson Immuno Research, 115-035-174
horseradish peroxidase-conjugated mouse anti-rabbit secondary antibody: RRID: AB_2339149, Jackson Immuno Research, 211-032-171
goat anti-mouse Alexa Fluor 488 antibody: RRID: AB_2534069, Thermo Fisher, A11001
goat anti-rabbit Alexa Fluor 568, RRID: AB_143157, Thermo Fisher, A11011

Validation

All the antibodies used in this study were obtained from commercial companies and have been registered with a unique Research resource identifier (RRID). Validation of these antibodies are accessible on companies' websites or RRID portal and all the antibodies were verified by relative expression to ensure that the antibody binds to the antigen stated.

Eukaryotic cell lines

Policy information about cell lines and Sex and Gender in Research

Cell line source(s)	U2OS cells (ATCC, HTB-96) and HEK293 cells (ATCC, CRL-1573) were obtained from ATCC.
Authentication	The cell lines were authenticated by short tandem repeat (STR) profile method.
Mycoplasma contamination	The cell lines were tested negative for mycoplasma contamination by PCR.
Commonly misidentified lines (See ICLAC register)	No commonly misidentified line was used in this study.
